# SARS-CoV-2 Main Protease Inhibitors That Leverage Unique Interactions with the Solvent Exposed S3 Site of the Enzyme

**DOI:** 10.1021/acsmedchemlett.4c00146

**Published:** 2024-05-20

**Authors:** Lauren R. Blankenship, Kai S. Yang, Veerabhadra R. Vulupala, Yugendar R. Alugubelli, Kaustav Khatua, Demonta Coleman, Xinyu R. Ma, Banumathi Sankaran, Chia-Chuan D. Cho, Yuying Ma, Benjamin W. Neuman, Shiqing Xu, Wenshe Ray Liu

**Affiliations:** † Texas A&M Drug Discovery Center and Department of Chemistry, College of Arts and Scienes, Texas A&M University, College Station, Texas 77843, United States; ‡ Molecular Biophysics and Integrated Bioimaging, Berkeley Center for Structural Biology, Laurence Berkeley National National Laboratory, Berkeley, California 94720, United States; § Department of Biology, College of Arts and Sciences, Texas A&M University, College Station, Texas 77843, United States; ∥ Texas A&M Global Health Research Complex, Texas A&M University, College Station, Texas 77843, United States; ⊥ Department of Molecular Pathogenesis and Immunology, School of Medicine, Texas A&M University, College Station, Texas 77843, United States; # Department of Pharmaceutical Sciences, Irma Lerma Rangel School of Pharmacy, Texas A&M University, College Station, Texas 77843, United States; ¶ Institute of Biosciences and Technology and Department of Translational Medical Sciences, School of Medicine, Texas A&M University, Houston, Texas 77030, United States; ◆ Department of Biochemistry and Biophysics, College of Agriculture and Life Sciences, Texas A&M University, College Station, Texas 77843, United States; ○ Department of Cell Biology and Genetics, School of Medicine, Texas A&M University, College Station, Texas 77843, United States

**Keywords:** COVID-19, SARS-CoV-2, Main Protease, S3 Site, Tail Flip

## Abstract

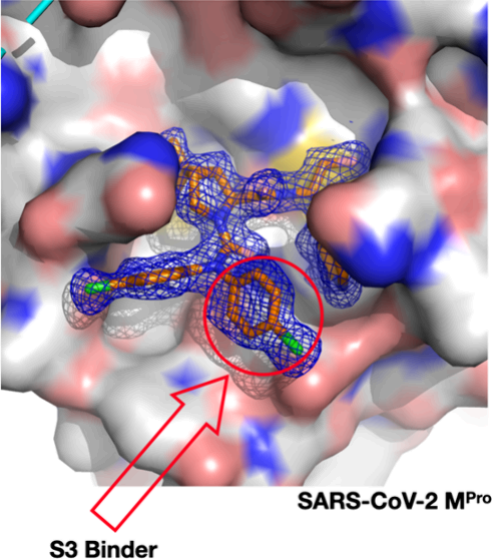

The main protease (M^Pro^) of SARS-CoV-2 is crucial for the virus’s replication and pathogenicity. Its active site is characterized by four distinct pockets (S1, S2, S4, and S1–3′) and a solvent-exposed S3 site for accommodating a protein substrate. During X-ray crystallographic analyses of M^Pro^ bound with dipeptide inhibitors containing a flexible *N*-terminal group, we often observed an unexpected binding mode. Contrary to the anticipated engagement with the deeper S4 pocket, the *N*-terminal group frequently assumed a twisted conformation, positioning it for interactions with the S3 site and the inhibitor component bound at the S1 pocket. Capitalizing on this observation, we engineered novel inhibitors to engage both S3 and S4 sites or to adopt a rigid conformation for selective S3 site binding. Several new inhibitors demonstrated high efficacy in M^Pro^ inhibition. Our findings underscore the importance of the S3 site’s unique interactions in the design of future M^Pro^ inhibitors as potential COVID-19 therapeutics.

The severe acute respiratory syndrome coronavirus 2 (SARS-CoV-2) is the causative agent of coronavirus disease 2019 (COVID-19), which led to a pandemic and continues to sustain a significant number of active cases.^1,2^ It is a positive-sense RNA virus and enters human cells using its Spike protein, which binds to the ACE2 receptor.^3,4^ After entry, SARS-CoV-2 releases its RNA. This genomic RNA is then translated into two large polypeptides, pp1a and pp1ab, a process facilitated by the host’s translation machinery.^5,6^ These polypeptides are subjected to autocatalytic cleavage by two protease domains within them, yielding essential nonstructural proteins (nsps).^6^ Some nsps form an RNA polymerase complex, which replicates both genomic and subgenomic RNAs. The two protease domains responsible for this cleavage are the main protease (M^Pro^) and the papain-like protease (PL^Pro^).^7^ Given their critical role in the virus’s life cycle and pathogenesis, both proteases are prime targets for antiviral drug development against SARS-CoV-2, with the majority of research efforts focusing on M^Pro^.^8−11^ Notable successes in the M^Pro^ inhibitor development have led to the approval of nirmatrelvir for its combined use with ritonavir and ensitrelvir for the treatment of COVID-19.^12,13^

M^Pro^ is a cysteine protease featuring a catalytic dyad composed of His41 and Cys145, with the latter serving as the catalytic residue. Cys145 forms a thioester intermediate with protein substrates during the catalytic process.^14^ According to the Schechter-Berger nomenclature, the active site of M^Pro^, as depicted in Figure 1A, can be divided into distinct subsites as S1, S2, S3, S4, and S1–3′, which bind P1, P2, P3, P4, and P1–3′, respectively, in protein substrates.^15^ S1, S2, S4, and S1–3′ are deep, concave pockets that are predominantly hydrophobic.^11^ The S3 subsite, in contrast, is exposed to the solvent and comprises a shallow, flat surface created by the backbone of Leu167 and the entire residue of Glu166. Interestingly, active site-targeting M^Pro^ inhibitors, including nirmatrelvir and ensitrelvir, predominantly feature hydrophobic moieties that interact with the S3 site.^12,13^ Nirmatrelvir interacts with all subsites of M^Pro^, with the exception of S1–3′.^12,16^ However, during our structural determination of M^Pro^ in complex with GC376 (the chemical structure of GC376 is presented in Figure S1), which is a dipeptide featuring an *N*-terminal benzyloxycarbonyl (CBZ) group, we observed that the CBZ phenyl group occupies the S3 site, contrary to the expectation of its binding to the S4 site. Shown in Figure 1B, the 2Fo-Fc map distinctively delineates all of the structural elements of GC376 in the M^Pro^ active site. This includes the covalent hemithioacetal linkage with Cys145, the P1 lactam anchored in the S1 site, the P2 leucine situated in the S2 site, and the CBZ phenyl group at S3, which is stacked above Glu166. Adjacent to this, we identified residual difference electron density in the S4 site (Figure S2). However, this density was disconnected from the 2Fo-Fc electron density surrounding GC376 and corresponded more closely to DMSO, the solvent used to dissolve GC376. The wild-type M^Pro^-GC376 complex structures has been reported over ten times.^17−25^ In all but two instances, the CBZ phenyl group is localized at the S3 site. The two exceptions that show the CBZ phenyl group at the S4 site present weak 2Fo-Fc electron density surrounding the CBZ phenyl group, suggesting a variable and flexible binding mode.^23,25^

**Figure 1:**
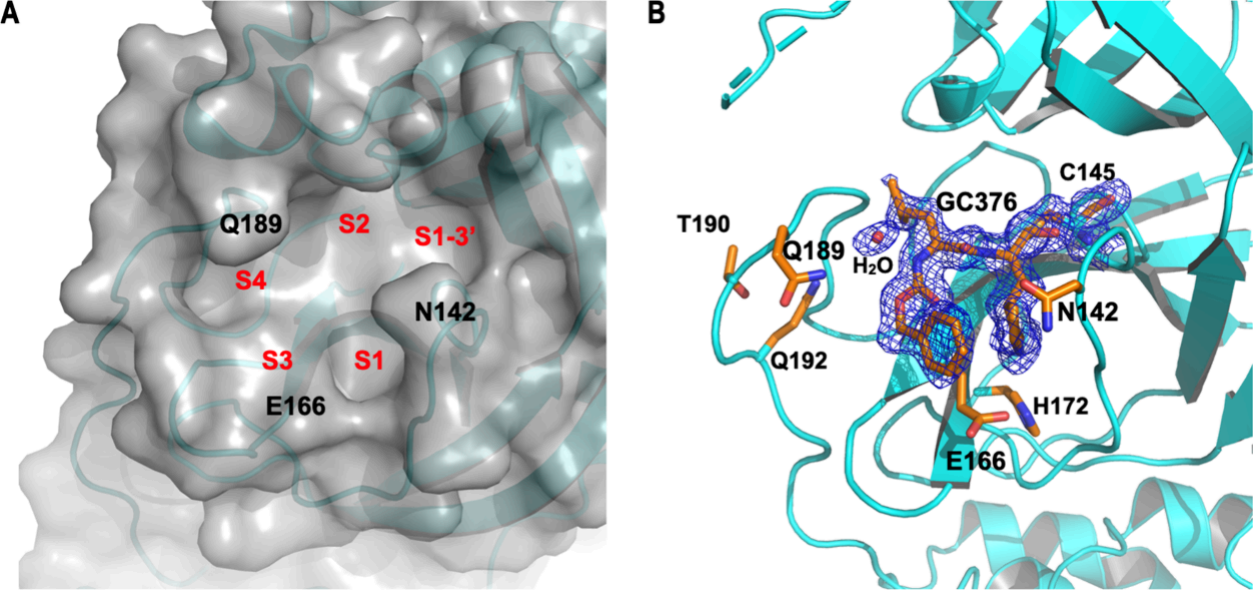
M^Pro^ active site and its interaction with GC376. (A) The M^Pro^ active site showcasing S1, S2, S3, S4, and S1–3′ subsites for binding a protein substrate. The protein is presented in the surface mode. The configuration of the structure is based on PDB entry 7TE0. (B) The crystal structure of M^Pro^ in complex with the inhibitor GC376. The blue 2Fo-Fc map delineates the electron density around GC376 and the catalytic residue C145, contoured at the 1s level for clarity.

The S4 site of M^Pro^ is predominantly a hydrophobic binding pocket, whereas the S3 site is solvent-exposed with a flat binding surface.^26^ Therefore, the configuration in which the CBZ phenyl group of GC376 binds at the S3 site piqued our interest. In the structure that we determined, the electron density surrounding the CBZ phenyl group was well-defined, signifying strong binding at this location. Two M^Pro^ residues, Asn142 and Glu166, are positioned in proximity to the CBZ phenyl group. Notably, while the alkyl side chain of Glu166 engages characteristic hydrophobic interactions with the CBZ phenyl group, the van der Waals contacts between the CBZ phenyl group and the side chain amide of Asn142 or the side chain carboxylate of Glu166 are unusual. This is because desolvation of the carboxylate or amide in these residues is required for their interactions with the CBZ phenyl group of GC376. A plausible explanation for this binding is that the substantial van der Waals forces between the P1 lactam and the CBZ phenyl group offset the energy costs incurred by desolvation. Indeed, in the finally determined structure, the CBZ phenyl group is neatly positioned as a cap over the P1 lactam nestled in the S1 pocket, facilitating interactions with Asn142 and Glu166. Dipeptide M^Pro^ inhibitors with their *N*-terminal group unambiguously bound at the S4 site have been reported. However, these inhibitors contain an *N*-terminal group with a fixed conformation that does not allow its flipping to the S3 site.^11,27,28^ Nirmatrelvir is a tripeptide inhibitor with a P3 *tert*-butyl-glycine. The P3 residue binds to M^Pro^ at the supposed S3 site but does not extend to interact with the P1 lactam bound at the S1 site.^16^

Intrigued by the unique binding observed with GC376, we embarked on elucidating the crystal structures of M^Pro^ in complex with a series of dipeptide inhibitors, each featuring a flexible *N*-terminal group. The X-ray crystallography analysis followed our established soaking strategy that has facilitated the structural determinations of more than 30 M^Pro^-inhibitor complexes.^29−31^ MI-14, MI-30, and MI-31 are aldehyde-based covalent inhibitors of M^Pro^ that were developed by Qiao et al. and showed high antiviral potency.^10^ Their chemical structures are shown in Figure S1. These compounds are dipeptidyl in nature and each contains a flexible *N*-terminal group. We determined the structures of these inhibitors in complex with M^Pro^, and Figure 2A depicts the superimposed structures of these molecules in the active site of M^Pro^. 2Fo-Fc electron density maps around three inhibitors bound at the M^Pro^ active site are presented in Figures S3–5 as well. Different conformations for the Asn142 and Gln189 side chains were observed in the three structures, but overall, the enzyme’s structure remains constant across all three complexes. MI-14 and MI-30 share a (2,4-dichlorophenoxy)acetyl group at their *N*-terminus but are different at the P2 residue. The presence of the 2-chloro substituent in the two molecules causes the phenyl group to deviate from the position held by the CBZ phenyl group in the M^Pro^-GC376 complex due to steric clash, yet it still occupies the S3 site. MI-31, structurally similar to MI-30 but with a (3,4-dichlorophenoxy)acetyl group at the *N*-terminus, demonstrates that relocating the 2-chloro to the 3-position on the phenyl ring mitigates steric clash, allowing the phenyl group to assume a position similar to the CBZ phenyl group in the M^Pro^-GC376 complex. In the M^Pro^-MI-31 complex, the dual chloride substituents that stack atop the P1 lactam push the side chain of Asn142 to rotate about 75° clockwise.

**Figure 2:**
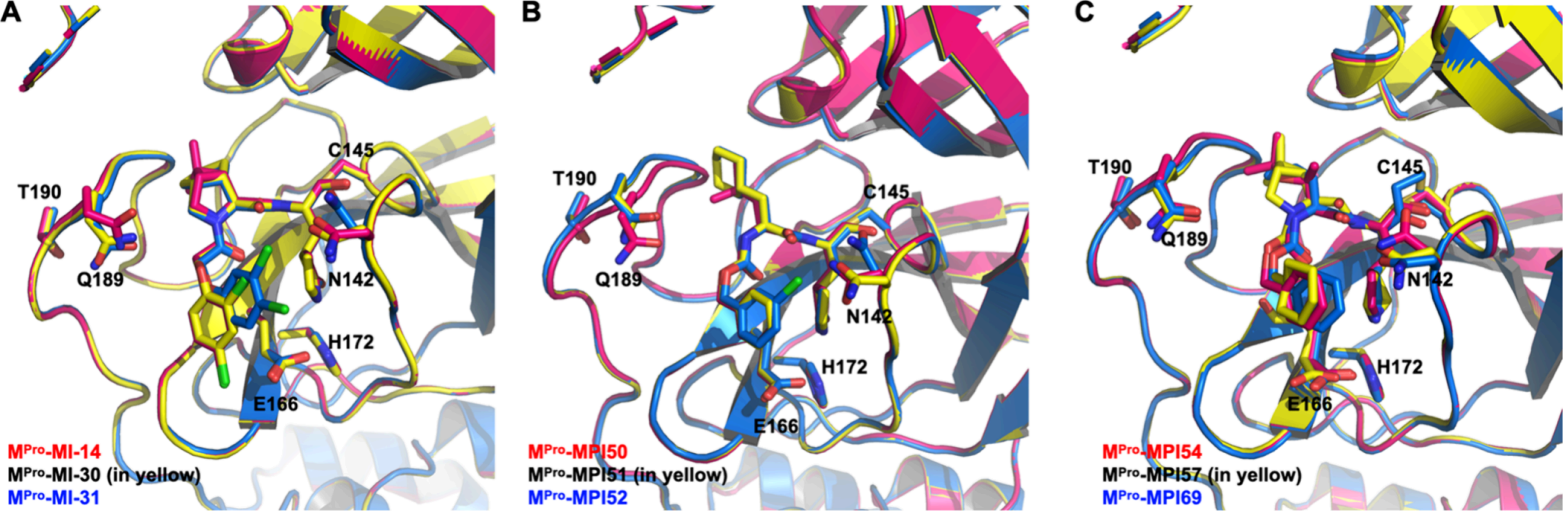
Comparative crystal structure overlays of M^Pro^-inhibitor complexes. Panels depict M^Pro^ in complex with (A) inhibitors MI-14, MI-30, and MI-31; (B) inhibitors MPI50, MPI51, and MPI52; and (C) inhibitors MPI54, MPI57, and MPI69. Inhibitors and side chains for residues including Asn142, Cys145, Glu166, His172, Gln189, and Thr190 are presented in stick representation. Carbon atoms in all structures are color-coded to match the corresponding inhibitor names.

We recently published a group of dipeptide inhibitors of M^Pro^ with an aldehyde functional group that acts as a covalent warhead and demonstrated inhibitors characterized by a bulky P2 spiro residue exhibiting promising antiviral potency.^32^ Efforts to elucidate the structures of their complexes with M^Pro^ were successful for MPI50, MPI51, MPI52, MPI54, and MPI57. In addition, we have developed a range of azapeptide inhibitors with various covalent moieties to target the M^Pro^ catalytic cysteine.^33^ Among these, MPI69 is a dipeptide inhibitor with a flexible *N*-terminal group. Its M^Pro^ complex structure were resolved as well. 2Fo-Fc electron density maps around all inhibitors in the M^Pro^ active site are presented in Figures S6–11. Chemical structures of these inhibitors can be found in Figure S1. Shown in Figure 2B, the superimposed structures of MPI50, MPI51, and MPI52 at the active site of M^Pro^ revealed a consistent *N*-terminal binding pattern akin to that of the CBZ phenyl group in the M^Pro^-GC376 complex. In particular, the 3-chloro substituent of MPI52, which caps the P1 lactam bound in the S1 site, prompts the Asn142 side chain rotating clockwise for about 75°. This observation is similar to that made in the M^Pro^-MI-31 complex. Figure 2C presents the overlay of MPI54, MPI57, and MPI69 bound to the M^Pro^ active site. All three inhibitors exhibit a similar *N*-terminal binding configuration at the S3 site, in line with the previous described M^Pro^-dipeptide inhibitor complexes, despite that MPI57 contains a cyclohexyl substituent in its *N*-terminal group as opposed to the CBZ phenyl group. The majority of all determined structures shown in Figure 2 also feature a distinctive Y-shaped, S–O–N–O-S-bridged cross-link formed among three residues Cys22, Cys44, and Lys61, corroborating earlier reports.^34,35^

Given that a flexible *N*-terminal group in a dipeptide inhibitor typically occupies the S3 site of the M^Pro^ enzyme, we hypothesized that a dipeptide inhibitor featuring an *N*-terminal group capable of engaging both the S3 and S4 sites could exhibit an affinity stronger than that of one interacting solely with the S3 site. To investigate this hypothesis, we embarked on designing, synthesizing, and evaluating novel inhibitors, as detailed in Table 1. Our initial series of inhibitors were derived from MPI57, which has an IC_50_ value of 0.025 μM against M^Pro^.^32^ To extend the reach to the S4 site, we introduced an additional phenyl group to the benzylic carbon of the CBZ group in MPI57, yielding compound MPI94. However, this alteration resulted in decreased potency with an IC_50_ value of 0.096 μM. Attaching two more fluorine atoms produced a marginally better inhibitor, MPI95, with an IC_50_ value of 0.094 μM. Utilizing a bis(4-chlorophenyl)acetyl group, in which the carbamate oxygen is removed, we synthesized MPI96. MPI96 demonstrated weak inhibition, with an IC_50_ value of 0.28 μM. Replacing the methine CH in MPI96’s *N*-terminal group with nitrogen led to an even less effective inhibitor, MPI97, which has an IC_50_ value of 0.45 μM. MPI94, MPI95, MPI96, and MPI97 all have a rigid biphenyl group with limited flexibility to engage both S3 and S4 sites. This may contribute to their low potency. Aiming for increased interaction with both the S3 and S4 sites through enhanced flexibility, we introduced two oxygen atoms into the *N*-terminal group of MPI96, creating MPI98. However, MPI98 displayed an IC_50_ value identical to that of MPI96. Given that the side chains of phenylalanine and cyclohexylalanine structurally mimic the *N*-terminal group seen in GC376 and other structures depicted in Figure 2, we also synthesized tripeptide inhibitors MPI99 and MPI100, featuring an *N*-terminal CBZ group targeting the S4 site. Yet, these compounds exhibited inhibition potency comparable to MPI96. Observing that the *N*-terminal CBZ phenyl group of MPI57 is within van der Waals distance to the side chain carboxylate of Glu166, we speculated that substituting a *meta* carbon in the CBZ phenyl group with nitrogen to form a pyridine group could enable a charge–charge interaction with the Glu166 side chain carboxylate. Pyridine has a p*K*_a_ value of 5.2. When it is positioned close to a carboxylate, proton transfer between the two groups will potentially occur to generate a ion pair. This rationale led to the development of MPI102. Although the introduction of a pyridine is expected to increase water solubility, MPI102 demonstrated an IC_50_ value similar to MPI57, suggesting that a charge–charge interaction does occur between the pyridine group of MPI102 and the Glu166 side chain carboxylate to counteract the energy loss during desolvating the pyridine group.

**Table 1:**
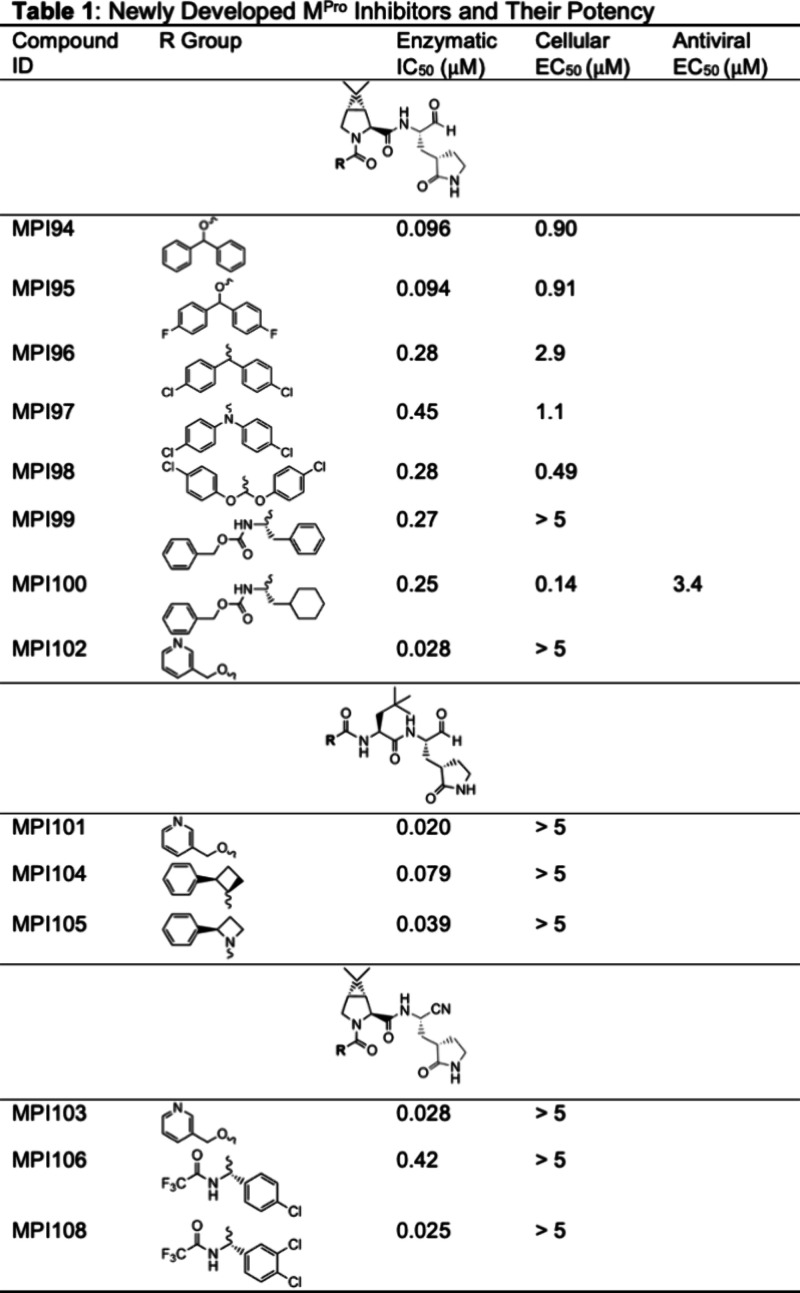
Newly Developed M^Pro^ Inhibitors and Their Potency

Our second series of inhibitors were derived from MPI50 that has a determined IC_50_ value of 0.053 μM.^32^ Informed by the development of MPI102, we introduced a corresponding *N-*terminal pyridine group, resulting in MPI101. As expected, this modification led to MPI101 exhibiting superior potency compared to its progenitor compound MPI50, achieving an IC_50_ value of 0.020 μM. Recognizing that a flexible *N*-terminal group may suffer entropy loss when adopting the conformation to bind the S3 site, we proposed that a strategically imposed rigidity might mitigate this effect by preorienting the group for S3 site interaction, thus potentially enhancing binding affinity to M^Pro^. With this concept in mind, we synthesized MPI104 and MPI105. Both feature a conformationally constrained four-membered ring designed to anchor the connected phenyl group in the S3 binding pose. These compounds demonstrated robust inhibition of M^Pro^, with MPI105 outperforming MPI50, which is reflected in its lower IC_50_ value of 0.039 μM.

The third series of compounds were derived from nirmatrelvir. Nirmatrelvir is a tripeptide M^Pro^ inhibitor known for its ability to bind both the S3 and S4 sites of the M^Pro^ enzyme.^12^ It contains a nitrile warhead and a P1 side chain lactam. On a separate effort, we attempted to generate dipeptide M^Pro^ inhibitors with the nitrile warhead while retaining the same PI lactam and analogous P2 residue found in nirmatrelvir. However, all inhibitors developed had low potency with IC_50_ values exceeding 1 μM.^32^ Observing the high potency provided by a pyridine *N*-terminal tail in two of our dipeptide M^Pro^ inhibitors, we designed and synthesized MPI103, which contains both a nitrile warhead and a pyridine tail. This compound indeed exhibited strong inhibition activity against M^Pro^, with an impressive IC_50_ value of 0.028 μM. To preserve interactions that were observed for the 3,4-dichlorophenyl group in MI-31 to engage the S3 site and the *N*-terminal trifluoroacetamide in nirmatrelvir to engage the S4 site, we designed and synthesized MPI106 and MPI108. Both compounds share a P3 phenylglycine backbone, and MPI108 incorporates the same 3,4-dichloro substituents as those found in MI-31. While MPI106 display low M^Pro^ inhibition potency, MPI108 demonstrated superior potency with an IC_50_ value of 0.025 μM. It is notably more potent than nirmatrelvir, which has an IC_50_ value of 0.066 μM under similar assay conditions.^30^

To understand the newly synthesized molecules in their interactions with M^Pro^, we conducted X-ray crystallography analysis of their complexes with M^Pro^. We successfully determined the structures for the five inhibitors MPI94, MPI95, MPI97, MPI101, and MPI105. 2Fo-Fc electron density maps around the five inhibitors in the active site of M^Pro^ are presented in Figures S12–S16. As depicted in Figure 3A, an overlay of three inhibitors, MPI94, MPI95, and MPI97, shows how their branched *N*-terminal groups interact at the active site of M^Pro^. M^Pro^, with the exception of the aa46–51 region and Asn142, maintains consistent conformations across all complexes. In the M^Pro^-MPI97 complex, the aa46–51 region exhibits an unstructured conformation, facilitating the formation of a unique Y-shaped, S-O-N-O-S-bridged cross-link involving residues Cys22, Cys44, and Lys61.^34,35^ Notably, in the M^Pro^-MPI94 and M^Pro^-MPI95 complexes, Asn142, at its side chain, undergoes a clockwise rotation of approximately 90° from its position in the wild-type enzyme. Despite some subtle variations in their branched *N*-terminal groups, all three inhibitors demonstrate a similar binding mode at the S3 site, while exhibiting different interaction modes at the S4 site, suggesting a pronounced preference for interaction at the S3 site. In the case of MPI94, the phenyl group at the S4 site engages van der Waals interactions, being stacked between Pro168 and Gln189. However, electron density around the C2, C3, C5, and C6 positions of this phenyl group is notably absent, suggesting potential free rotation around its C1–C4 axis. Being structurally similar to MPI94, MPI95 exhibits similar binding modes to both S3 and S4 sites. Notably, the fluorophenyl group at the S4 site in MPI95 displays electron density at C1, C4, and the 4-fluoro positions, but weaker density at other positions, indicating free rotation around the C1–C4 axis. MPI97 has an *N*-terminal bis(4-chlorophenyl)urea group. Compared with the branching points observed in MPI94 and MPI95, the branching point in this *N*-terminal group is positioned closer to the amino group in the P2 residue. This results in the elevation of the S4-binding 4-chlorophenyl group, as depicted in Figure 3A, which in turn exerts upward pressure on Gln189. This close proximity restricts the S4-binding 4-chlorophenyl group to adopt a fixed conformation that clearly shows electron density around all non-hydrogen atoms in the determined structure. It is this close interaction with Gln189, which apparently generates steric hindrance, that likely explains why MPI97 serves as a weaker inhibitor compared to MPI94 and MPI95.

**Figure 3:**
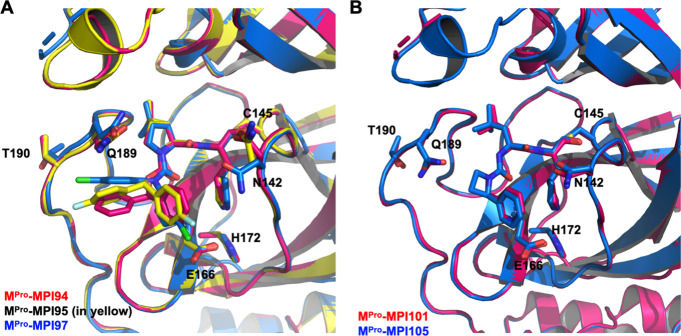
Comparative crystal structure overlays of M^Pro^ in complex with newly developed inhibitors. Panels depict M^Pro^ in complex with (A) inhibitors MPI94, MPI95, and MPI97; and (B) inhibitors MPI102 and MPI105. Inhibitors and side chains of key residues are presented in a stick representation. Carbon atoms in all structures are color-coded to match the corresponding inhibitor names.

MPI101 and MPI105 were crafted based on the structure of MPI50, featuring a P1 side chain lactam to interact with the S1 site and a P2 α-*t*-butylalanine to engage the S2 site.^32^ The superimposition of the two determined inhibitor-M^Pro^ complexes, as depicted in Figure 3B, shows that the two structures at the M^Pro^ active site are almost identical except that the M^Pro^-MPI101 complex has a flexible aa46–51 region. MPI101 is different from MPI50 only in its pyridine nitrogen. The introduction of this pyridine nitrogen was aimed at establishing a potent charge–charge interaction with the Glu166 side chain carboxylate, a conjecture supported by the lowest determined *K*_d_ value for MPI101 among all newly developed inhibitors. Despite expectations of the pyridine nitrogen being drawn closer to the Glu166 side chain carboxylate due to this interaction, the determined structure reveals its position to be the same as its counterpart carbon in the CBZ group of MPI50. This suggests robust van der Waals interactions anchoring the pyridine at the S3 site. Compared to MPI101, MPI105 has a constrained *N*-terminal group secured by the azetidine moiety. Figure 3B shows a perfect alignment of MPI105’s *N*-terminal phenyl group at the S3 site, with the azetidine group snugly fitting into active site without any steric clash with neighboring residues, including Gln189. For both complexes, there is additional difference electron density at the S4 site attributing to DMSO. Considering the remarkable potency of both MPI101 and MPI105 as M^Pro^ inhibitors, devising a strategy to maintain their robust binding to the S3 site while extending their reach to the S4 site through the introduction of additional chemical moieties necessitates astute designs. This aspect is definitely worth exploring.

When recombinantly expressed in human cells, M^Pro^ triggers acute toxicity, leading to the killing of host cells. Built upon this observation, we previously developed a cell-based assay to evaluate the cellular potency of M^Pro^ inhibitors.^36^ In this assay, inhibitors capable of permeating into cells to effectively inhibit M^Pro^ prevent toxicity induced by an M^Pro^-eGFP (enhanced green fluorescent protein) fusion protein transiently expressed in 293T cells. Consequently, this inhibition fosters host cell survival and augments the overall expression of M^Pro^-eGFP that can be quantified via flow cytometry. This assay expedites the potency assessment of M^Pro^ inhibitors in human cells by circumventing high-biosafety-restricted antiviral characterizations that need to be conducted in a BSL3 lab setup. Using this assay, we systematically characterized a diverse array of M^Pro^ inhibitors, both repurposed and newly developed, prior to advancing inhibitors with notable cellular potency to more biosafety-restricted antiviral potency evaluations. Utilizing this assay, we determined the cellular EC_50_ values for all newly developed molecules. As shown in Table 1, with the exception of MPI94, MPI95, MPI97, MPI98, and MPI100, which demonstrated a cellular EC_50_ value around or below 1 μM, the majority of tested compounds exhibited modest cellular potency, with the most with cellular EC_50_ value above 5 μM. Given the lack of comparable cellular potency exhibited by the majority of newly developed inhibitors in this work to previously reported highly potent M^Pro^ inhibitors such as MPI50 and MPI60 and MPI61, we did not conduct antiviral potency assessments on most of them. Only MPI100, showcasing a cellular EC_50_ value of 0.14 μM, was advanced to antiviral testing in human A549 cells using the SARS-CoV-2 delta variant (hCoV-19/USA/MD-HP05647/2021). MPI100 has a determined antiviral EC_50_ value of 3.4 μM. All pyridine-containing inhibitors have low cellular potency, potentially due to the pyridine influence on cellular permeability. The potential positive charge might play a role to attract these molecules to the negatively charged cell membrane, but this strong interaction prevents the molecules from moving into cells. It is also likely the introduced rigidity might also decrease cellular permeability for other compounds.

In summary, during our X-ray crystallography analysis of the M^Pro^-GC376 complex, we observed a distinctive conformational arrangement of GC376 at its flexible *N*-terminal group that adopts a flipped tail conformation to bind specifically at the S3 site of M^Pro^. This observation remained constant across 9 other dipeptide inhibitors with a flexible *N*-terminal group, indicating a strong preference of maintaining the flexible *N*-terminal group in dipeptide inhibitors at the S3 site of M^Pro^. Capitalized on this unique interaction, we devised additional inhibitors. Three of these inhibitors feature an *N*-terminal pyridine moiety to potentially engage the Glu166 side chain carboxylate for a charge–charge interaction. As a result, these inhibitors demonstrate exceptional potency in inhibiting M^Pro^*in vitro*. Moreover, the design of introducing a conformation constraint to lock the *N*-terminal group at the preferred S3 binding mode was also successful in making MPI105 better than its parent compound MPI50. Furthermore, the integration of both the potent S2 binding group from MI-31 and the S4 binding group from nirmatrelvir into the inhibitor MPI108 resulted in better *in vitro* inhibition potency than nirmatrelvir. By combining our findings with optimizations aimed at improving *in vivo* efficacy, we believe that our insights into the preferential binding of an inhibitor component to the S3 site, the enhancement of binding strength at the S3 site through an introduced charge–charge interaction, and the integration of both S3 and S4 binders for improved overall M^Pro^ binding are invaluable for the future design of M^Pro^ inhibitors. We highly recommend a continued exploration in this direction.

## Supporting Information Available

The expression and crystallization of M^Pro^; the structural determination of M^Pro^ bound with all inhibitors; the synthesis and characterization of all newly developed inhibitors; the determination of enzymatic IC_50_, cellular EC_50_ to inhibit ectopically expressed M^Pro^ in 293T cells, and antiviral EC_50_ to inhibit SARS-CoV-2 infection of A549-hACE2 cells for newly developed inhibitors; Figure S1 shows the chemical structures of all inhibitors; Figures S2–16 that show electron density around 15 inhibitors bound at the active site of M^Pro^; Table S1 displays all parameters for the X-ray crystallography analysis of M^Pro^ bound with different inhibitors; Finally determined structures have been deposited into the Protein Data Bank with the entry codes as 8TY5 (M^Pro^-MI-14), 8TY4 (M^Pro^-MI-30), 8TY3 (M^Pro^-MI-31), 8TQH (M^Pro^-MPI50), 8TQU (M^Pro^-MPI51), 8TQT (M^Pro^-MPI52), 8TQL (M^Pro^-MPI54), 8TQJ (M^Pro^-MPI57), 8U9H (M^Pro^-MPI64), 7SHD (M^Pro^-MPI68), 7SHC (M^Pro^-MPI69), 8U9K (M^Pro^-MPI94), 8U9M (M^Pro^-MPI95), 8U9N (M^Pro^-MPI96), 8U9T (M^Pro^-MPI97), 8U9 V (M^Pro^-MPI98), 8U9 V (M^Pro^-MPI101), 8U9W (M^Pro^-MPI105) (PDF) brief description (PDF)

## ABBREVIATIONS

COVID-19: coronavirus disease 2019
SARS-CoV-2: severe acute respiratory syndrome coronavirus 2
M^Pro^: main protease
IC_50_: half maximal inhibitory concentration
EC_50_: half maximal effective concentration.
