# Supplementary material for: SARS-CoV-2 Main Protease Inhibitors That Leverage Unique Interactions with the Solvent Exposed S3 Site of the Enzyme
Source: ACS Med Chem Lett. 2024 May 20;15(6):950–7. doi: 10.1021/acsmedchemlett.4c00146 (PMC11181478; doi:10.1021/acsmedchemlett.4c00146)
Supplement: Supplementary file 1 — ml4c00146_si_002.pdf [file ml4c00146_si_002.pdf]

Supporting Information for

SARS-CoV-2 Main Protease Inhibitors That Leverage Unique Interactions with the Solvent Exposed S3 Site of the Enzyme

Lauren R. Blankenship[a,‡], Kai S. Yang[a,‡], Veerabhadra R. Vulupala[a], Yugendar R. Alugubelli[a], Kaustav Khatua[a], Demonta Coleman[a], Xinyu R. Ma[a], Banumathi Sankaran[b], Chia-Chuan D. Cho[a], Yuying Ma[a], Benjamin W. Neuman[c,d,e], Shiqing Xu[a,f,*], and Wenshe Ray Liu[a,f,g,h,i,*]

[a]Texas A&M Drug Discovery Center and Department of Chemistry, College of Arts and Scienes, Texas A&M University, College Station, TX 77843, United States

[b]Molecular Biophysics and Integrated Bioimaging, Berkeley Center for Structural Biology, Laurence Berkeley National Laboratory, Berkeley, CA 94720, United States

[c]Department of Biology, College of Arts and Sciences, Texas A&M University, College Station, TX 77843, United States

[d]Texas A&M Global Health Research Complex, Texas A&M University, College Station, TX 77843, United States

[e]Department of Molecular Pathogenesis and Immunology, School of Medicine, Texas A&M University, College Station, TX 77843, United States

[f]Department of Pharmaceutical Sciences, Irma Lerma Rangel School of Pharmacy, Texas A&M University, College Station, TX 77843, United States

[g]Institute of Biosciences and Technology and Department of Translational Medical Sciences, School of Medicine, Texas A&M University, Houston, TX 77030, United States

[h]Department of Biochemistry and Biophysics, College of Agriculture and Life Sciences, Texas A&M University, College Station, TX 77843, United States

[i]Department of Cell Biology and Genetics, School of Medicine, Texas A&M University, College Station, TX 77843, United States

[‡]Contributed equally to the paper.

[*]Correspondence should be addressed to Shiqing Xu: shiqing.xu@tamu.edu and Wenshe Ray Liu: wsliu2007@tamu.edu

Table of Contents

Supplementary Methods…………………………………………………………………..S3

Supplementary Figures…………………………………………………………………...S36

Supplementary Tables…………………………………………………………………….S52

Supplementary Methods

During the synthesis of designed molecules, no unexpected or unusually high safety hazards were encountered.

The Synthesis of MPIs

All reagents and solvents for the synthesis were purchased from commercial sources and used without purification. All glassware was flame-dried prior to use. Thin-layer chromatography (TLC) was carried out on aluminum plates coated with 60 F254 silica gel. TLC plates were visualized under UV light (254 or 365 nm) or stained with 5% phosphomolybdic acid. Normal phase column chromatography was carried out using a Yamazen Small Flash AKROS system. NMR spectra were recorded on a Bruker AVANCE Neo 400 MHz spectrometer in specified deuterated solvents. Analytical HPLC-MS was performed on a PHENOMENEX C18 Column (150 × 2.00 mm 5u micron, gradient from 10% to 100% B [A = 10 mmol/L HCOONH4 /H2O, B = MeOH], flow rate: 0.3 mL/min) using a Thermo Scientific Ultimate 3000 with a UV-detector (detection at 215 nm), equipped with a Thermo Scientific Orbitrap Q Exactive Focus System. MI-14, MI-30, MI-31, MPI50, MPI51, MPI52, MPI54, MPI57, and MPI69 were synthesized according to the literature procedures.[1-3] They were analyzed by HPLC to confirm their purity as >95% before their use in soaking with apo-M^{Pro} crystals for the X-ray crystallography analysis.

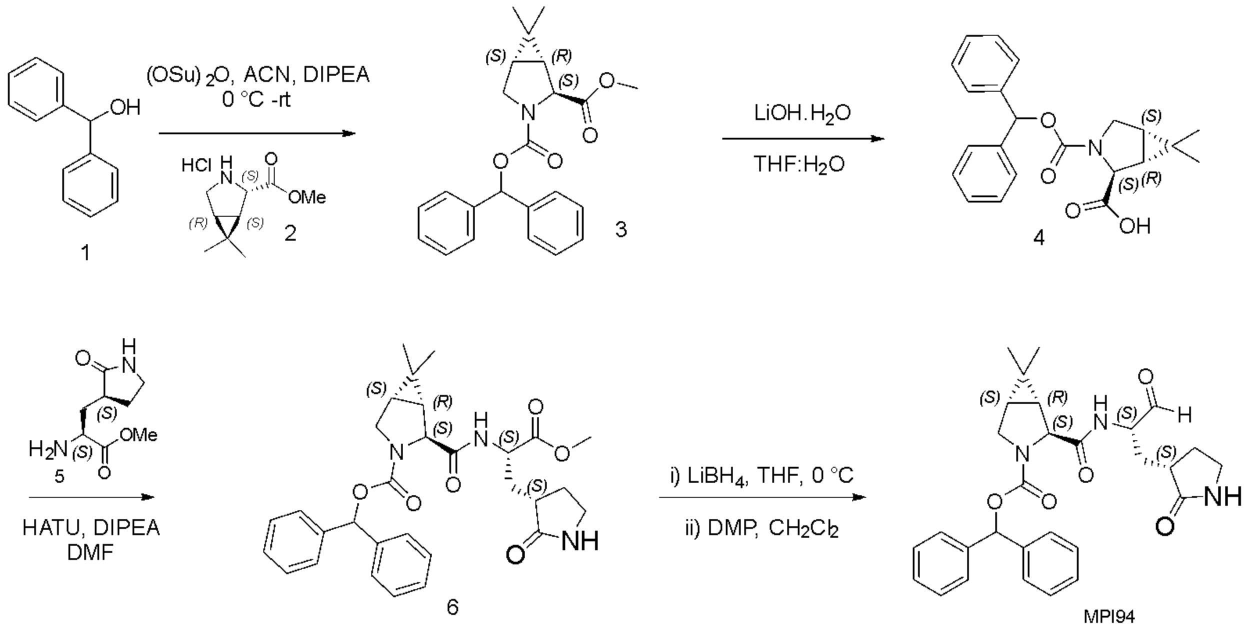

3-Benzhydryl 2-methyl (1R,2S,5S)-6,6-dimethyl-3-azabicyclo[3.1.0]hexane-2,3-dicarboxylate (3)

To a solution of **1** (200 mg, 0.637 mmol) in anhydrous ACN (5 mL) was added NMM (200 mg, 1.91 mmol) at 0 °C. bis(2,5-dioxopyrrolidin-1-yl) carbonate (228 mg, 1.2 mmol) was slowly added to the solution at the same temperature. The reaction mixture was then allowed to warm up to room temperature and stirred overnight. Then Compound 2 (298 mg, 0.637) was added to reaction and stirred for 2 h. Solvent was removed vacuum and 20 ml Ethyl acetate added to residue. The reaction mixture was then washed by 1 M HCl (2×10 mL). The organic layer was separated, dried over anhydrous Na_2SO_4, and concentrated *in vacuo*. The residue was purified by flash chromatography (50~100% EtOAc in hexanes as eluent) to yield **3** as white solid (250 mg, 49%). ^{1}H NMR (400 MHz, DMSO) δ 7.45 – 7.21 (m, 10H), 6.64 (d, *J* = 6.0 Hz, 1H), 4.35 (s, 0.55H), 4.07 (s, 0.45H), 3.78 (dd, *J* = 10.9, 5.1 Hz, 0.45H), 3.64 (s, 3H), 3.57 (dd, *J* = 11.0, 5.1 Hz, 0.55H), 1.57 – 1.42 (m, 2H), 1.03 (s, 3H), 0.89 (d, *J* = 1.8 Hz, 3H).

(1R,2S,5S)-3-((Benzhydryloxy)carbonyl)-6,6-dimethyl-3-azabicyclo[3.1.0]hexane-2-carboxylic acid (4)

The amino ester **4** (240 mg, 0.57 mmol) was dissolved in THF/H_2O (1:1, 10 mL). LiOH (73 mg, 1.77 mmol) was added at 0 °C. The mixture was stirred at room temperature overnight. Then THF was removed *on vacuum* and the aqueous layer was acidified with 1 M HCl and extracted with dichloromethane (3 x 10 mL). The organic layer was dried over anhydrous Na_2SO_4 and concentrated under reduced pressure to compound **4** as white solid. Crude material directly used for the next step without further purification.

Benzhydryl (1R,2S,5S)-2-(((S)-1-methoxy-1-oxo-3-((S)-2-oxopyrrolidin-3-yl)propan-2-yl)carbamoyl)-6,6-dimethyl-3-azabicyclo[3.1.0]hexane-3-carboxylate (6)

6 was prepared as a white solid following a similar procedure to **b168** (yield 91%). Note: this product isolated as a mixture of isomers. ^{1}H NMR (400 MHz, $CDCl_3$) δ 8.01 (d, *J* = 6.0 Hz, 0.5H), 7.38 (d, *J* = 7.4 Hz, 0.5H), 7.29 – 7.09 (m, 10H), 6.67 (d, *J* = 4.4 Hz, 1H), 6.19 (s, 0.5H), 5.99 (s, 0.5H), 4.48 (ddd, *J* = 11.1, 7.4, 4.2 Hz, 0.5H), 4.29 (ddd, *J* = 10.2, 6.0, 4.0 Hz, 0.5H), 4.22 (s, 0.5H), 4.06 (s, 0.5H), 3.79 (dd, *J* = 10.9, 5.2 Hz, 0.5H), 3.69 – 3.58 (m, 2.5H), 3.52 (d, *J* = 2.2

Hz, 2H), 3.22 – 3.01 (m, 2H), 2.38 – 1.93 (m, 3H), 1.83 – 1.60 (m, 2H), 1.49 (dd, J = 7.6, 6.3 Hz, 1H), 1.42 – 1.36 (m, 1H), 0.96 (d, J = 7.1 Hz, 3H), 0.79 (d, J = 22.5 Hz, 3H).

Benzhydryl (1R,2S,5S)-6,6-dimethyl-2-(((S)-1-oxo-3-((S)-2-oxopyrrolidin-3-yl)propan-2-yl)carbamoyl)-3-azabicyclo[3.1.0]hexane-3-carboxylate (MPI94)

MPI94 was prepared by using b55& b57 procedures.(Yield: 60%). Note: this product is isolated as a mixture of isomers.^{1}H NMR (400 MHz, $CDCl_3$) δ 9.36 (s, 0.4H), 8.91 (d, J = 1.6 Hz, 0.6H), 8.76 (d, J = 4.8 Hz, 0.6H), 7.96 (d, J = 6.3 Hz, 0.4H), 7.30 – 7.11 (m, 10H), 6.76 (s, 0.6H), 6.67 (s, 0.4H), 6.45 (s, 0.6H), 5.97 (s, 0.4H), 4.32 – 4.22 (m, 1H), 4.12 (s, 0.4H), 3.98 (dtd, J = 10.3, 5.1, 1.6 Hz, 0.6H), 3.83 (dd, J = 11.0, 5.3 Hz, 0.4H), 3.69 (dd, J = 11.4, 5.4 Hz, 0.6H), 3.63 (d, J = 10.9 Hz, 0.4H), 3.52 (d, J = 11.3 Hz, 0.6H), 3.27 – 3.08 (m, 2H), 2.39 – 2.11 (m, 2H), 1.92 – 1.76 (m, 1H), 1.75 – 1.58 (m, 2H), 1.49 (d, J = 7.5 Hz, 1H), 0.97 (d, J = 5.5 Hz, 3H), 0.81 (d, J = 11.2 Hz, 3H). ^{13}C NMR (101 MHz, $CDCl_3$) δ 200.33, 199.84, 180.20, 180.02, 173.55, 172.61, 153.83, 153.32, 140.72, 140.62, 140.48, 140.21, 128.51, 128.39, 128.36, 127.91, 127.85, 127.79, 127.66, 127.51, 126.91, 126.81, 126.69, 78.16, 77.97, 77.26, 61.62, 61.39, 58.69, 57.72, 53.45, 47.25, 46.91, 40.79, 40.54, 38.90, 37.93, 32.95, 31.35, 29.73, 29.54, 29.09, 28.61, 27.42, 26.21, 26.16, 19.32, 19.19, 12.61, 12.41. HRMS (ESI): m/z = 504.2480 $[M + H]^{+}$. HPLC purity: >99%.

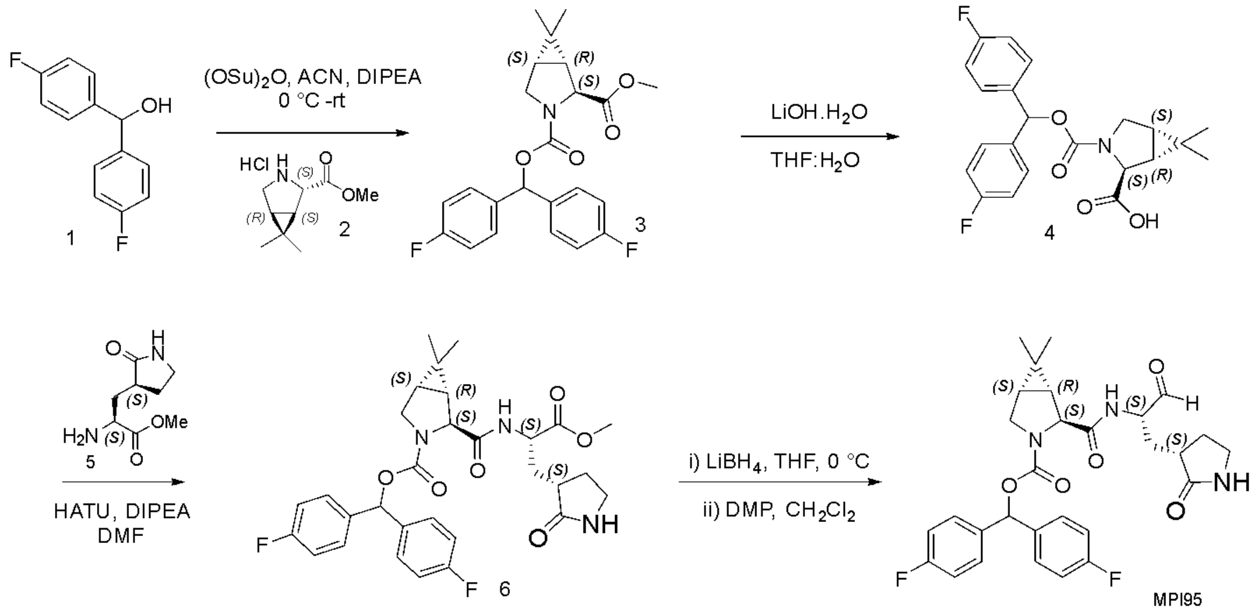

3-(Bis(4-fluorophenyl)methyl) 2-methyl (1R,2S,5S)-6,6-dimethyl-3-azabicyclo[3.1.0]hexane-2,3-dicarboxylate (2)

To a solution of 1 (250 mg, 1.35 mmol) in anhydrous ACN (5 mL) was added DIPEA (0.71 mL, 4.07 mmol) at 0 °C. bis(2,5-dioxopyrrolidin-1-yl) carbonate (228 mg, 1.9 mmol) was slowly added to the solution at the same temperature. The reaction mixture was then allowed to warm up to room temperature and stirred overnight. Then Compound 2 (278 mg, 1.35 mmol) was added to reaction and stirred for 2 h. Solvent was removed vacuum and 20 ml Ethyl acetate added to residue. The reaction mixture was then washed by 1 M HCl (2×10 mL). The organic layer was separated, dried over anhydrous Na_2SO_4, and concentrated *in vacuo*. The residue was purified by flash chromatography (50~100% EtOAc in hexanes as eluent) to yield 3 as white solid (250 mg, 51%). (45% : 55% isomers). ^{1}H NMR (400 MHz, DMSO) δ 7.50 – 7.35 (m, 3H), 7.34 – 7.26 (m, 1H), 7.25 – 7.08 (m, 4H), 6.67 (d, *J* = 5.9 Hz, 1H), 4.36 (s, 0.55H), 4.06 (s, 0.45H), 3.75 (dd, *J* = 10.9, 5.1 Hz, 0.45H), 3.63 (d, *J* = 1.4 Hz, 3H), 3.56 (dd, *J* = 11.0, 5.1 Hz, 0.55H), 1.59 – 1.42 (m, 2H), 1.02 (s, 3H), 0.88 (s, 3H).

(1R,2S,5S)-3-((Bis(4-fluorophenyl)methoxy)carbonyl)-6,6-dimethyl-3-azabicyclo[3.1.0]hexane-2-carboxylic acid (4)

The amino ester **4** (240 mg, 0.57 mmol) was dissolved in THF/H_2O (1:1, 10 mL). LiOH (73 mg, 1.77 mmol) was added at 0 °C. The mixture was stirred at room temperature overnight. Then THF was removed *on vacuum* and the aqueous layer was acidified with 1 M HCl and extracted with dichloromethane (3 x 10 mL). The organic layer was dried over anhydrous Na_2SO_4 and concentrated under reduced pressure to compound **4** as white solid. Crude material directly used for the next step without further purification.

Bis(4-fluorophenyl)methyl (1R,2S,5S)-2-(((S)-1-methoxy-1-oxo-3-((S)-2-oxopyrrolidin-3-yl)propan-2-yl)carbamoyl)-6,6-dimethyl-3-azabicyclo[3.1.0]hexane-3-carboxylate (6)

6 was prepared as a white solid following a similar procedure to **b168** (yield 91%). Note: this product isolated as a mixture of isomers. ^{1}H NMR (400 MHz, $CDCl_3$) δ 8.12 (d, *J* = 6.0 Hz, 0.55H), 7.41 (d, *J* = 7.3 Hz, 0.45H), 7.18 (dh, *J* = 10.7, 2.4 Hz, 4H), 6.97 – 6.82 (m, 4H), 6.26 (s, 0.55H), 6.12 – 5.97 (m, 0.45H), 4.46 (ddd, *J* = 11.2, 7.3, 4.1 Hz, 0.45H), 4.25 (ddd, *J* = 11.2, 5.8,

3.9 Hz, .55H), 3.76 (dd, J = 10.9, 5.3 Hz, 0.45H), 3.68 – 3.59 (m, 2H), 3.56 (s, 2H), 3.53 – 3.47 (m, 1H), 3.27 – 3.03 (m, 2H), 2.26 (dddd, J = 24.6, 12.0, 8.7, 6.0 Hz, 1H), 2.15 – 1.90 (m, 2H), 1.83 – 1.64 (m, 2H), 1.48 (dd, J = 7.5, 3.0 Hz, 1H), 1.42 – 1.24 (m, 3H), 0.95 (d, J = 7.9 Hz, 3H), 0.80 (s, 1.3H), 0.74 (s, 1.7H). ^{13}C NMR (101 MHz, $CDCl_3$) δ 179.75, 179.73, 172.66, 172.25, 171.92, 171.76, 163.56, 163.54, 163.44, 163.37, 162.60, 161.11, 161.08, 160.99, 160.92, 153.56, 153.18, 136.47, 136.44, 136.38, 136.35, 136.31, 136.28, 136.21, 136.18, 128.89, 128.82, 128.81, 128.77, 128.74, 128.69, 128.55, 128.47, 115.60, 115.58, 115.45, 115.39, 115.36, 115.23, 115.20, 114.99, 61.51, 61.29, 55.43, 52.61, 52.45, 52.33, 51.32, 47.38, 47.00, 43.40, 40.57, 40.40, 38.88, 38.62, 38.14, 36.51, 33.23, 32.85, 32.82, 31.45, 31.14, 28.80, 28.33, 27.39, 26.22, 26.16, 19.26, 19.16, 18.59, 17.23, 12.62, 12.46, 12.42.

Bis(4-fluorophenyl)methyl (1R,2S,5S)-6,6-dimethyl-2-(((S)-1-oxo-3-((S)-2-oxopyrrolidin-3-yl)propan-2-yl)carbamoyl)-3-azabicyclo[3.1.0]hexane-3-carboxylate (MPI95)

MPI95 was prepared by using b55& b57 procedures.(Yield: 60%). Note: this product isolated as a mixture of isomers. ^{1}H NMR (400 MHz, $CDCl_3$) δ 9.38 (d, J = 0.9 Hz, 0.4H), 9.05 (d, J = 1.5 Hz, .6H), 8.73 (d, J = 4.7 Hz, .6H), 8.03 (d, J = 6.1 Hz, 0.4H), 7.26 – 7.11 (m, 5H), 6.99 – 6.84 (m, 5H), 6.71 (s, 0.6H), 6.63 (s, 0.4H), 6.23 (s, 0.6H), 5.87 (s, 0.4H), 4.32 – 4.20 (m, 1H), 4.09 (d, J = 12.8 Hz, 0.4H), 4.05 – 3.96 (m, 0.6H), 3.81 (dd, J = 10.9, 5.3 Hz, 0.4H), 3.69 (dd, J = 11.3, 5.4 Hz, 0.6H), 3.54 (dd, J = 23.2, 11.2 Hz, 1H), 3.22 (dddd, J = 18.7, 16.3, 8.4, 5.7 Hz, 2H), 2.42 – 2.13 (m, 2H), 1.89 – 1.81 (m, 1H), 1.80 – 1.57 (m, 2H), 1.48 (d, J = 7.4 Hz, 1H), 1.34 (ddd, J = 37.6, 7.4, 5.2 Hz, 1H), 0.97 (d, J = 5.5 Hz, 3H), 0.80 (d, J = 14.4 Hz, 3H). ^{13}C NMR (101 MHz, CDCl3) δ 199.58, 180.08, 173.44, 172.56, 171.17, 163.56, 161.10, 153.08, 136.40, 129.25, 129.17, 128.78, 128.70, 128.64, 128.59, 128.56, 128.51, 115.64, 115.42, 115.39, 115.20, 115.17, 61.46, 60.41, 57.86, 47.31, 46.91, 40.77, 40.55, 38.91, 38.05, 32.95, 31.45, 29.75, 29.53, 29.11, 28.73, 27.36, 26.22, 26.13, 21.06, 19.33, 19.23, 14.21, 12.56, 12.38. HRMS (ESI): m/z = 540.2310 [M + H]$^{+}$. HPLC purity: >99%.

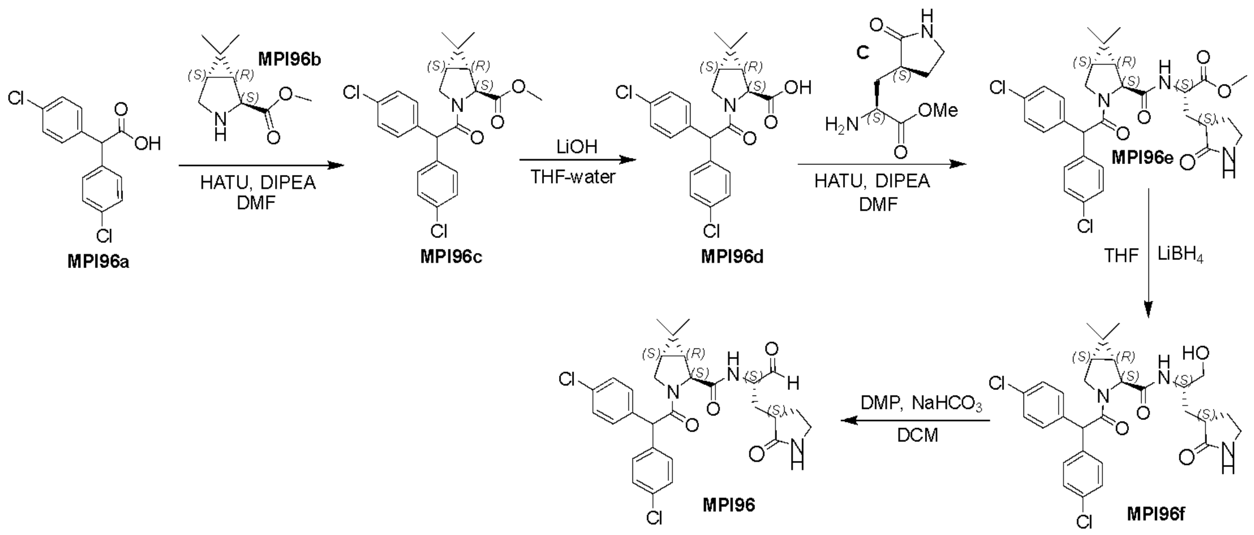

Methyl (1R,2S,5S)-3-(2,2-bis(4-chlorophenyl)acetyl)-6,6-dimethyl-3-azabicyclo[3.1.0]hexane-2-carboxylate (MPI96c).

To a solution of **MPI96a** (1.42 mmol, 400 mg) and **MPI96b** (1.56 mmol, 325 mg) in anhydrous DMF (15 mL) was added DIPEA (5.7 mmol, 737 mg, 1.1 mL) and was cooled to 0°C. HATU (1.85 mmol, 710 mg) was added to the solution under 0 °C and then stirred at room temperature overnight. The reaction mixture was then diluted with ethyl acetate (50 mL) and washed with saturated $NaHCO_3$ solution (2×20 mL), 1 M HCl solution (2×20 mL), and saturated brine solution (2×20 mL) sequentially. The organic layer was dried over anhydrous Na_2SO_4 and then concentrated *on vacuo*. The residue was then purified with flash chromatography (50-100% EtOAc in hexanes as the eluent) to afford **MPI96c** (500 mg, 75%). ^{1}H NMR (400 MHz, Chloroform-*d*) δ 7.35 – 6.82 (m, 8H), 4.88 (s, 1H), 4.35 (s, 1H), 3.64 (d, *J* = 25.8 Hz, 4H), 3.41 (d, *J* = 10.0 Hz, 1H), 1.41 – 1.30 (m, 2H), 0.93 (d, *J* = 8.7 Hz, 3H), 0.73 (s, 2H). ^{13}C NMR (100 MHz, Chloroform-*d*) δ 172.23, 171.90, 169.52, 136.83, 136.64, 133.39, 133.22, 130.43, 130.28, 130.24, 129.10, 128.81, 128.79, 128.46, 59.93, 55.02, 52.39, 47.19, 30.46, 27.48, 26.10, 19.52, 12.57.

(1R,2S,5S)-3-(2,2-Bis(4-chlorophenyl)acetyl)-6,6-dimethyl-3-azabicyclo[3.1.0]hexane-2-carboxylic acid (MPI96d).

To a solution of **MPI96c** (0.5 g, 1.06 mmol) in THF (15 mL) was added an aqueous solution of LiOH (110 mg, 2.66 mmol). The reaction mixture was stirred at room temperature for

3 h and then diluted with water (10 mL), extracted with dichloromethane (2×10 mL). The organic layers were combined and dried with anhydrous Na_2SO_4 and concentrated *in vacuo*. The crude product was used without further purification for the next step. ^{1}H NMR (400 MHz, DMSO-d_6) δ 7.49 – 7.06 (m, 8H), 4.13 (s, 1H), 4.03 (q, *J* = 7.1 Hz, 1H), 2.51 (p, *J* = 1.8 Hz, 1H), 1.60 – 1.31 (m, 2H), 1.17 (t, *J* = 7.1 Hz, 1H), 0.95 (d, *J* = 6.5 Hz, 3H), 0.70 (s, 2H), 0.55 (s, 1H). ^{13}C NMR (100 MHz, DMSO-d_6) δ 172.94, 169.16, 138.65, 138.52, 138.30, 132.22, 132.04, 131.17, 131.05, 131.00, 128.84, 128.65, 128.45, 60.25, 59.95, 53.44, 47.07, 30.27, 27.11, 26.34, 26.15, 21.22, 19.20, 14.54, 12.75.

Methyl (S)-2-((1R,2S,5S)-3-(2,2-bis(4-chlorophenyl)acetyl)-6,6-dimethyl-3-azabicyclo[3.1.0]hexane-2-carboxamido)-3-((S)-2-oxopyrrolidin-3-yl)propanoate (MPI96e).

To a solution of **MPI96d** (1mmol, 500 mg) and **C** (1.21 mmol, 270 mg) in anhydrous DMF (15 mL) was added DIPEA (4 mmol, 516 mg, 0.72 mL) and was cooled to 0°C. HATU (1.3 mmol, 494 mg) was added to the solution under 0 °C and then stirred at room temperature overnight. The reaction mixture was then diluted with ethyl acetate (50 mL) and washed with saturated $NaHCO_3$ solution (2×20 mL), 1 M HCl solution (2×20 mL), and saturated brine solution (2×20 mL) sequentially. The organic layer was dried over anhydrous Na_2SO_4 and then concentrated *on vacuo*. The residue was purified by column chromatography (MeOH: DCM = 1:10 v/v) to afford the pure product **MPI96e** (500 mg, 73%). ^{1}H NMR (400 MHz, Chloroform-*d*) δ 7.60 (d, *J* = 7.2 Hz, 1H), 7.40 – 7.06 (m, 8H), 5.97 (s, 1H), 5.02 (d, *J* = 35.4 Hz, 1H), 4.60 – 4.50 (m, 1H), 4.42 – 4.28 (m, 1H), 3.88 (dd, *J* = 10.0, 5.4 Hz, 1H), 3.80 – 3.68 (m, 3H), 3.46 (d, *J* = 10.0 Hz, 1H), 3.38 – 3.08 (m, 2H), 2.53 – 1.72 (m, 6H), 1.60 – 1.40 (m, 2H), 1.39 – 1.20 (m, 1H), 0.99 (d, *J* = 29.3 Hz, 3H), 0.75 (s, 2H), 0.48 (s, 1H).

(1R,2S,5S)-3-(2,2-Bis(4-chlorophenyl)acetyl)-N-((S)-1-hydroxy-3-((S)-2-oxopyrrolidin-3-yl)propan-2-yl)-6,6-dimethyl-3-azabicyclo[3.1.0]hexane-2-carboxamide (MPI96f).

To a solution of **MPI96e** (0.5 g, 0.8 mmol, 1.0 equiv) in anhydrous THF (10 mL) at 0 °C was added $LiBH_4$ (2.0 M in THF, 1mL, 2.0 mmol, 5.0 equiv). The mixture was stirred at RT for 2 h. After the reaction was completed, excess reactants were consumed by slow addition of H_2O. The mixture was diluted with H_2O and extracted with EtOAc, washed with sat. NaCl, dried over

Na_2SO_4 and concentrated. The residue was purified by column chromatography (MeOH: DCM = 1:12 v/v) to afford the pure product **MPI96f** as a white solid (400 mg, 84%). ^{1}H NMR (400 MHz, Chloroform-*d*) δ 7.48 (d, *J* = 7.7 Hz, 1H), 7.36 – 6.91 (m, 8H), 6.45 (s, 1H), 4.94 (s, 1H), 4.21 (s, 1H), 3.99 – 3.76 (m, 2H), 3.60 – 3.51 (m, 1H), 3.49 – 3.39 (m, 1H), 3.32 (d, *J* = 10.1 Hz, 1H), 3.23 – 2.92 (m, 2H), 2.47 – 2.36 (m, 1H), 2.30 – 2.12 (m, 1H), 2.02 – 1.85 (m, 1H), 1.74 – 1.55 (m, 1H), 1.49 – 1.30 (m, 3H), 0.88 (d, *J* = 15.3 Hz, 3H), 0.61 (s, 2H), 0.40 (s, 1H). ^{13}C NMR (100 MHz, Chloroform-*d*) δ 181.29, 180.89, 173.02, 171.90, 170.39, 170.01, 137.43, 136.04, 134.99, 133.46, 132.95, 132.78, 130.53, 130.42, 130.39, 129.19, 128.97, 128.50, 128.23, 65.03, 61.80, 54.85, 50.84, 50.43, 47.70, 40.53, 38.51, 38.08, 32.02, 31.15, 28.84, 28.40, 27.52, 26.07, 19.44, 19.24, 12.63, 12.20.

(1R,2S,5S)-3-(2,2-Bis(4-chlorophenyl)acetyl)-6,6-dimethyl-N-((S)-1-oxo-3-((S)-2-oxopyrrolidin-3-yl)propan-2-yl)-3-azabicyclo[3.1.0]hexane-2-carboxamide (MPI96).

To a solution of **MPI96f** (400 mg, 0.67 mmol, 1.0 equiv) in anhydrous DCM (10 mL) was added Dess-Martin reagent (875 mg, 2 mmol, 3.0 equiv) slowly at 0 °C. Then the reaction mixture was stirred at RT for 2 h. A solution of $NaHCO_3$ and $Na_2S_2O_3$ was added to quench the reaction. After 10 min, the mixture was washed with water, sat. NaCl, dried over Na_2SO_4 and concentrated. The residue was purified by column chromatography (MeOH: DCM = 1:10 v/v) to yield **MPI96** as a white solid (312 mg, yield 78%). ^{1}H NMR (400 MHz, DMSO-*d*$_6$) δ 9.39 (s, 0H), 7.85 – 7.63 (m, 1H), 7.46 – 6.84 (m, 9H), 6.11 (d, *J* = 7.3 Hz, 0H), 5.25 (d, *J* = 2.3 Hz, 1H), 4.39 – 4.13 (m, 2H), 3.74 (dd, *J* = 10.3, 5.3 Hz, 1H), 3.16 (d, *J* = 4.1 Hz, 2H), 3.12 – 2.62 (m, 2H), 2.38 – 2.16 (m, 1H), 2.00 – 1.65 (m, 2H), 1.65 – 1.48 (m, 1H), 1.48 – 1.06 (m, 3H), 0.98 – 0.78 (m, 3H), 0.61 (d, *J* = 4.5 Hz, 2H), 0.44 (d, *J* = 15.5 Hz, 1H). ^{13}C NMR (100 MHz, DMSO-*d*$_6$) δ 201.58, 179.81, 179.00, 172.07, 171.84, 171.40, 169.19, 169.02, 139.08, 139.00, 138.83, 138.22, 138.01, 132.80, 132.28, 132.21, 131.98, 131.92, 131.89, 131.85, 131.26, 131.21, 131.12, 131.06, 130.88, 130.71, 129.27, 128.93, 128.85, 128.49, 128.44, 128.34, 56.70, 55.31, 53.50, 53.40, 38.21, 37.66, 31.30, 28.35, 27.33, 26.34, 26.22, 25.49, 19.10, 18.94, 12.95, 12.86, 12.51. HRMS (ESI) m/z = 556.1762 $[M + H]^+$. HPLC purity: 98.3%.

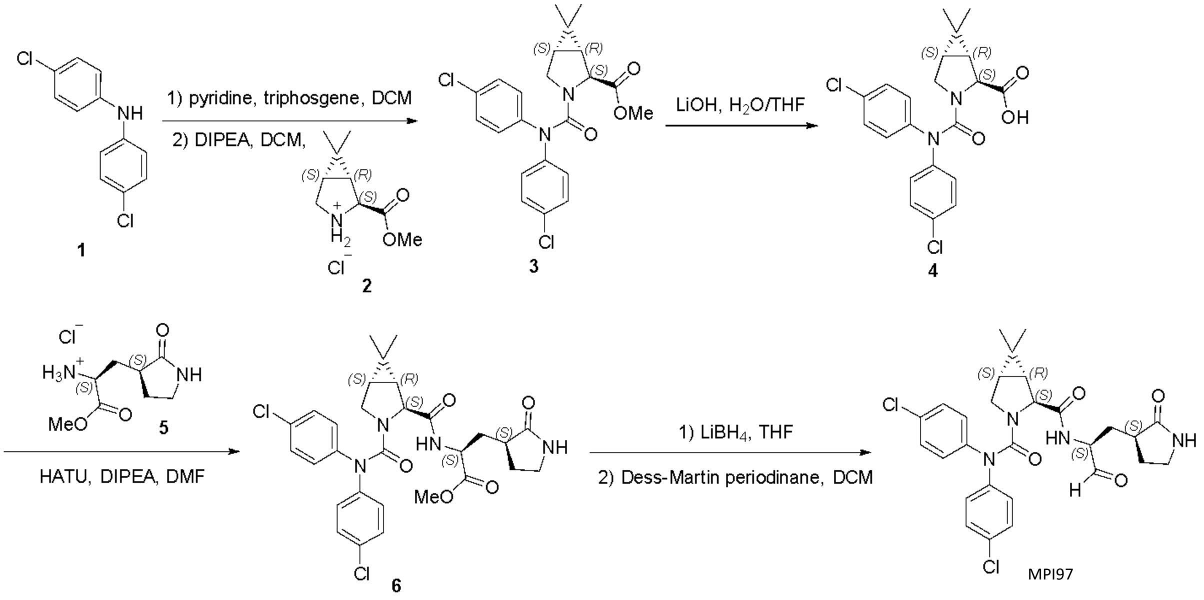

Methyl(1R,2S,5S)-3-(bis(4-chlorophenyl)carbamoyl)-6,6-dimethyl-3-azabicyclo[3.1.0]hexane-2-carboxylate (3)

To a stirred solution of triphosgene (0.5 mmol, 150 mg) in anhydrous DCM (5 mL) was added pyridine (6 mmol, 480 mg, 0.5 mL) at 0 °C. Then a solution of **1** (1 mmol, 238 mg) in anhydrous DCM (5 mL) was added dropwise at the same temperature. The resulting reaction mixture was stirred at room temperature overnight. Then it was washed with 1 M HCl solution (2×10 mL) and the organic layer was separated, dried over anhydrous Na_2SO_4 and concentrated *in vacuo*. The residue was then dissolved in anhydrous DCM (5 mL), then a solution of **2** (1 mmol, 206 mg) and DIPEA (2 mmol, 0.3 mL) in anhydrous DCM (5 mL) was added. The reaction mixture was stirred at room temperature overnight. Then the reaction mixture was washed with 1 M HCl solution (2×10 mL). The organic layer was separated, dried over anhydrous Na_2SO_4 and concentrated *in vacuo*. The residue was purified by flash chromatography (10~50% EtOAc in hexanes as eluent) to yield compound **3** as white solid (230 mg, 53%).^{1}H NMR (400 MHz, Chloroform-*d*) δ 7.28 (d, *J* = 8.5 Hz, 4H), 7.01 (d, *J* = 7.5 Hz, 4H), 4.71 (s, 1H), 3.78 (s, 3H), 3.38 – 2.92 (m, 2H), 1.43 (d, *J* = 7.4 Hz, 1H), 1.35 – 1.22 (m, 1H), 1.12 – 0.81 (m, 6H).

(1R,2S,5S)-3-(Bis(4-chlorophenyl)carbamoyl)-6,6-dimethyl-3-azabicyclo[3.1.0]hexane-2-carboxylic acid (4)

To a stirred solution of **3** (0.53 mmol, 230 mg) in THF (5 mL) was added 5 mL aqueous solution of LiOH (0.6 mmol, 24 mg). The solution was stirred at room temperature for 3 h before evaporation *in vacuo*. The residue was acidified with 1 M HCl and extracted with DCM (2×20 mL). The organic layers were combined, dried over anhydrous Na_2SO_4 and concentrated *in vacuo* to yield **4** as white solid (210 mg, 95%). The compound was used without further purification.

Methyl(S)-2-((1R,2S,5S)-3-(bis(4-chlorophenyl)carbamoyl)-6,6-dimethyl-3-azabicyclo[3.1.0]hexane-2-carboxamido)-3-((S)-2-oxopyrrolidin-3-yl)propanoate (6)

To a stirred solution of **4** (0.5 mmol, 210 mg) and **5** (0.5 mmol, 111 mg) in anhydrous DMF (5 mL) was added DIPEA (1.5 mmol, 250 uL). The solution was cooled at 0 °C, then HATU (0.6 mmol, 228 mg) was added at the same temperature. The reaction mixture was then stirred at room temperature overnight, diluted with EtOAc (50 mL), and washed with saturated $NaHCO_3$ solution (2×20 mL), 1 M HCl (2×20 mL) and saturated brine solution (2×20 mL). The organic layer was then dried over anhydrous Na_2SO_4, filtered and concentrated *in vacuo*. The residue was then purified by flash chromatography (1~10% methanol in DCM as eluent) to yield **6** as white solid (190 mg, 65%).^{1}H NMR (400 MHz, Chloroform-*d*) δ 7.49 (d, J = 7.2 Hz, 1H), 7.31 (d, J = 8.8 Hz, 4H), 7.02 (d, J = 8.8 Hz, 4H), 6.05 (s, 1H), 4.59 (s, 2H), 3.76 (s, 3H), 3.39 – 3.22 (m, 2H), 3.22 – 3.04 (m, 1H), 2.53 – 2.37 (m, 2H), 2.26 – 2.11 (m, 1H), 2.00 – 1.71 (m, 3H), 1.57 – 1.48 (m, 1H), 1.39 – 1.31 (m, 1H), 1.10 – 0.92 (m, 6H). ^{13}C NMR (101 MHz, $CDCl_3$) δ 179.6, 172.3, 172.2, 157.6, 142.4, 130.7, 129.5, 126.9, 53.4, 52.5, 49.4, 40.4, 38.6, 38.3, 33.4, 28.6, 26.1, 19.3, 12.9.

(1R,2S,5S)-N3,N3-Bis(4-chlorophenyl)-6,6-dimethyl-N2-((S)-1-oxo-3-((S)-2-oxopyrrolidin-3-yl)propan-2-yl)-3-azabicyclo[3.1.0]hexane-2,3-dicarboxamide (MPI97)

To a solution of **6** (0.17 mmol, 100 mg) in anhydrous THF (5 mL) was dropwise added a 2 M solution of $LiBH_4$ (0.34 mmol, 0.17 mL) at 0 °C. The solution was stirred at the same temperature for 3 h. Then the reaction was quenched with slow addition of saturated NH_4Cl solution. The organic layer was separated, dried over anhydrous Na_2SO_4, and concentrated *in vacuo*. The residue was then purified by flash chromatography (1~10% methanol in DCM as eluent). The purified product was then dissolved in anhydrous DCM (5 mL). Dess-Martin periodinane (0.2 mmol, 84 mg) was added to the solution and the reaction mixture was then stirred at room temperature for 3

h. Then the reaction was quenched by 10% $Na_2S_2O_3$ in saturated $NaHCO_3$ solution (5 mL). The mixture was further stirred for 15 min until the organic layer turned clear. Then organic layer was separated, dried over anhydrous Na_2SO_4, and concentrated *in vacuo*. The residue was then purified by flash chromatography (1~10% methanol in DCM as eluent) to yield **MPI97** as white solid (52 mg, 55%).^{1}H NMR (400 MHz, Chloroform-*d*) δ 9.50 (s, 1H), 7.89 (s, 1H), 7.20 (d, J = 8.7 Hz, 4H), 6.91 (d, J = 8.6 Hz, 4H), 6.12 (s, 1H), 4.51 (s, 1H), 4.36 (s, 1H), 3.21 (dtd, J = 18.6, 9.3, 6.7 Hz, 2H), 3.08 (s, 1H), 2.55 – 2.18 (m, 3H), 1.91 (h, J = 8.5, 7.8 Hz, 2H), 1.76 (dq, J = 12.3, 9.1 Hz, 1H), 1.39 (d, J = 7.6 Hz, 1H), 1.26 (t, J = 6.5 Hz, 1H), 0.94 (s, 3H), 0.88 (s, 3H). ^{13}C NMR (101 MHz, $CDCl_3$) δ 199.3, 180.0, 172.9, 157.5, 142.3, 130.8, 129.5, 126.8, 61.9, 57.8, 50.8, 49.3, 40.6, 38.0, 29.9, 28.7, 26.1, 19.4, 13.0. HRMS (ESI): m/z = 558.1633 $[M + H]^+$. HPLC purity: >99%.

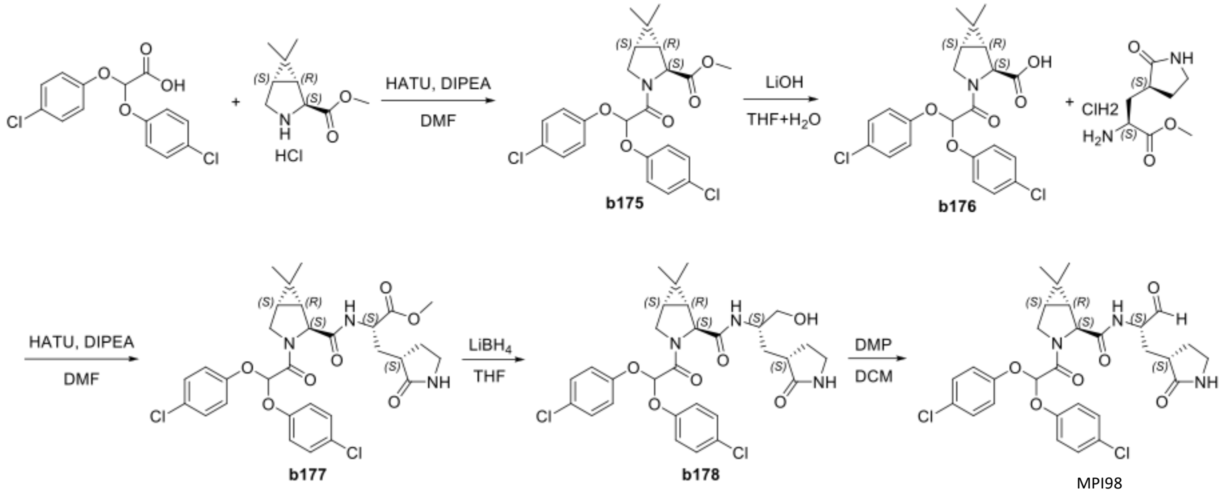

Methyl (1R,2S,5S)-3-(2,2-bis(4-chlorophenoxy)acetyl)-6,6-dimethyl-3-azabicyclo[3.1.0]hexane-2-carboxylate (b175)

b175 was prepared as a white solid following a similar procedure to **b50** (yield 78%). Note: this compound was actually obtained as a mixture of isomers. ^{1}H NMR (400 MHz, Methanol-*d*$_4$) δ 7.2 – 7.1 (m, 4H), 7.0 – 6.8 (m, 4H), 6.0 (d, J = 66.1 Hz, 1H), 4.8 (d, J = 65.7 Hz, 1H), 4.0 – 3.6 (m, 2H), 3.5 (d, J = 37.0 Hz, 3H), 1.5 – 1.4 (m, 1H), 1.4 – 1.3 (m, 1H), 0.9 (d, J = 1.5 Hz, 3H), 0.8 (d, J = 5.7 Hz, 3H). ^{13}C NMR (100 MHz, Methanol-*d*$_4$) δ 172.7, 165.5, 155.3, 155.2, 130.8, 130.7, 130.7, 130.6, 129.5, 129.4, 119.7, 119.6, 119.4, 119.4, 98.2, 61.7, 53.0, 47.7, 30.7, 28.8, 26.4, 20.4, 12.9.

(1R,2S,5S)-3-(2,2-bis(4-chlorophenoxy)acetyl)-6,6-dimethyl-3-azabicyclo[3.1.0]hexane-2-carboxylic acid (b176)

b176 was prepared as a white solid following a similar procedure to **b51** (yield 93%), the residue was used in the next step without further purification. Note: this compound was actually obtained as a mixture of isomers.

Methyl (S)-2-((1R,2S,5S)-3-(2,2-bis(4-chlorophenoxy)acetyl)-6,6-dimethyl-3-azabicyclo[3.1.0]hexane-2-carboxamido)-3-((S)-2-oxopyrrolidin-3-yl)propanoate (b177)

b177 was prepared as a white solid following a similar procedure to **b52** (yield 99%). Note: this compound was actually obtained as a mixture of isomers.

(1R,2S,5S)-3-(2,2-bis(4-chlorophenoxy)acetyl)-N-((S)-1-hydroxy-3-((S)-2-oxopyrrolidin-3-yl)propan-2-yl)-6,6-dimethyl-3-azabicyclo[3.1.0]hexane-2-carboxamide (b178)

b178 was prepared as a white solid following a similar procedure to **b55** (yield 70%). Note: this compound was actually obtained as a mixture of isomers. ^{1}H NMR (400 MHz, Methanol-d_4) δ 7.4 – 7.2 (m, 4H), 7.1 – 7.0 (m, 4H), 6.0 (d, *J* = 166.2 Hz, 1H), 4.4 (s, 1H), 4.2 (dd, *J* = 10.8, 5.4 Hz, 1H), 4.1 – 3.9 (m, 1H), 3.9 – 3.8 (m, 1H), 3.5 (dd, *J* = 18.2, 5.6 Hz, 2H), 3.2 (td, *J* = 9.3, 2.0 Hz, 1H), 3.1 – 2.8 (m, 1H), 2.6 (dddd, *J* = 11.7, 9.8, 8.3, 3.6 Hz, 1H), 2.2 (dddd, *J* = 12.6, 8.8, 7.1, 2.1 Hz, 1H), 2.1 – 1.9 (m, 1H), 1.8 – 1.6 (m, 2H), 1.6 – 1.4 (m, 2H), 1.1 (d, *J* = 5.4 Hz, 3H), 0.9 (d, *J* = 2.3 Hz, 3H). ^{13}C NMR (100 MHz, Methanol-d_4) δ 182.7, 173.4, 165.0, 155.5, 155.2, 130.9, 130.7, 130.6, 130.5, 129.4, 129.1, 120.0, 119.9, 119.6, 119.6, 97.4, 65.6, 63.1, 50.5, 48.1, 41.3, 39.3, 33.4, 31.9, 29.2, 29.0, 26.4, 20.3, 13.1.

(1R,2S,5S)-3-(2,2-bis(4-chlorophenoxy)acetyl)-6,6-dimethyl-N-((S)-1-oxo-3-((S)-2-oxopyrrolidin-3-yl)propan-2-yl)-3-azabicyclo[3.1.0]hexane-2-carboxamide (MPI98)

MPI98 was prepared as a white solid following a similar procedure to **b57** (yield 66%). Note: this compound was actually obtained as a mixture of isomers. ^{1}H NMR (400 MHz, Chloroform-*d*) δ 9.5 – 9.1 (m, 1H), 7.2 – 7.1 (m, 4H), 7.0 – 6.8 (m, 4H), 6.6 (d, *J* = 12.5 Hz, 1H), 5.9 (d, *J* = 10.5 Hz, 1H), 5.1 (d, *J* = 144.6 Hz, 1H), 4.4 (dt, *J* = 14.9, 5.3 Hz, 1H), 4.1 – 4.0 (m,

1H), 4.0 – 3.9 (m, 1H), 3.8 (d, J = 4.1 Hz, 1H), 3.4 – 3.1 (m, 3H), 2.5 – 2.4 (m, 1H), 2.3 – 2.2 (m, 1H), 1.9 – 1.8 (m, 1H), 1.8 – 1.7 (m, 1H), 1.6 – 1.3 (m, 2H), 1.1 – 1.0 (m, 3H), 0.9 – 0.7 (m, 3H). ^{13}C NMR (100 MHz, Chloroform-*d*) δ 199.6, 180.3, 171.7, 163.7, 154.1, 153.9, 129.7, 129.7, 129.7, 129.6, 128.5, 128.4, 119.0, 118.5, 118.4, 118.3, 98.0, 61.8, 57.7, 47.0, 40.6, 38.1, 30.3, 29.7, 28.5, 27.9, 26.2, 19.4, 12.7. HRMS (ESI): *m/z* = 589.1534 [M + H]$^+$. HPLC purity: 95.8%.

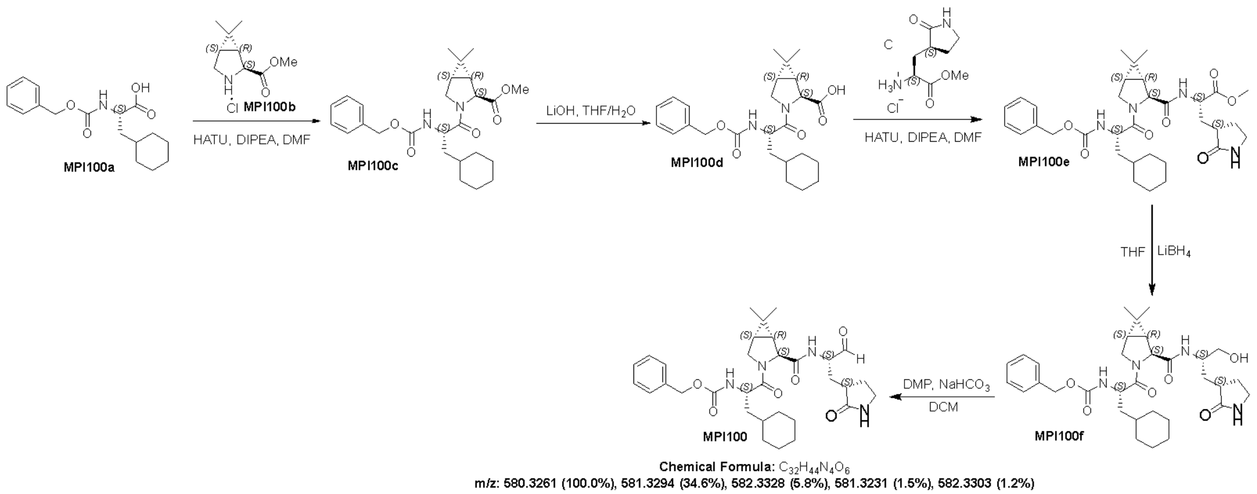

Methyl(1R,2S,5S)-3-((S)-2-(((benzyloxy)carbonyl)amino)-3-cyclohexylpropanoyl)-6,6-dimethyl-3-azabicyclo[3.1.0]hexane-2-carboxylate (MPI100c)

To a solution of **MPI100a** (1.3 mmol, 400 mg) and **MPI100b** (1.44 mmol, 300 mg) in anhydrous DMF (15 mL) was added DIPEA (5.24 mmol, 680 mg, 1.0 mL) and was cooled to 0°C. HATU (1.7 mmol, 650 mg) was added to the solution under 0 °C and then stirred at room temperature overnight. The reaction mixture was then diluted with ethyl acetate (50 mL) and washed with saturated $NaHCO_3$ solution (2×20 mL), 1 M HCl solution (2×20 mL), and saturated brine solution (2×20 mL) sequentially. The organic layer was dried over anhydrous Na_2SO_4 and then concentrated *on vacuo*. The residue was then purified with flash chromatography (50-100% EtOAc in hexanes as the eluent) to afford **MPI100c** (300 mg, 50%). ^{1}H NMR (400 MHz, CDCl3) δ 7.41 – 7.27 (m, 5H), 5.35 (d, J = 8.9 Hz, 1H), 5.18 – 5.01 (m, 2H), 4.55 – 4.46 (m, 1H), 3.87 – 3.79 (m, 2H), 3.76 (s, 3H), 1.90 (d, J = 7.0 Hz, 2H), 1.78 – 1.62 (m, 4H), 1.62 – 1.53 (m, 1H), 1.54 – 1.41 (m, 4H), 1.32 – 1.12 (m, 4H), 1.07 (d, J = 4.6 Hz, 3H), 0.97 (d, J = 3.7 Hz, 3H). ^{13}C NMR (100 MHz, $CDCl_3$) δ 171.86, 171.44, 156.19, 136.38, 128.49, 128.45, 128.07, 127.99, 127.94, 127.91, 77.39, 77.28, 77.08, 76.76, 66.84, 59.43, 52.37, 50.36, 46.77, 40.10, 33.98, 33.77, 32.60, 32.36, 30.38, 27.49, 26.44, 26.24, 26.06, 19.64, 12.84, 12.64.

(1R,2S,5S)-3-((S)-2-(((benzyloxy)carbonyl)amino)-3-cyclohexylpropanoyl)-6,6-dimethyl-3-azabicyclo[3.1.0]hexane-2-carboxylic acid (MPI100d)

To a solution of **MPI100c** (0.6 g, 1.3 mmol) in THF (15 mL) was added an aqueous solution of LiOH (135 mg, 3.3 mmol). The reaction mixture was stirred at room temperature for 3 h and then diluted with water (10 mL), extracted with dichloromethane (2×10 mL). The organic layers were combined and dried with anhydrous Na_2SO_4 and concentrated *in vacuo*. The crude product was used without further purification for the next step. ^1H NMR (400 MHz, $CDCl_3$) δ 7.39 – 7.30 (m, 5H), 5.54 (d, *J* = 8.9 Hz, 1H), 5.15 – 5.06 (m, 2H), 4.59 – 4.48 (m, 1H), 3.89 (d, *J* = 10.2 Hz, 1H), 3.80 (dd, *J* = 10.2, 5.3 Hz, 1H), 1.95 (s, 1H), 1.74 – 1.58 (m, 5H), 1.58 – 1.38 (m, 4H), 1.35 – 1.12 (m, 4H), 1.07 (s, 3H), 0.99 – 0.93 (m, 3H). ^{13}C NMR (100 MHz, $CDCl_3$) δ 174.43, 172.54, 156.26, 136.36, 128.50, 128.09, 127.92, 66.90, 59.69, 50.45, 47.07, 39.75, 33.91, 33.72, 32.59, 29.99, 27.36, 26.42, 26.21, 26.03, 19.58, 12.64.

Methyl(S)-2-((1R,2S,5S)-3-((S)-2-(((benzyloxy)carbonyl)amino)-3-cyclohexylpropanoyl)-6,6-dimethyl-3-azabicyclo[3.1.0]hexane-2-carboxamido)-3-((S)-2-oxopyrrolidin-3-yl)propanoate (MPI100e).

To a solution of **MPI100d** (0.68 mmol, 300 mg) and **C** (0.75 mmol, 170 mg) in anhydrous DMF (15 mL) was added DIPEA (2.7 mmol, 350 mg, 0.5 mL) and was cooled to 0°C. HATU (0.8 mmol, 340 mg) was added to the solution under 0 °C and then stirred at room temperature overnight. The reaction mixture was then diluted with ethyl acetate (50 mL) and washed with saturated $NaHCO_3$ solution (2×20 mL), 1 M HCl solution (2×20 mL), and saturated brine solution (2×20 mL) sequentially. The organic layer was dried over anhydrous Na_2SO_4 and then concentrated *on vacuo*. The residue was purified by column chromatography (MeOH: DCM = 1:10 v/v) to afford the pure product **MPI100e** (300 mg, 72%). ^1H NMR (400 MHz, $CDCl_3$) δ 7.94 (s, 1H), 7.57 (d, *J* = 7.3 Hz, 1H), 7.32 – 7.20 (m, 5H), 6.11 (s, 1H), 5.07 – 4.86 (m, 2H), 4.53 – 4.31 (m, 2H), 4.25 (s, 1H), 3.80 – 3.68 (m, 2H), 3.64 (s, 3H), 3.22 (dd, *J* = 9.2, 4.8 Hz, 2H), 2.50 – 2.35 (m, 1H), 2.35 – 2.23 (m, 1H), 2.12 – 2.03 (m, 1H), 1.91 – 1.67 (m, 3H), 1.66 – 1.51 (m, 4H), 1.48 (s, 2H), 1.42 – 1.25 (m, 3H), 1.22 – 1.04 (m, 3H), 0.98 (d, *J* = 4.7 Hz, 4H), 0.86 (d, *J* = 3.2 Hz, 3H). ^{13}C NMR (100 MHz, $CDCl_3$) δ 179.95, 172.23, 171.36, 162.60, 156.23, 136.40,

128.48, 128.44, 128.06, 127.97, 77.37, 77.05, 76.74, 66.82, 60.85, 52.45, 51.31, 40.52, 38.63, 38.20, 36.51, 34.05, 33.03, 31.46, 30.62, 28.45, 26.41, 26.24, 19.44, 12.82.

Benzyl((S)-3-cyclohexyl-1-((1R,2S,5S)-2-(((S)-1-hydroxy-3-((S)-2-oxopyrrolidin-3-yl)propan-2-yl)carbamoyl)-6,6-dimethyl-3-azabicyclo[3.1.0]hexan-3-yl)-1-oxopropan-2-yl)carbamate (MPI100f)

To a solution of **MPI100e** (0.3 g, 0.5 mmol, 1.0 equiv) in anhydrous THF (10 mL) at 0 °C was added $LiBH_4$ (2.0 M in THF, 0.6 mL, 1.2.0 mmol, 2.5 equiv). The mixture was stirred at RT for 2 h. After the reaction was completed, excess reactants were consumed by slow addition of H_2O. The mixture was diluted with H_2O and extracted with EtOAc, washed with sat. NaCl, dried over Na_2SO_4 and concentrated. The residue was purified by column chromatography (MeOH: DCM = 1:12 v/v) to afford the pure product **MPI100f** as a white solid (200 mg, 70%). ^{1}H NMR (400 MHz, $CDCl_3$) δ 7.53 (d, J = 7.9 Hz, 1H), 7.31 – 7.21 (m, 5H), 6.46 – 6.19 (m, 1H), 5.67 (t, J = 9.7 Hz, 1H), 5.10 – 4.89 (m, 2H), 4.39 (td, J = 9.6, 3.6 Hz, 1H), 4.24 (s, 1H), 4.11 – 3.99 (m, 1H), 3.99 – 3.86 (m, 1H), 3.86 – 3.54 (m, 4H), 3.54 – 3.37 (m, 1H), 3.29 – 3.09 (m, 2H), 2.48 – 2.20 (m, 3H), 2.06 – 1.94 (m, 1H), 1.86 – 1.75 (m, 1H), 1.75 – 1.24 (m, 12H), 1.24 – 1.00 (m, 4H), 0.97 (d, J = 2.8 Hz, 3H), 0.83 (d, J = 15.9 Hz, 4H). ^{13}C NMR (100 MHz, $CDCl_3$) δ 181.20, 171.96, 171.75, 171.65, 156.28, 136.41, 136.03, 128.52, 128.49, 128.08, 128.04, 127.97, 77.39, 77.27, 77.07, 76.75, 66.79, 65.37, 61.30, 50.64, 47.33, 40.56, 39.74, 38.14, 34.02, 33.75, 32.26, 30.89, 28.61, 27.79, 26.42, 26.22, 25.99, 19.71, 19.33, 13.10, 12.84.

Benzyl((S)-3-cyclohexyl-1-((1R,2S,5S)-6,6-dimethyl-2-(((S)-1-oxo-3-((S)-2-oxopyrrolidin-3-yl)propan-2-yl)carbamoyl)-3-azabicyclo[3.1.0]hexan-3-yl)-1-oxopropan-2-yl)carbamate (MPI100)

To a solution of **MPI100f** (185 mg, 0.32 mmol, 1.0 equiv) in anhydrous DCM (10 mL) was added Dess-Martin reagent (420 mg, 0.95 mmol, 3.0 equiv) slowly at 0 °C. Then the reaction mixture was stirred at RT for 2 h. A solution of $NaHCO_3$ and $Na_2S_2O_3$ was added to quench the reaction. After 10 min, the mixture was washed with water, sat. NaCl, dried over Na_2SO_4 and concentrated. The residue was purified by column chromatography (MeOH: DCM = 1:10 v/v) to yield **MPI100** as a white solid (150 mg, yield 81%). ^{1}H NMR (400 MHz, $CDCl_3$) δ 9.44 (d, J =

0.7 Hz, 1H), 8.06 (d, *J* = 6.2 Hz, 1H), 7.26 (q, *J* = 5.2 Hz, 5H), 5.81 – 5.61 (m, 1H), 5.08 – 4.91 (m, 2H), 4.47 – 4.39 (m, 1H), 4.39 – 4.18 (m, 2H), 3.86 – 3.61 (m, 2H), 3.33 – 3.17 (m, 2H), 2.55 – 2.40 (m, 1H), 2.40 – 2.26 (m, 1H), 1.98 – 1.74 (m, 4H), 1.69 – 1.50 (m, 8H), 1.40 – 1.27 (m, 2H), 1.23 – 1.02 (m, 4H), 0.99 (d, *J* = 3.3 Hz, 3H), 0.91 – 0.81 (m, 4H). ^{13}C NMR (100 MHz, $CDCl_3$) δ 200.66, 199.73, 180.07, 172.14, 171.75, 162.57, 156.20, 136.37, 128.50, 128.45, 128.10, 127.98, 127.84, 77.36, 77.25, 77.04, 76.73, 66.86, 60.96, 57.65, 50.55, 47.27, 40.56, 39.94, 37.88, 36.50, 34.04, 33.80, 32.36, 31.45, 30.95, 29.75, 28.70, 27.79, 26.42, 26.23, 26.01, 19.50, 12.95, 12.78. HRMS (ESI) m/z = 581.3325 $[M + H]^+$. HPLC purity: 98.3%.

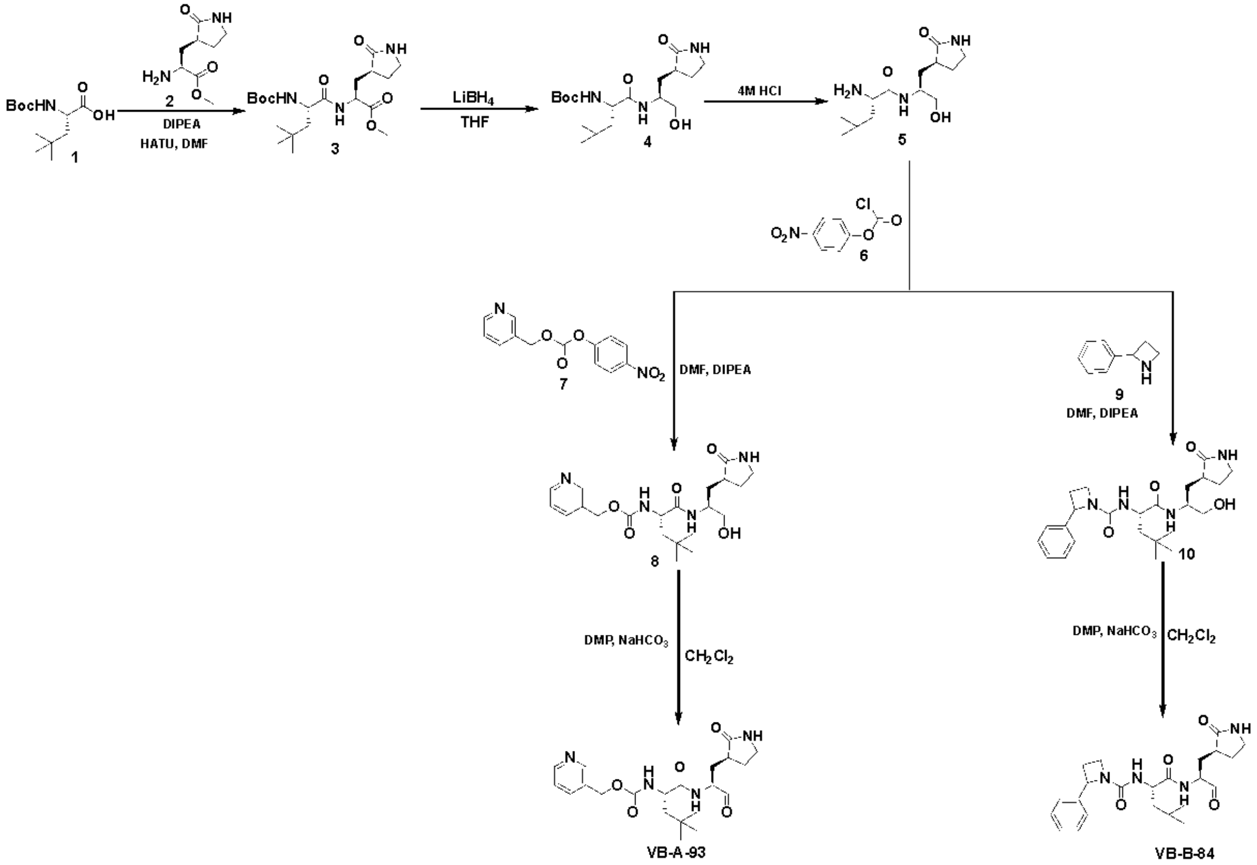

MPI101 **MPI105**

Methyl (*S*)-2-((*S*)-2-((*tert*-butoxycarbonyl)amino)-4,4-dimethylpentanamido)-3-((*S*)-2-oxopyrrolidin-3-yl)propanoate (3)

To a solution of **1** (2.04 mmol, 0.5 g) and **2** (2.04 mmol, 0.45 g) in anhydrous DMF (20 mL) was added DIPEA (6.12 mmol, 1.03 mL) and was cooled to 0 °C. HATU (3.06 mmol, 1.16

g) was added to the solution under 0 °C and then stirred at room temperature overnight. The reaction mixture was then diluted with ethyl acetate (50 mL) and washed with saturated $NaHCO_3$ solution (2×20 mL), 1 M HCl solution (2×20 mL), and saturated brine solution (2×20 mL) sequentially. The organic layer was dried over anhydrous Na_2SO_4 and then concentrated *on vacuo*. The residue was then purified by Column chromatographic purification of the residue (4% MeOH in CH_2Cl_2 as the eluent) afforded **3** as white solid (540 mg, 65%). ^{1}H NMR (400 MHz, $CDCl_3$) δ 7.50 – 7.37 (m, 1H), 5.84 (d, *J* = 37.8 Hz, 1H), 4.89 (d, *J* = 8.1 Hz, 1H), 4.59 – 4.44 (m, 1H), 4.19 (s, 1H), 3.73 (s, 3H), 3.33 (dd, *J* = 9.4, 6.6 Hz, 2H), 2.50 – 2.35 (m, 2H), 2.28 – 2.15 (m, 1H), 1.88 (d, *J* = 3.8 Hz, 1H), 1.85 (d, *J* = 3.5 Hz, 1H), 1.43 (s, 9H), 0.98 (s, 9H).

***tert*-Butyl ((*S*)-1-(((*S*)-1-hydroxy-3-((*S*)-2-oxopyrrolidin-3-yl)propan-2-yl)amino)-4,4-dimethyl-1-oxopentan-2-yl)carbamate (4)**

To a stirred solution of compound **3** (500 mg, 1.21 mmol) in THF (10 mL) was added $LiBH_4$ (2.0 M in THF, 1.51 mL, 3.02 mmol) in several portions at 0 °C under a nitrogen atmosphere. The reaction mixture was stirred at 0 °C for 1 h, then allowed to warm up to room temperature, and stirred for an additional 2 h. The reaction was quenched by the drop wise addition of 1.0 M HCl (aq) (1.2 mL) with cooling in an ice bath. The solution was diluted with ethyl acetate and H_2O. The phases were separated, and the aqueous layer was extracted with ethyl acetate (3×15 mL). The organic phases were combined, dried over $MgSO_4$, filtered, and concentrated on a rotorvap to give a yellow oily residue. Column chromatographic purification of the residue (6% MeOH in CH_2Cl_2 as the eluent) afforded 7 as white solid (350 mg, 75%). ^{1}H NMR (400 MHz, DMSO) δ 7.55 – 7.39 (m, 2H), 6.88 (d, *J* = 8.5 Hz, 1H), 4.66 (t, *J* = 5.5 Hz, 1H), 3.93 (m, 1H), 3.81 – 3.68 (m, 1H), 3.36 (dd, *J* = 10.3, 5.0 Hz, 1H), 3.28 – 2.95 (m, 3H), 2.30 – 2.10 (m, 2H), 1.81 (m, 1H), 1.59 – 1.30 (m, 13H), 0.89 (s, 9H).

(*S*)-2-Amino-*N*-((*S*)-1-hydroxy-3-((*S*)-2-oxopyrrolidin-3-yl)propan-2-yl)-4,4-dimethylpentanamide Hydrochloride salt (5)

To a stirred solution of **4** (300 mg, 0.78 mmol) in 1,4-Dioxane (1 mL) at 0 °C was added 4N HCl (1.3 mL). Reaction mixture was stirred at rt for 3 h. After completion of reaction, solvent was concentrated in a vacuum. The residue was used in the next step without further

purification(320 mg). ^{1}H NMR (400 MHz, DMSO) δ 8.61 – 8.50 (m, 1H), 8.34 (brs, 3H), 7.55 (s, 1H), 3.84 – 3.69 (m, 2H), 3.41 (dd, *J* = 10.6, 4.6 Hz, 1H), 3.26 (m, 1H), 3.13 (m, 2H), 2.22 (m, 1H), 1.92 – 1.74 (m, 2H), 1.62 – 1.35 (m, 4H), 0.92 (s, 10H).

Pyridin-3-ylmethyl ((*S*)-1-(((*S*)-1-hydroxy-3-((*S*)-2-oxopyrrolidin-3-yl)propan-2-yl)amino)-4,4-dimethyl-1-oxopentan-2-yl)carbamate (8)

To **7** (20 mg, 0.183 mmol) in DMF (5 mL) were added DIPEA (19.3 mg, 0.183 mmol) and **6** (36.6 mg, 0.183 mmol) and the mixture was stirred at room temperature for 1 h. After 1h compound **5** (0.065 mg, 0.183 mmol) in DMF (5 mL) and DIPEA (0.46 mL, 0.275mmol) were added to drop wise. The reaction mixture stirred for 12 h. The mixture was then poured into water (30 mL) and extracted with ethyl acetate (4×20 mL). The organic layer was washed with aqueous hydrochloric acid 10% v/v (2×20 mL), saturated aqueous $NaHCO_3$ (2×20 mL), brine (2×20 mL) and dried over Na_2SO_4. The organic phase was evaporated to dryness and the crude material purified by silica gel column chromatography (1-5% MeOH in DCM as the eluent) to afford **8** white solid (41 mg, 50%).

Pyridin-3-ylmethyl ((*S*)-4,4-dimethyl-1-oxo-1-(((*S*)-1-oxo-3-((*S*)-2-oxopyrrolidin-3-yl)propan-2-yl)amino)pentan-2-yl)carbamate (MPI101)

To a solution of **8** (35 mg, 0.083 mmol) in CH_2Cl_2 (6 mL) was added $NaHCO_3$ (28 mg, 4 equiv) and the Dess-Martin reagent (106 mg, 0.25 mmol, 3 equiv). The resulting mixture was stirred at rt for 12 h. Then the reaction was quenched with a saturated $NaHCO_3$ solution containing 10 % $Na_2S_2O_3$. The layers were separated. The organic layer was then washed with saturated brine solution, dried over anhydrous Na_2SO_4, and concentrated on vacuum. The residue was then purified with flash chromatography afford **MPI101** as white solid (20 mg, 65%). ^{1}H NMR (400 MHz, $CDCl_3$) δ 9.40 (s, 1H), 8.55 (d, *J* = 2.2 Hz, 1H), 8.48 – 8.45 (m, 1H), 8.42 (d, *J* = 6.1 Hz, 1H), 7.62 – 7.59 (m, 1H), 7.20 (d, *J* = 4.7 Hz, 1H), 6.69 (s, 1H), 5.83 (d, *J* = 8.8 Hz, 1H), 5.05 (d, *J* = 4.6 Hz, 2H), 4.32 (m, 1H), 4.24 – 4.19 (m, 1H), 3.32 – 3.16 (m, 3H), 2.36 – 2.23 (m, 2H), 1.83 – 1.70 (m, 3H), 1.46 – 1.39 (m, 1H), 0.90 (s, 9H). ^{13}C NMR (101 MHz, $CDCl_3$) δ 199.77, 180.20, 174.29, 155.66, 149.23, 135.81, 132.22, 123.45, 77.27, 64.30, 57.91, 53.46, 52.92, 46.47, 40.66, 38.34, 30.60, 29.72, 29.68, 28.64. HRMS (ESI): *m*/*z* = 419.2287 $[M + H]^+$. HPLC purity: >99%.

***N*-((*S*)-1-(((*S*)-1-hydroxy-3-((*S*)-2-oxopyrrolidin-3-yl)propan-2-yl)amino)-4,4-dimethyl-1-oxopentan-2-yl)-2-phenylazetidine-1-carboxamide (10)**

To **9** (0.020 g, 0.15 mmol) in DMF (5 mL) were added DIPEA (19.3 mg, 0.15 mmol) and **6** (30.1 mg, 0.15 mmol) and the mixture was stirred at room temperature for 1 h. After 1h compound **5** (0.048 mg, 0.183 mmol) in DMF (5 mL) and DIPEA (0.46 mL, 0.275mmol) were added in dropwise manner. The reaction mixture stirred for 12 h. The mixture was then poured into water (30 mL) and extracted with ethyl acetate (4×20 mL). The organic layer was washed with aqueous hydrochloric acid 10% v/v (2×20 mL), saturated aqueous $NaHCO_3$ (2×20 mL), brine (2×20 mL) and dried over Na_2SO_4. The organic phase was evaporated to dryness and the crude material purified by silica gel column chromatography (1-5% MeOH in DCM as the eluent) to afford **10** white solid (39 mg, 60%). ^{1}H NMR (400 MHz, DMSO) δ 7.52 – 7.37 (m, 2H), 7.34 – 7.13 (m, 5H), 6.01 (m, 1H), 5.07 (m, 1H), 4.60 (m, 1H), 4.04 (m, 1H), 3.94 – 3.60 (m, 3H), 3.29 (m, 1H), 3.24 – 2.86 (m, 3H), 2.58 – 2.47 (m, 1H), 2.23 – 1.98 (m, 2H), 1.91 (t, *J* = 9.2 Hz, 1H), 1.72 (m, 1H), 1.58 – 1.28 (m, 4H), 0.80 (s, 9H).

***N*-((*S*)-4,4-dimethyl-1-oxo-1-(((*S*)-1-oxo-3-((*S*)-2-oxopyrrolidin-3-yl)propan-2-yl)amino)pentan-2-yl)-2-phenylazetidine-1-carboxamide (VB-B-84)**

To a solution of **10** (30 mg, 0.067 mmol) in CH_2Cl_2 (6 mL) was added $NaHCO_3$ (29 mg, 4 equiv) and the Dess-Martin reagent (85 mg, 0.20 mmol, 3 equiv). The resulting mixture was stirred at rt for 12 h. Then the reaction was quenched with a saturated $NaHCO_3$ solution containing 10 % $Na_2S_2O_3$. The layers were separated. The organic layer was then washed with saturated brine solution, dried over anhydrous Na_2SO_4, and concentrated on vacuum. The residue was then purified with flash chromatography afford **MPI105** as white solid (20 mg, 65%). ^{1}H NMR (400 MHz, DMSO) δ 9.40 (s, 1H), 8.36 (dd, *J* = 7.5, 3.8 Hz, 1H), 7.65 (d, *J* = 22.9 Hz, 1H), 7.33 (d, *J* = 5.8 Hz, 5H), 7.25 (d, *J* = 6.0 Hz, 1H), 6.19 (dd, *J* = 20.1, 8.5 Hz, 1H), 5.21 – 5.10 (m, 1H), 4.25 (td, *J* = 8.8, 3.6 Hz, 1H), 4.16 (dt, *J* = 9.4, 3.8 Hz, 1H), 3.95 (q, *J* = 7.0 Hz, 1H), 3.15 (dq, *J* = 20.2, 9.9 Hz, 2H), 2.61 (dd, *J* = 9.8, 5.2 Hz, 1H), 2.28 (tt, *J* = 9.5, 4.9 Hz, 2H), 2.14 (s, 1H), 1.99 (s, 1H), 1.91 (d, *J* = 6.1 Hz, 1H), 1.71 – 1.66 (m, 1H), 1.63 – 1.52 (m, 2H), 0.89 (d, *J* = 13.4 Hz, 9H). ^{13}C NMR (101 MHz, DMSO) δ 201.48, 178.85, 174.72, 174.60, 159.56, 144.10, 143.91, 128.70,

128.64, 127.39, 126.15, 126.01, 63.08, 56.92, 56.72, 51.53, 47.34, 45.49, 30.74, 30.70, 30.14, 30.04, 27.94, 21.53. HRMS (ESI): *m*/*z* = 443.2589[M + H]$^+$. HPLC purity: >99%.

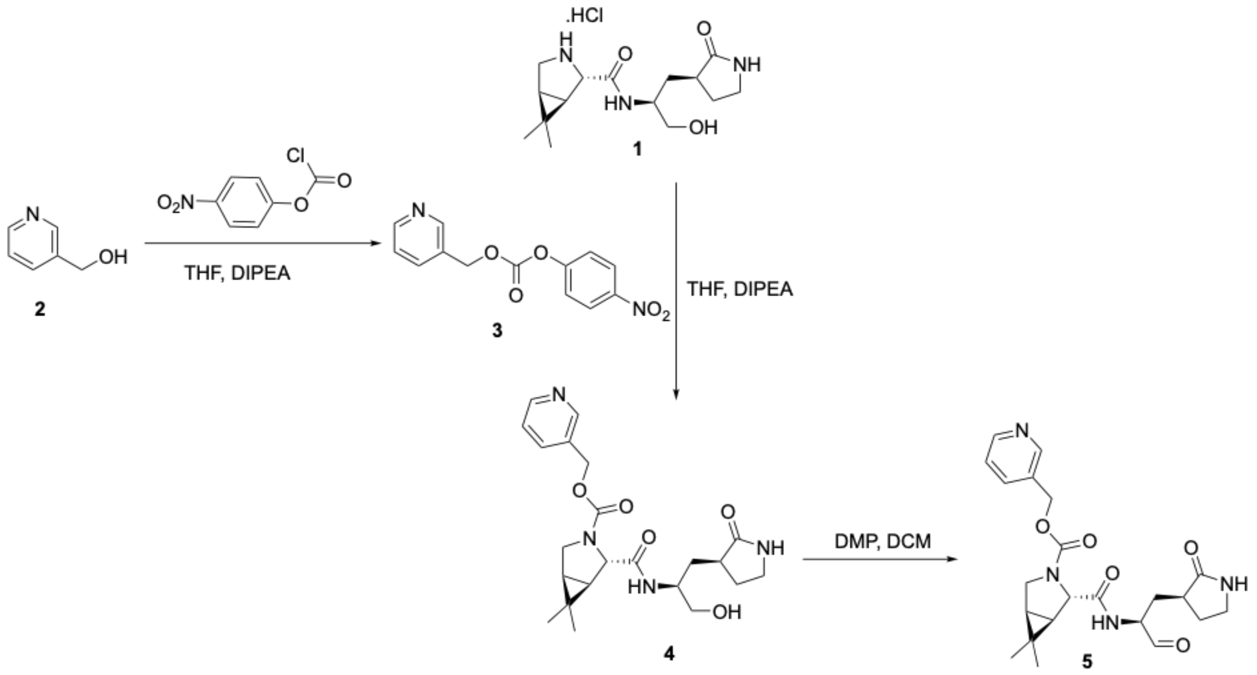

MPI102

Pyridin-3-ylmethyl (1*R*,2*S*,5*S*)-2-(((*S*)-1-hydroxy-3-((*S*)-2-oxopyrrolidin-3-yl)propan-2-yl)carbamoyl)-6,6-dimethyl-3-azabicyclo[3.1.0]hexane-3-carboxylate (4)

To **2** (22 mg, 0.19 mmol) in THF (5 mL) were added DIPEA (0.03 mL, 0.19 mmol) and **PNCF** (38 mg, 0.19 mmol) and the mixture was stirred at room temperature for 1 h. After 1h compound **1** (0.065 mg, 0.19 mmol) in THF (5 mL) and DIPEA (0.046 mL, 0.275mmol) were added to drop wise. The reaction mixture stirred for 12 h. The mixture was then poured into water (30 mL) and extracted with ethyl acetate (4×20 mL). The organic layer was washed with saturated aqueous $NaHCO_3$ (2×20 mL), brine (2×20 mL) and dried over Na_2SO_4. The organic phases were combined, dried over $MgSO_4$, filtered, concentrated to yield **4** as white solid (38 mg, 46%). The crude product is directly used for the next step. To a solution of **4** (30 mg, 0.069 mmol) in CH_2Cl_2 (6 mL) was added $NaHCO_3$ (29 mg, 4 equiv) and the Dess-Martin reagent (88 mg, 0.207 mmol, 3 equiv). The resulting mixture was stirred at rt for 12 h. Then the reaction was quenched with a

saturated $NaHCO_3$ solution containing 10 % $Na_2S_2O_3$. The layers were separated. The organic layer was then washed with saturated brine solution, dried over anhydrous Na_2SO_4, and concentrated in a vacuum. The residue was then purified with flash chromatography for **MPI102** as white solid (10 mg, 33%).^{1}H NMR (400 MHz, DMSO) δ 9.33 (d, *J* = 36.7 Hz, 1H), 8.59 (dd, *J* = 12.6, 7.5 Hz, 1H), 8.44 – 8.40 (m, 1H), 7.67 (ddt, *J* = 6.8, 4.9, 2.4 Hz, 1H), 7.59 (td, *J* = 5.5, 3.2 Hz, 1H), 7.39 – 7.21 (m, 2H), 5.83 – 5.51 (m, 1H), 5.08 – 4.97 (m, 2H), 4.13 – 3.99 (m, 2H), 3.62 – 3.55 (m, 1H), 3.37 – 3.29 (m, 1H), 3.06 – 2.92 (m, 2H), 2.06 (td, *J* = 9.7, 4.8 Hz, 1H), 1.97 – 1.86 (m, 1H), 1.85 – 1.72 (m, 1H), 1.56 (ddt, *J* = 13.5, 6.8, 4.1 Hz, 2H), 1.40 – 1.32 (m, 2H), 0.95 (dd, *J* = 8.1, 1.7 Hz, 3H), 0.83 (dd, *J* = 3.7, 1.9 Hz, 3H).

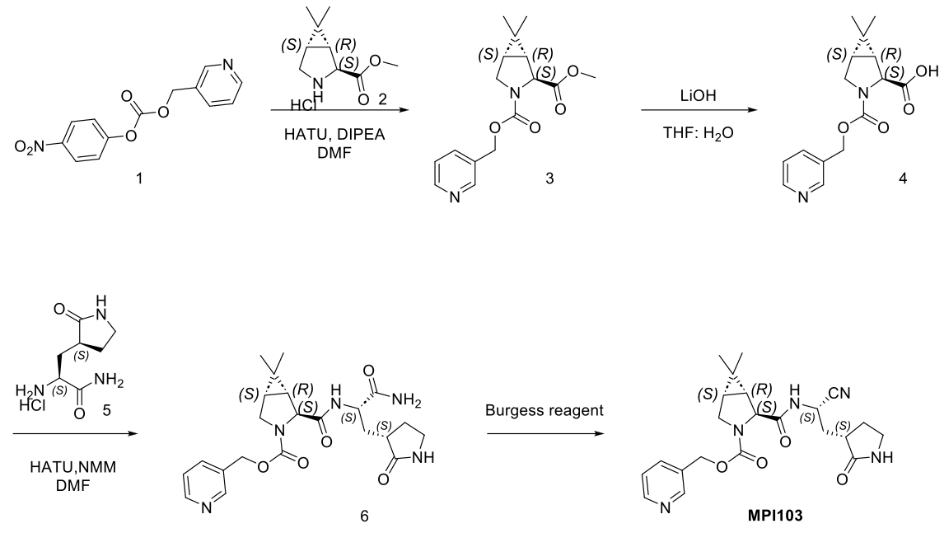

2-Methyl 3-(pyridin-3-ylmethyl) (1R,2S,5S)-6,6-dimethyl-3-azabicyclo[3.1.0]hexane-2,3-dicarboxylate (3)

3 was prepared with methyl (1R,2S,5S)-6,6-dimethyl-3-azabicyclo[3.1.0]hexane-2-carboxylate (2) and 4-nitrophenyl (pyridin-3-ylmethyl) carbonate (1) as a white solid following a similar procedure **b50** (yield 70%). ^{1}H NMR (400 MHz, $CDCl_3$) δ 8.12 – 8.04 (m, 2H), 6.88 – 6.80 (m, 2H), 5.40 – 4.90 (m, 2H), 4.17 (d, *J* = 23.7 Hz, 1H), 3.86 – 3.62 (m, 2.5H), 3.59 (s, 1.5H), 3.44 (dd, *J* = 19.5, 10.9 Hz, 1H), 1.36 (d, *J* = 3.9 Hz, 2H), 0.98 (s, 3H), 0.89 (d, *J* = 2.3 Hz, 3H).

(1R,2S,5S)-6,6-Dimethyl-3-((pyridin-3-ylmethoxy)carbonyl)-3-azabicyclo[3.1.0]hexane-2-carboxylic acid 4)

4 was prepared as a white solid following a similar procedure b51. Directly used for further steps without purification.

Pyridin-3-ylmethyl (1R,2S,5S)-2-(((S)-1-amino-1-oxo-3-((S)-2-oxopyrrolidin-3-yl)propan-2-yl)carbamoyl)-6,6-dimethyl-3-azabicyclo[3.1.0]hexane-3-carboxylate (6)

6 was prepared as a white solid following a similar procedure b50.

Pyridin-3-ylmethyl (1R,2S,5S)-2-(((S)-1-cyano-2-((S)-2-oxopyrrolidin-3-yl)ethyl)carbamoyl)-6,6-dimethyl-3-azabicyclo[3.1.0]hexane-3-carboxylate (MPI103)

Methyl N-(triethylammoniosulfonyl)carbamate (Burgess reagent; 135 mg, 0.566 mmol) was added to a solution of **6** (100 mg, 0.227 mmol) in dichloromethane (3 ml). After the reaction mixture had been stirred at rt for 5 h. The reaction mixture was quenched by a mixture of saturated aqueous sodium bicarbonate solution (20 ml) and saturated aqueous sodium chloride solution (10 ml). The separated organic phase was concentrated. The residue was then purified with flash chromatography (0-10% MeOH in DCM as the eluent) to afford **MPI103** as white solid (20 mg, 21%). As a 1:1 isomers. ^{1}H NMR (400 MHz, DMSO) δ 9.00 (t, *J* = 8.0 Hz, 1H), 8.58 – 8.47 (m, 2H), 7.78 – 7.62 (m, 2H), 7.38 (ddd, *J* = 13.1, 7.7, 4.8 Hz, 1H), 5.12 (s, 1H), 5.05 (s, 1H), 4.97 (p, *J* = 7.7 Hz, 1H), 4.06 (s, 0.5H), 4.01 (s, 0.5H), 3.73 – 3.61 (m, 1H), 3.49 – 3.35 (m, 1H), 3.19 – 3.00 (m, 2H), 2.26 – 2.01 (m, 2H), 1.85 – 1.55 (m, 2H), 1.52 – 1.42 (m, 1H), 1.33 (dd, *J* = 10.0, 7.5 Hz, 1H), 1.18 (t, *J* = 7.3 Hz, 1H), 1.03 (s, 3H), 0.89 (s, 3H). HRMS (ESI): *m*/*z* = 426.2133 [M + H]$^+$. HPLC purity: >99%.

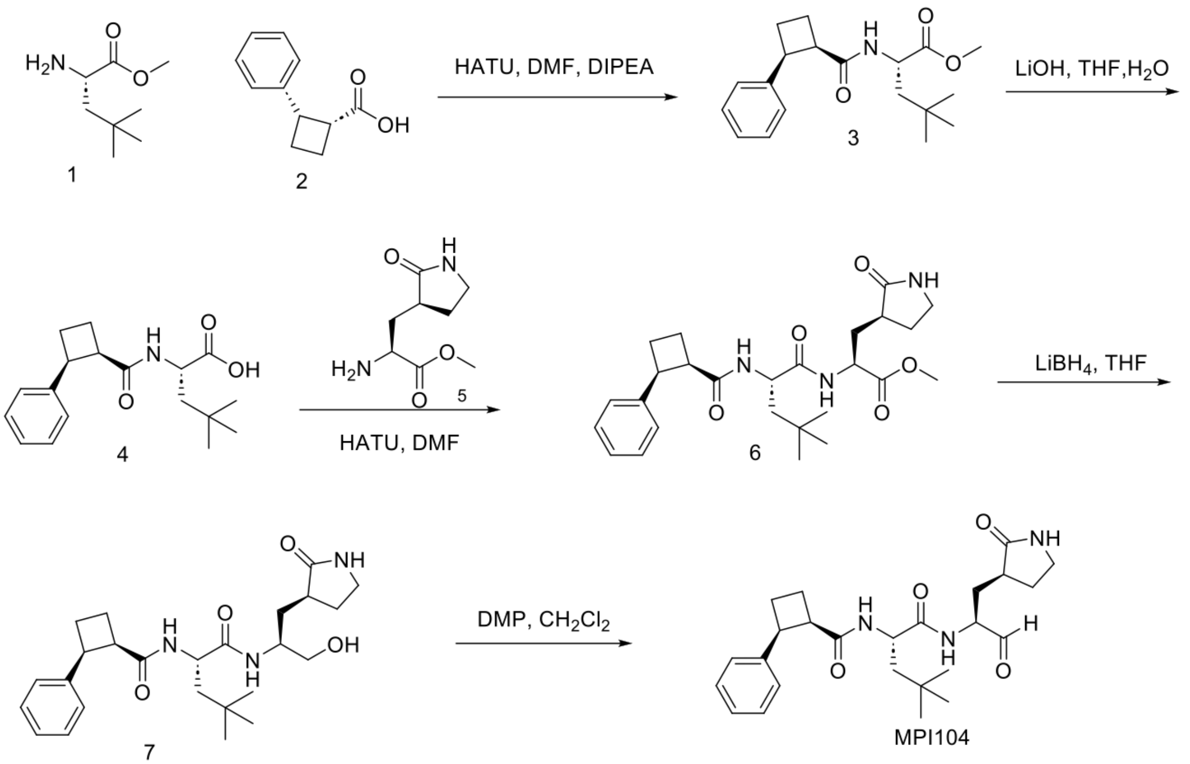

Methyl (S)-4,4-dimethyl-2-((1R,2S)-2-phenylcyclobutane-1-carboxamido)pentanoate (3)

3 was prepared with methyl (S)-2-amino-4,4-dimethylpentanoate (1) and (1R,2S)-2-phenylcyclobutane-1-carboxylic acid (2) as a white solid following a similar procedure **b50** (yield 70%). ^{1}H NMR (400 MHz, $CDCl_3$) δ 7.34 – 7.21 (m, 5H), 5.36 (d, J = 8.7 Hz, 1H), 4.43 (ddd, J = 8.7, 7.7, 5.0 Hz, 1H), 3.41 – 3.30 (m, 1H), 2.68 – 2.55 (m, 1H), 2.48 – 2.17 (m, 2H), 1.61 – 1.51 (m, 2H), 1.30 – 1.18 (m, 2H), 0.85 (s, 9H).

(S)-4,4-Dimethyl-2-((1R,2S)-2-phenylcyclobutane-1-carboxamido)pentanoic acid (4)

4 was prepared as a white solid following a similar procedure b51. Directly used for further step without purification.

Methyl (S)-2-((S)-4,4-dimethyl-2-((1R,2S)-2-phenylcyclobutane-1-carboxamido)pentanamido)-3-((S)-2-oxopyrrolidin-3-yl)propanoate (6)

6 was prepared with methyl (S)-2-amino-3-((S)-2-oxopyrrolidin-3-yl)propanoate (5) and (1R,2S)-2-phenylcyclobutane-1-carboxylic acid (4) as a white solid following a similar procedure b50. ^{1}H NMR (400 MHz, DMSO) δ 8.18 (d, *J* = 7.4 Hz, 1H), 7.80 (d, *J* = 8.8 Hz, 1H), 7.68 (s, 1H), 7.25 – 7.02 (m, 5H), 4.29 – 4.13 (m, 2H), 3.81 (q, *J* = 9.0 Hz, 1H), 3.59 (s, 3H), 3.44 (q, *J* = 7.5 Hz, 1H), 3.21 – 3.04 (m, 2H), 2.27 – 1.91 (m, 7H), 1.63 – 1.47 (m, 3H), 1.39 – 1.23 (m, 2H), 0.82 (s, 9H).

(1R,2S)-N-((S)-1-(((S)-1-Hydroxy-3-((S)-2-oxopyrrolidin-3-yl)propan-2-yl)amino)-4,4-dimethyl-1-oxopentan-2-yl)-2-phenylcyclobutane-1-carboxamide (7)

7 was prepared as a white solid following a similar procedure b55.

(1R,2S)-N-((S)-4,4-Dimethyl-1-oxo-1-(((S)-1-oxo-3-((S)-2-oxopyrrolidin-3-yl)propan-2-yl)amino)pentan-2-yl)-2-phenylcyclobutane-1-carboxamide (MPI104)

MPI104 was prepared as a white solid following a similar procedure **b57**. ^{1}H NMR (400 MHz, $CDCl_3$) δ 9.24 (d, *J* = 0.9 Hz, 1H), 7.77 (d, *J* = 6.0 Hz, 1H), 7.25 – 7.01 (m, 6H), 6.58 (s, 1H), 5.97 (d, *J* = 8.8 Hz, 1H), 4.43 (td, *J* = 8.4, 4.2 Hz, 1H), 4.04 – 3.94 (m, 1H), 3.85 (q, *J* = 8.5 Hz, 1H), 3.38 – 3.14 (m, 4H), 2.61 – 2.47 (m, 1H), 2.35 – 2.08 (m, 6H), 1.96 – 1.62 (m, 4H), 1.18 (dd, *J* = 14.4, 8.1 Hz, 1H), 0.80 (s, 9H). ^{13}C NMR (101 MHz, $CDCl_3$) δ 200.15, 179.62, 173.81, 172.41, 141.49, 128.11, 127.37, 126.23, 57.57, 50.25, 46.80, 45.79, 43.00, 42.52, 40.54, 38.07, 30.52, 29.89, 29.78, 29.59, 29.28, 28.61, 25.00, 21.31.HRMS (ESI): *m/z* = 442.2680 $[M + H]^{+}$. HPLC purity: >99%.

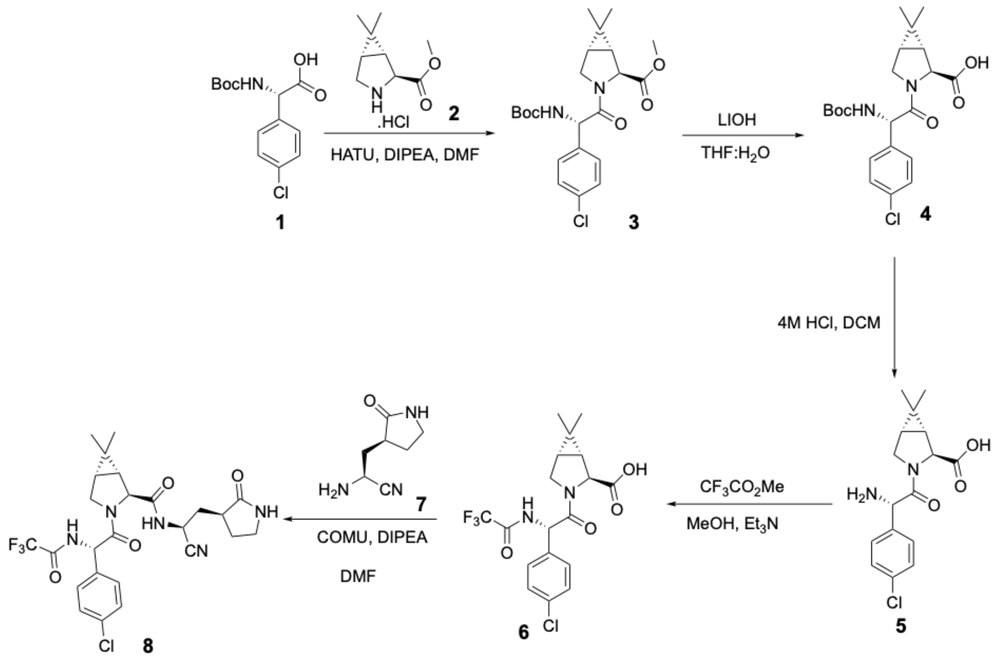

MPI106

(1*R*,2*S*,5*S*)-3-((*S*)-2-(4-Chlorophenyl)-2-(2,2,2-trifluoroacetamido)acetyl)-6,6-dimethyl-3-azabicyclo[3.1.0]hexane-2-carboxylic acid (6)

To a solution of 1 (1.75 mmol, 0.5 g) and 2 (1.75 mmol, 0.35 g) in anhydrous DMF (20 mL) was added DIPEA (5.25 mmol,0.89 mL) and was cooled to 0 °C. HATU (2.63 mmol, 1.0 g) was added to the solution under 0 °C and then stirred at room temperature overnight. The reaction mixture was then diluted with ethyl acetate (50 mL) and washed with saturated $NaHCO_3$ solution (2×20 mL), 1 M HCl solution (2×20 mL), and saturated brine solution (2×20 mL) sequentially. The organic layer was dried over anhydrous Na_2SO_4 and then concentrated *in a vacuum*. The organic phases were combined, dried over $MgSO_4$, filtered, concentrated to yield 3 as white solid (530 mg, 70%). The crude product is directly used for the next step. The peptide 3 (500 mg, 1.14 mmol) was dissolved in THF/H_2O (1:1, 20 mL).LiOH (72 mg, 1.71 mmol) was added at 0 °C. The mixture was stirred at room temperature overnight. Then THF was removed *on vacuum* and the aqueous layer was acidified with 1 M HCl and extracted with dichloromethane (3 x 10 mL). The organic phases were combined, dried over $MgSO_4$, filtered, concentrated to yield 4 as white solid (380 mg, 80%). The crude product is directly used for the next step. To a stirred solution of 4 (350 mg, 0.83 mmol) in 1,4-Dioxane (1 mL) at 0 °C was added 4N HCl (1.4 mL). Reaction mixture

was stirred at rt for 3 h. After completion of reaction, solvent was concentrated in a vacuum. The residue was used in the next step without further purification (400 mg).

(1*R*,2*S*,5*S*)-3-((*S*)-2-(4-Chlorophenyl)-2-(2,2,2-trifluoroacetamido)acetyl)-*N*-((*S*)-1-cyano-2-((*S*)-2-oxopyrrolidin-3-yl)ethyl)-6,6-dimethyl-3-azabicyclo[3.1.0]hexane-2-carboxamide (8): To a solution of 6 (0.23 mmol, 0.1 g) and 7 (0.23 mmol, 0.05 g) in anhydrous DMF (20 mL) was added DIPEA (0.69 mmol, 0.11 mL) and was cooled to 0 °C. COMU (0.36 mmol, 0.15 g) was added to the solution under 0 °C and then stirred at room temperature 3 h. The reaction mixture was then diluted with ethyl acetate (50 mL) and washed with saturated $NaHCO_3$ solution (2×20 mL), 1 M HCl solution (2×20 mL), and saturated brine solution (2×20 mL) sequentially. The organic layer was dried over anhydrous Na_2SO_4 and then concentrated *in a vacuum*. The residue was then purified with flash chromatography (0-6% MeOH in DCM as the eluent) to afford MPI106 as white solid (0.082 mg, 62%). ^{1}H NMR (400 MHz, DMSO) δ 9.96 (d, *J* = 6.5 Hz, 1H), 8.95 (d, *J* = 8.1 Hz, 1H), 7.64 (s, 1H), 7.45 – 7.27 (m, 4H), 5.62 (d, *J* = 6.6 Hz, 1H), 4.90 (ddd, *J* = 9.5, 8.0, 6.5 Hz, 1H), 4.15 (s, 1H), 3.63 (d, *J* = 10.5 Hz, 1H), 3.22 – 2.99 (m, 3H), 2.32 (qd, *J* = 9.5, 5.3 Hz, 1H), 2.15 – 1.97 (m, 2H), 1.77 – 1.60 (m, 2H), 1.42 (dd, *J* = 7.6, 5.3 Hz, 1H), 1.22 (d, *J* = 7.6 Hz, 1H), 0.95 (s, 3H), 0.89 (s, 3H). ^{13}C NMR (101 MHz, DMSO) δ 178.02, 170.97, 166.43, 156.50, 133.79, 133.15, 131.41, 128.90, 119.97, 117.61, 114.75, 66.37, 60.75, 55.72, 47.02, 38.76, 38.43, 37.52, 34.24, 30.58, 27.68, 27.56, 26.24, 19.35, 13.02. HRMS(ESI): *m*/*z* = 555.1712 $[M + H]^+$. HPLC purity: >99%.

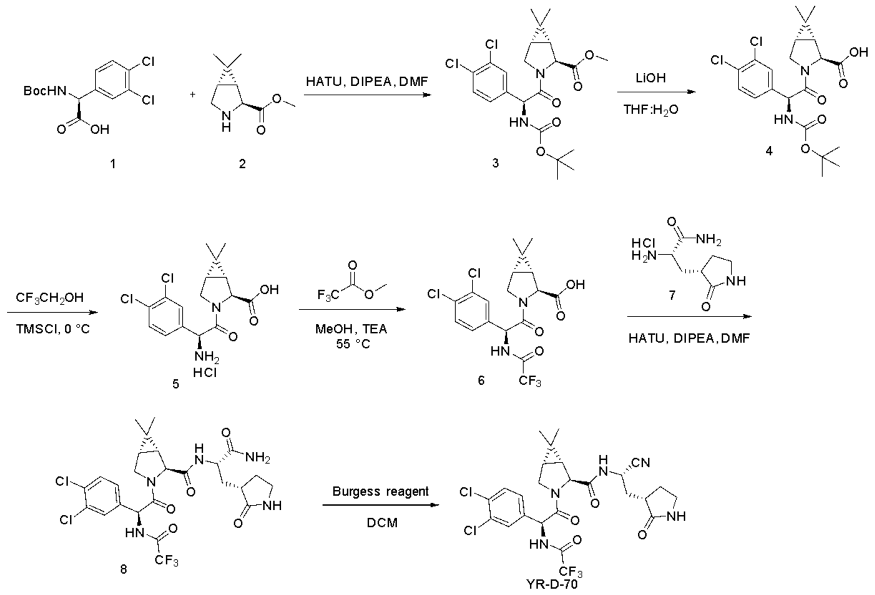

MPI108

Methyl(1R,2S,5S)-3-((S)-2-((tert-butoxycarbonyl)amino)-2-(3,4-dichlorophenyl)acetyl)-6,6-dimethyl-3-azabicyclo[3.1.0]hexane-2-carboxylate (3)

Compound **3** prepared as a white solid following similar procedure **b50** (yield:60%). ^{1}H NMR (400 MHz, DMSO) δ 7.69 – 7.56 (m, 3H), 7.46 (t, *J* = 8.4 Hz, 1H), 7.38 (ddd, *J* = 8.4, 4.1, 2.1 Hz, 1H), 5.39 (t, *J* = 8.4 Hz, 1H), 4.25 (s, 1H), 3.82 – 3.71 (m, 1H), 3.70 – 3.63 (m, 2H), 3.62 (s, 3H), 3.28 (dd, *J* = 10.6, 5.4 Hz, 1H), 1.52 (ddd, *J* = 17.7, 8.4, 4.7 Hz, 1H), 1.42 – 1.34 (m, 10H), 1.01 (d, *J* = 6.0 Hz, 3H), 0.97 – 0.87 (m, 3H).

(1R,2S,5S)-3-((S)-2-((tert-Butoxycarbonyl)amino)-2-(3,4-dichlorophenyl)acetyl)-6,6-dimethyl-3-azabicyclo[3.1.0]hexane-2-carboxylic acid (4)

Compound **4** prepared as a white solid following similar procedure **b51** (yield:74%). The residue was used next step without further purification.

(1R,2S,5S)-3-((S)-2-(3,4-Dichlorophenyl)-2-(2,2,2-trifluoroacetamido)acetyl)-6,6-dimethyl-3-azabicyclo[3.1.0]hexane-2-carboxylic acid (6)

To a solution of **4** (700 mg) of crude material in 2,2,2-trifluoroethanol (10 mL) was added dropwise a TMSCl solution (mL). The resulting solution was stirred at room temperature for 6 h. Then residue was then concentrated *on vacuo* to afford **5** as light-yellow hygroscopic solid. Crude

compound directly used next step. To a 0 °C solution of the HCl salt of **5** (500 mg, 1.31 mmol) in methanol (3 ml) was added triethylamine (0.64 ml, 4.58 mmol), followed by Methyl trifluoroacetate (0.2 ml, 1.96 mmol), whereupon the reaction mixture was allowed to warm to 50 °C, and was stirred for 16 h. It was then concentrated in vacuo at 50 °C, and the residue was diluted with water (10 ml) and adjusted to a pH of 3 to 4 by addition of 1 M HCl. After extraction of the aqueous layer with ethyl acetate (3 x 20 ml), the combined organic layers were washed with saturated aqueous sodium chloride solution (20 ml), dried over sodium sulfate, filtered, and concentrated to afford **6** as a white solid. The residue was used next step without further purification.

(1R,2S,5S)-N-((S)-1-Amino-1-oxo-3-((S)-2-oxopyrrolidin-3-yl)propan-2-yl)-3-((S)-2-(3,4-dichlorophenyl)-2-(2,2,2-trifluoroacetamido)acetyl)-6,6-dimethyl-3-azabicyclo[3.1.0]hexane-2-carboxthede (7)

Compound **7** prepared as a white solid following similar procedure **b50** (yield:56%).

(1R,2S,5S)-N-((S)-1-Cyano-2-((S)-2-oxopyrrolidin-3-yl)ethyl)-3-((S)-2-(3,4-dichlorophenyl)-2-(2,2,2-trifluoroacetamido)acetyl)-6,6-dimethyl-3-azabicyclo[3.1.0]hexane-2-carboxamide (MPI108)

To a stirred solution of **6** (60 mg, 0.10 mmol) in DCM (3 mL) at 0 °C was added Burgess reagent (57 mg, 0.26 mmol) and the mixture was stirred at rt for 2 h. The reaction was quenched with saturated $NaHCO_3$ solution (5 mL) and extracted with DCM (2 × 10 mL). The combined organic layer was washed with brine, dried over $MgSO_4$, and concentrated in a vacuum. The residue was then purified with flash chromatography (0-10% MeOH in Dichloromethane as the eluent) to afford **MPI108** as a yellow solid (35 mg, 60%).^{1}H NMR (400 MHz, DMSO) δ 10.06 (d, *J* = 6.6 Hz, 1H), 9.04 (d, *J* = 8.1 Hz, 1H), 7.77 – 7.63 (m, 3H), 7.43 (dd, *J* = 8.4, 2.1 Hz, 1H), 5.72 (d, *J* = 6.6 Hz, 1H), 5.03 – 4.89 (m, 1H), 3.68 (dd, *J* = 29.2, 12.9 Hz, 1H), 3.39 – 3.24 (m, 1H), 3.22 – 3.04 (m, 3H), 2.45 – 2.33 (m, 1H), 2.25 – 2.04 (m, 2H), 1.85 – 1.65 (m, 2H), 1.56 – 1.35 (m, 1H), 1.34 – 1.25 (m, 1H), 1.03 (s, 3H), 0.97 (s, 2H). HRMS (ESI): *m/z* = 589.1262 $[M + H]^+$. HPLC purity: 97.6%

In Vitro M^{Pro} Inhibition Potency Characterization of Inhibitors

For most inhibitors, the assay was conducted using 20 nM M^{Pro} and 10 μM Sub3.[4] We dissolved all inhibitors in DMSO as 10 mM stock solutions. Sub3 was dissolved in DMSO as a 1 mM stock solution and diluted 100 times in the final assay buffer containing 10 mM $Na_xH_yPO_4$, 10 mM NaCl, 0.5 mM EDTA, and 1.25% DMSO at pH 7.6. M^{Pro} and an inhibitor were incubated in the final assay buffer for 30 min before adding the substrate to initiate the reaction catalyzed by M^{Pro}. The production format was monitored in a fluorescence plate reader with excitation at 336 nm and emission at 490 nm. More assay details can be found in ref. 4.

Cellular M^{Pro} Inhibition Potency Characterization of Inhibitors

Cellular M^{Pro} inhibition potency for all tested inhibitors was characterized according to the protocol shown in a previous report.[5] HEK 293T/17 cells were grown in high-glucose DMEM with GlutaMAX supplement and 10% fetal bovine serum in 10 cm culture plates under 37 °C and 5% CO_2 to ~80–90% and then transfected with the pLVX-MProeGFP-2 plasmid. For each transfection, 30 mg/mL polyethylenimine and a total of 8 μg of the plasmid in 500 μL of the Opti-MEM medium were used. Cells were incubated with transfection reagents overnight. On the second day, the medium was removed, and cells were washed with a PBS buffer and then digested with 0.05% trypsin–EDTA. Cells were collected by centrifugation and then resuspended in the original growth medium to a cell density of 5×10^5 cells/mL in 500 μL in a 48-well plate. A compound solution of 100 μL was then added to the growth medium. These cells were incubated under 37 °C and 5% CO_2 for 3 days before their flow cytometry analysis.

Recombinant M^{Pro} protein expression and purification

The pET28a-His-SUMO-M^{Pro} expression and purification were performed according to our previous report.[6] The pET28a-His-SUMO-M^{Pro} construct was transformed into *Escherichia coli* BL21(DE3) cells. Transformed cells were cultured at 37°C in 2× YT medium with kanamycin (50 g/mL) until the opeitcal density at 600 nm (OD_{600}) reached 0.6 and then induced with 1 mM isopropyl-D-1-thiogalactoside (IPTG) at 37°C. After 3 h, cells were harvested and lysed in buffer

A (20 mM Tris, 100 mM NaCl, 10 mM imidazole, pH 8.0). The supernatant was loaded onto a nickel-chelating column (GenScript) washed with buffer A, followed by elution with buffer B (20 mM Tris, 100 mM NaCl, 250 mM imidazole, pH 8.0). The eluted protein solution was desalted to buffer C (20 mM Tris, 10 mM NaCl, pH 8.0) using a HiPrep 26/10 desalting column (GE Healthcare). The His-SUMO-M^{Pro} proteins were digested with SUMO protease overnight at 4°C. The digested protein was applied to the nickel-chelating column again to remove the His-tagged SUMO protease, the His-SUMO tag, and the expressed protein with uncleaved His-SUMO tag. The tag-free M^{Pro} protein was loaded onto a HiPrep 16/60 Sephacryl S-100 HR size-exclusion column (GE Healthcare) pre-equilibrated with buffer D (20 mM Tris, 100 mM NaCl, 1 mM EDTA, pH 7.8). The eluted M^{Pro} protein was stored in buffer D at −80°C for further use.

X-ray Crystallography Analysis

The production of crystals of apo M^{Pro} and M^{Pro}–inhibitor complexes followed the previous protocols with the crystal growth conditions of 0.1 M Bis-Tris, pH 6.5, 16% w/v PEG10k.[6] The data for M^{Pro} with MI-14, MI-30, MI-31, MPI50, MPI51, MPI57, MPI101, and MPI105 were collected on a Bruker Photon II detector, and the data for M^{Pro} with MPI52, MPI54, MPI69, MPI94, MPI95, and MPI97 were collected at the Advanced Light Source (ALS) beamline 8.2.2 using a Pilatus3 6 M detector. The diffraction data were indexed, integrated, and scaled with iMosflm or PROTEUM3.[7] The structure was determined by molecular replacement using the structure model of the free enzyme of the SARS-CoV-2 M^{pro} (PDB entry 7JPY) as the search model using Phaser in the Phenix package. JLigand and Sketcher from the CCP4 suite were employed for the generation of PDB and geometric restraints for the inhibitors. The inhibitors were built into the $F_o - F_c$ density using Coot. All of the structures were refined with real-space refinement in Phenix.[8-9] Details of data quality and structure refinement are summarized in Table S1. All structural figures were generated with PyMOL (https://www.pymol.org).

Live Virus Antiviral Tests

SARS-CoV-2 delta variant hCoV-19/USA/MD-HP05647/2021 (BEI Resources, NR-55672) was propagated in A549-hACE2 cells (BEI Resources NR-53522) for antiviral testing, at 37ºC in an air-jacketed incubator, with 5% CO_2 and >90% relative humidity, under BSL-3

conditions at the Texas A&M Global Health Research Complex. A low-dose, multi-step growth protocol was used for live virus EC_{50} assays. Briefly, A549-hACE2 cells were cultured in DMEM supplemented with 10% fetal bovine serum overnight. Approximately 5×10^4 A549-hACE2 cells were inoculated by adding 10^3 infectious units of SARS-CoV-2, as determined by tissue culture infectious dose 50% (TCID50) assay, and incubated at 37ºC for 1 hour. Cells were then aspirated and rinsed three times with room temperature phosphate buffered saline, to remove residual inoculum, before replacing DMEM with 10% FBS. Serial three-fold dilutions of candidate antivirals were made in DMEM with 10% FBS and added to three replicate wells per treatment condition. Infected, treated cells were then cultured for 72h at 37ºC, 5% CO_2. At 48h, and 72h after inoculation, 50 microliters of tissue culture medium were removed from each sample, for quantitative SARS-CoV-2 reverse transcriptase quantitative PCR (RT-qPCR). After 72h, cell culture supernatant was removed by aspiration, cells were fixed in 10% formalin, 1× phosphate buffered saline for at least 30 min, then stained with crystal violet in order to qualitatively assess cytopathic effect. EC_{50} values were calculated from the slope and intercept of log-transformed RT-qPCR results, at the point where the linear portion of the transformed dose-response curve showed 50% reduced growth compared to infected, untreated controls. Samples were processed and RT-qPCR was performed as per protocol established previously.[10] Samples were diluted 1:1 in 2× Tris-borate-EDTA [TBE] containing 1% Tween-20 and heated at 95°C for 15 min to lyse and inactivate virions. RT-qPCR screening was performed using the CDC N1 oligonucleotide pair/FAM probe (CDC N1-F, 5′-GACCCCAAAATCAGCGAAAT-3′; CDC N1-R, 5′-TCTGGTTACTGCCAGTTGAATCTG-3′; and Probe CDC N1, 5′ FAM-ACCCCGCATTACGTTTGGTGGACC-BHQ1 3′) and the Luna Universal Probe one-step RT-qPCR kit (catalog no. E3006; New England Biolabs). A 20-μL RT-qPCR mixture contained 7 μL of sample, 0.8 μL each of forward and reverse oligonucleotides (10 μM), 0.4 μL of probe (10 μM), and 11 μL of NEB Luna one-step RT-qPCR 2× master mix. Samples were incubated at 55°C for 10 min for cDNA synthesis, followed by 95°C for 1 min (1 cycle), then 41 cycles of 95°C for 10 s and 60°C for 30 s. Genome copies were quantitated relative to quantitative PCR control RNA from heat-inactivated SARS-Related Coronavirus 2, Isolate USA-WA1/2020 (BEI Resources, NR-52347).

References:

1. Geng, Z. Z.; Atla, S.; Shaabani, N.; Vulupala, V.; Yang, K. S.; Alugubelli, Y. R.; Khatua, K.; Chen, P. H.; Xiao, J.; Blankenship, L. R.; Ma, X. R.; Vatansever, E. C.; Cho, C. D.; Ma, Y.; Allen, R.; Ji, H.; Xu, S.; Liu, W. R., A Systematic Survey of Reversibly Covalent Dipeptidyl Inhibitors of the SARS-CoV-2 Main Protease. *J. Med. Chem.* **2023,** *66* (16), 11040-11055.
2. Qiao, J.; Li, Y. S.; Zeng, R.; Liu, F. L.; Luo, R. H.; Huang, C.; Wang, Y. F.; Zhang, J.; Quan, B.; Shen, C.; Mao, X.; Liu, X.; Sun, W.; Yang, W.; Ni, X.; Wang, K.; Xu, L.; Duan, Z. L.; Zou, Q. C.; Zhang, H. L.; Qu, W.; Long, Y. H.; Li, M. H.; Yang, R. C.; Liu, X.; You, J.; Zhou, Y.; Yao, R.; Li, W. P.; Liu, J. M.; Chen, P.; Liu, Y.; Lin, G. F.; Yang, X.; Zou, J.; Li, L.; Hu, Y.; Lu, G. W.; Li, W. M.; Wei, Y. Q.; Zheng, Y. T.; Lei, J.; Yang, S., SARS-CoV-2 M(pro) inhibitors with antiviral activity in a transgenic mouse model. *Science* **2021,** *371* (6536), 1374-1378.
3. Khatua, K.; Alugubelli, Y. R.; Yang, K. S.; Vulupala, V. R.; Blankenship, L. R.; Coleman, D. D.; Atla, S.; Chaki, S. P.; Geng, Z. Z.; Ma, X. R.; Xiao, J.; Chen, P.-H. C.; Cho, C.-C. D.; Vatansever, E. C.; Ma, Y.; Yu, G.; Neuman, B. W.; Xu, S.; Liu, W. R., An Azapeptide Platform in Conjunction with Covalent Warheads to Uncover High-Potency Inhibitors for SARS-CoV-2 Main Protease. *bioRxiv* **2023**, 2023.04.11.536467.
4. Vatansever, E. C.; Yang, K. S.; Drelich, A. K.; Kratch, K. C.; Cho, C. C.; Kempaiah, K. R.; Hsu, J. C.; Mellott, D. M.; Xu, S.; Tseng, C. K.; Liu, W. R., Bepridil is potent against SARS-CoV-2 in vitro. *Proc. Natl. Acad. Sci. U. S. A.* **2021,** *118* (10), e2012201118.
5. Cao, W.; Cho, C. D.; Geng, Z. Z.; Shaabani, N.; Ma, X. R.; Vatansever, E. C.; Alugubelli, Y. R.; Ma, Y.; Chaki, S. P.; Ellenburg, W. H.; Yang, K. S.; Qiao, Y.; Allen, R.; Neuman, B. W.; Ji, H.; Xu, S.; Liu, W. R., Evaluation of SARS-CoV-2 Main Protease Inhibitors Using a Novel Cell-Based Assay. *ACS Cent. Sci.* **2022,** *8* (2), 192-204.
6. Yang, K. S.; Ma, X. R.; Ma, Y.; Alugubelli, Y. R.; Scott, D. A.; Vatansever, E. C.; Drelich, A. K.; Sankaran, B.; Geng, Z. Z.; Blankenship, L. R.; Ward, H. E.; Sheng, Y. J.; Hsu, J. C.; Kratch, K. C.; Zhao, B.; Hayatshahi, H. S.; Liu, J.; Li, P.; Fierke, C. A.; Tseng, C. K.; Xu, S.; Liu, W. R., A Quick Route to Multiple Highly Potent SARS-CoV-2 Main Protease Inhibitors. *ChemMedChem* **2021,** *16* (6), 942-948.

7. Powell, H. R.; Battye, T. G. G.; Kontogiannis, L.; Johnson, O.; Leslie, A. G. W., Integrating macromolecular X-ray diffraction data with the graphical user interface iMosflm. *Nat. Protoc.* **2017,** *12* (7), 1310-1325.
8. Potterton, L.; Agirre, J.; Ballard, C.; Cowtan, K.; Dodson, E.; Evans, P. R.; Jenkins, H. T.; Keegan, R.; Krissinel, E.; Stevenson, K.; Lebedev, A.; McNicholas, S. J.; Nicholls, R. A.; Noble, M.; Pannu, N. S.; Roth, C.; Sheldrick, G.; Skubak, P.; Turkenburg, J.; Uski, V.; von Delft, F.; Waterman, D.; Wilson, K.; Winn, M.; Wojdyr, M., CCP4i2: the new graphical user interface to the CCP4 program suite. *Acta Crystallogr D Struct Biol* **2018,** *74* (Pt 2), 68-84.
9. Chaikuad, A.; Petros, A. M.; Fedorov, O.; Xu, J.; Knapp, S., Structure-based approaches towards identification of fragments for the low-druggability ATAD2 bromodomain. *Medchemcomm* **2014,** *5* (12), 1843-1848.
10. Chaki, S. P.; Kahl-McDonagh, M. M.; Neuman, B. W.; Zuelke, K. A., Receptor-Binding-Motif-Targeted Sanger Sequencing: a Quick and Cost-Effective Strategy for Molecular Surveillance of SARS-CoV-2 Variants. *Microbiol Spectr* **2022,** *10* (3), e0066522.

Supplementary Figures

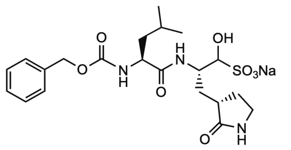

GC376

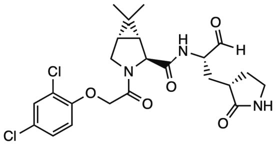

MI-14

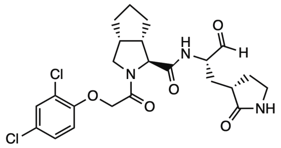

MI-30

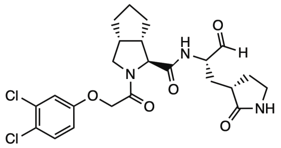

MI-31

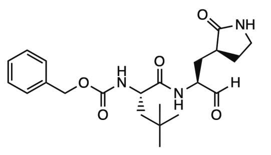

MPI50

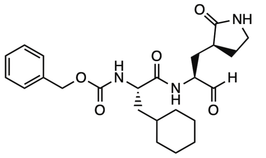

MPI51

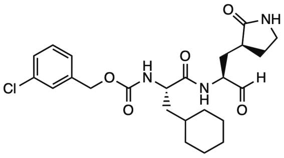

MPI52

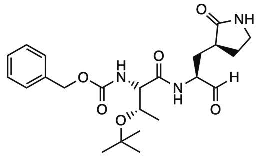

MPI54

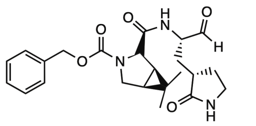

MPI57

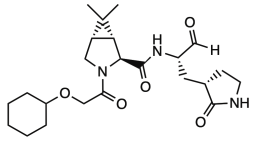

MPI64

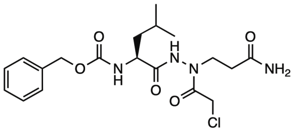

MPI68

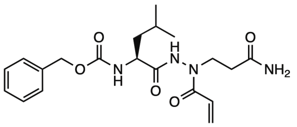

MPI69

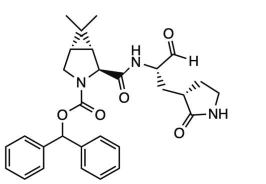

MPI94

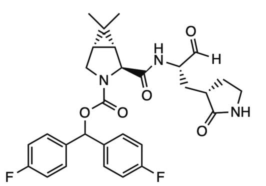

MPI95

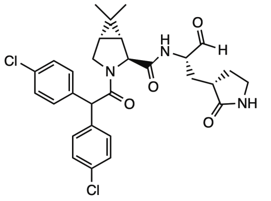

MPI96

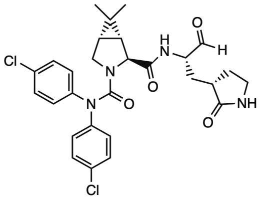

MPI97

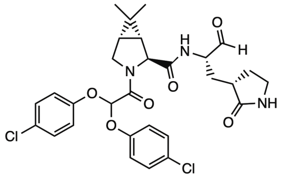

MPI98

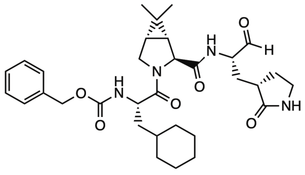

MPI100

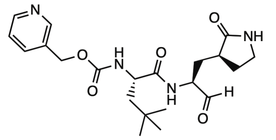

MPI101

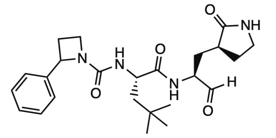

MPI105

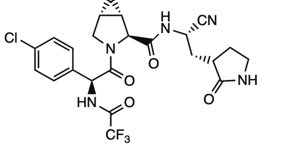

MPI106

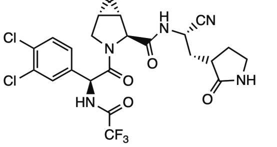

MPI108

Figure S1: Structures of All inhibitors that were discussed.

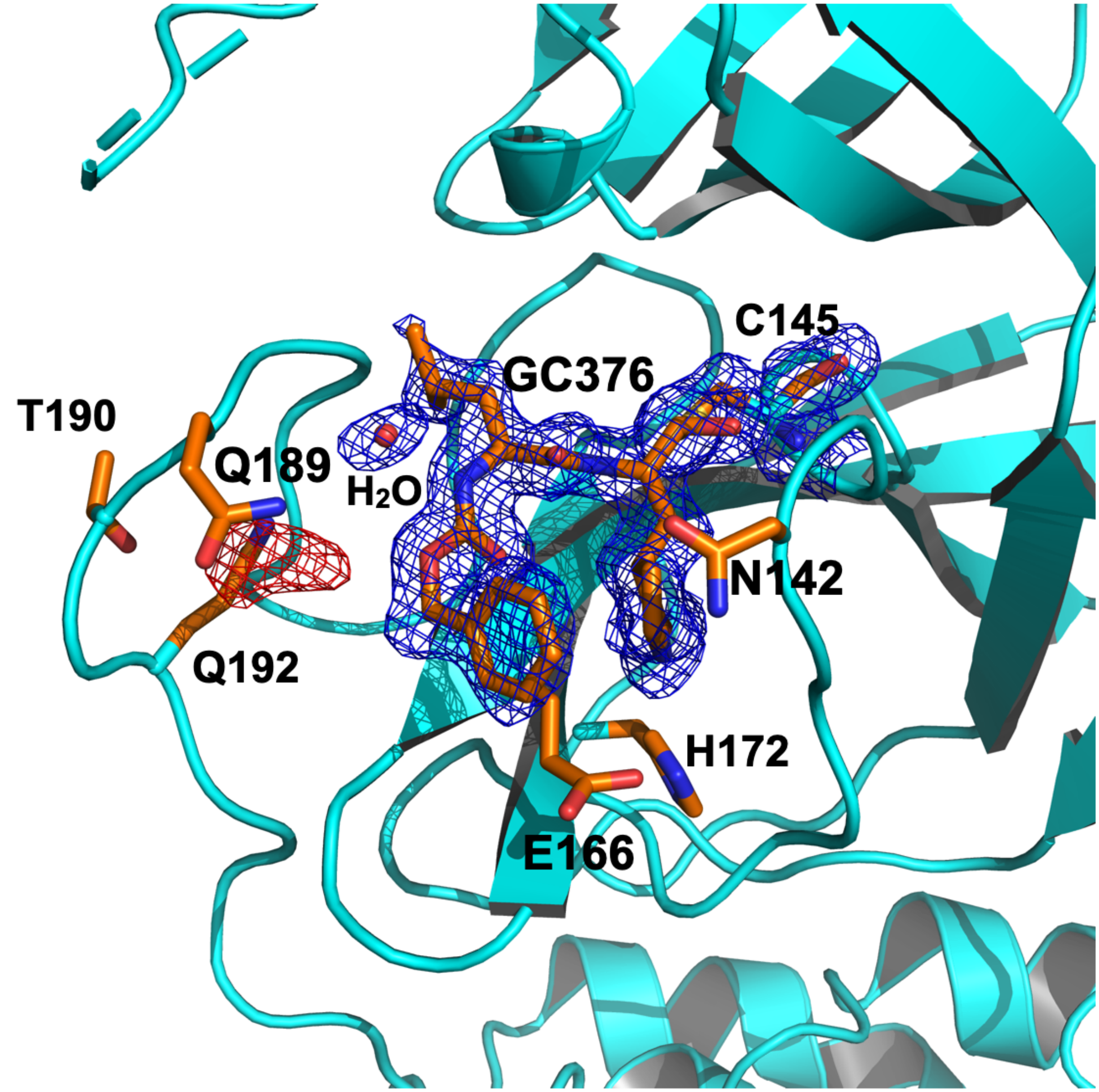

Figure S2: The 2Fo-Fc map around GC376 and Cys145 in the active site of the M^{Pro}-GC376 complex. The map is contoured at the 1σ level. The Fo-Fc map at the S4 site is shown as well, contoured at the 2σ level.

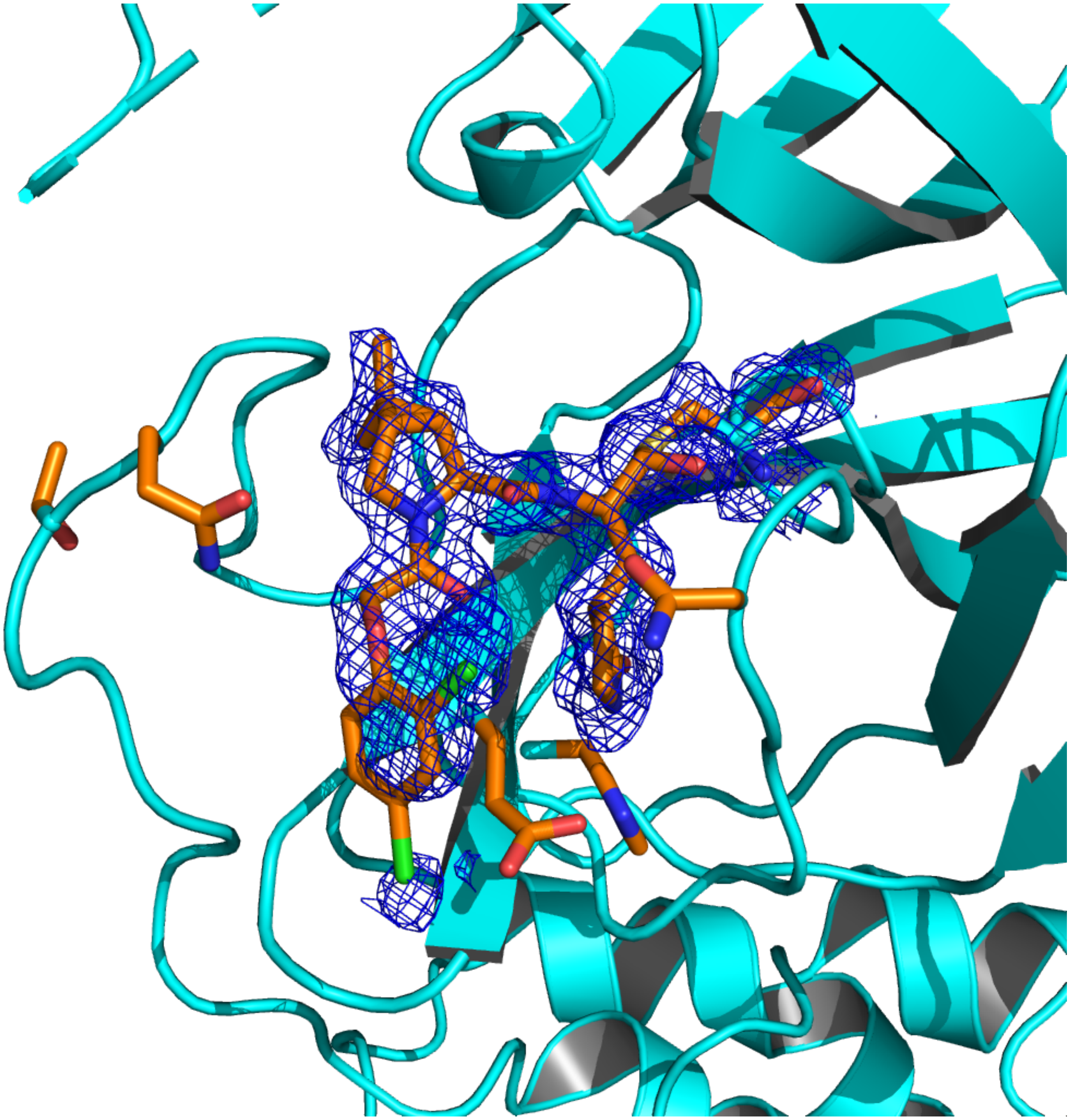

Figure S3: The 2Fo-Fc map around MI-14 and Cys145 in the active site of the M^{Pro}-MI-14 complex. The map is contoured at the 1σ level.

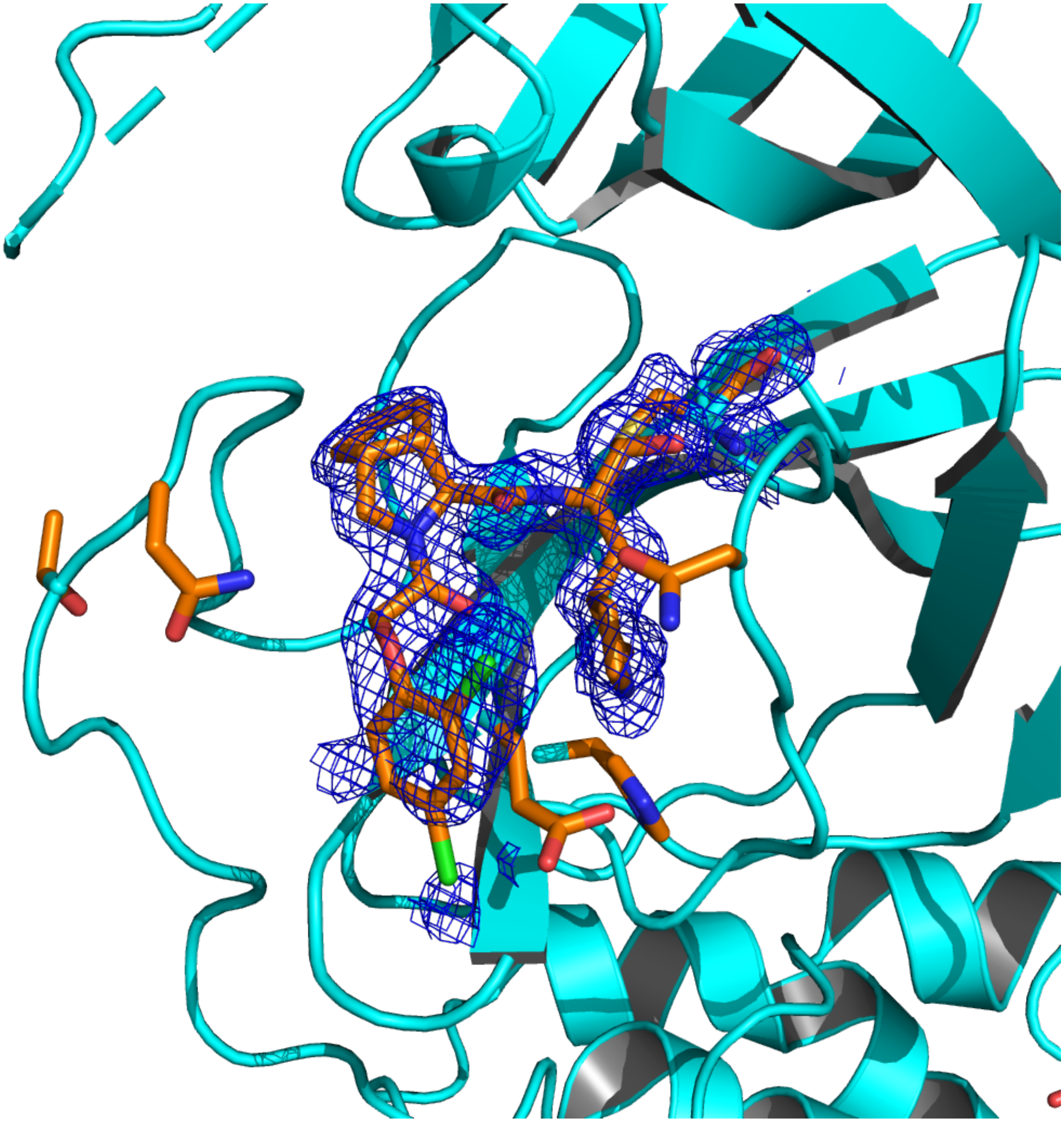

Figure S4: The 2Fo-Fc map around MI-30 and Cys145 in the active site of the M^{Pro}-MI-30 complex. The map is contoured at the 1σ level.

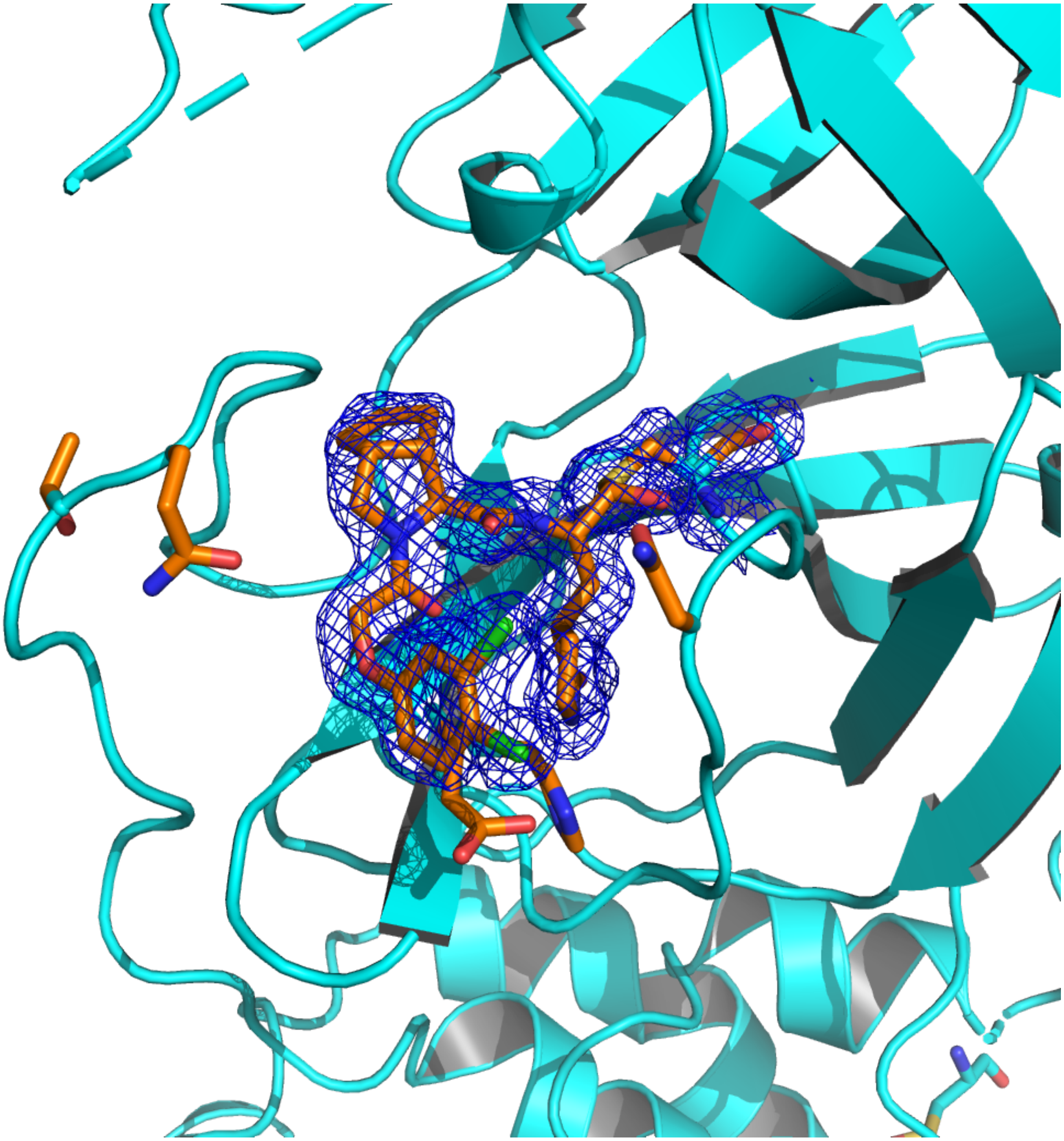

Figure S5: The 2Fo-Fc map around MI-31 and Cys145 in the active site of the M^{Pro}-MI-31 complex. The map is contoured at the 1σ level.

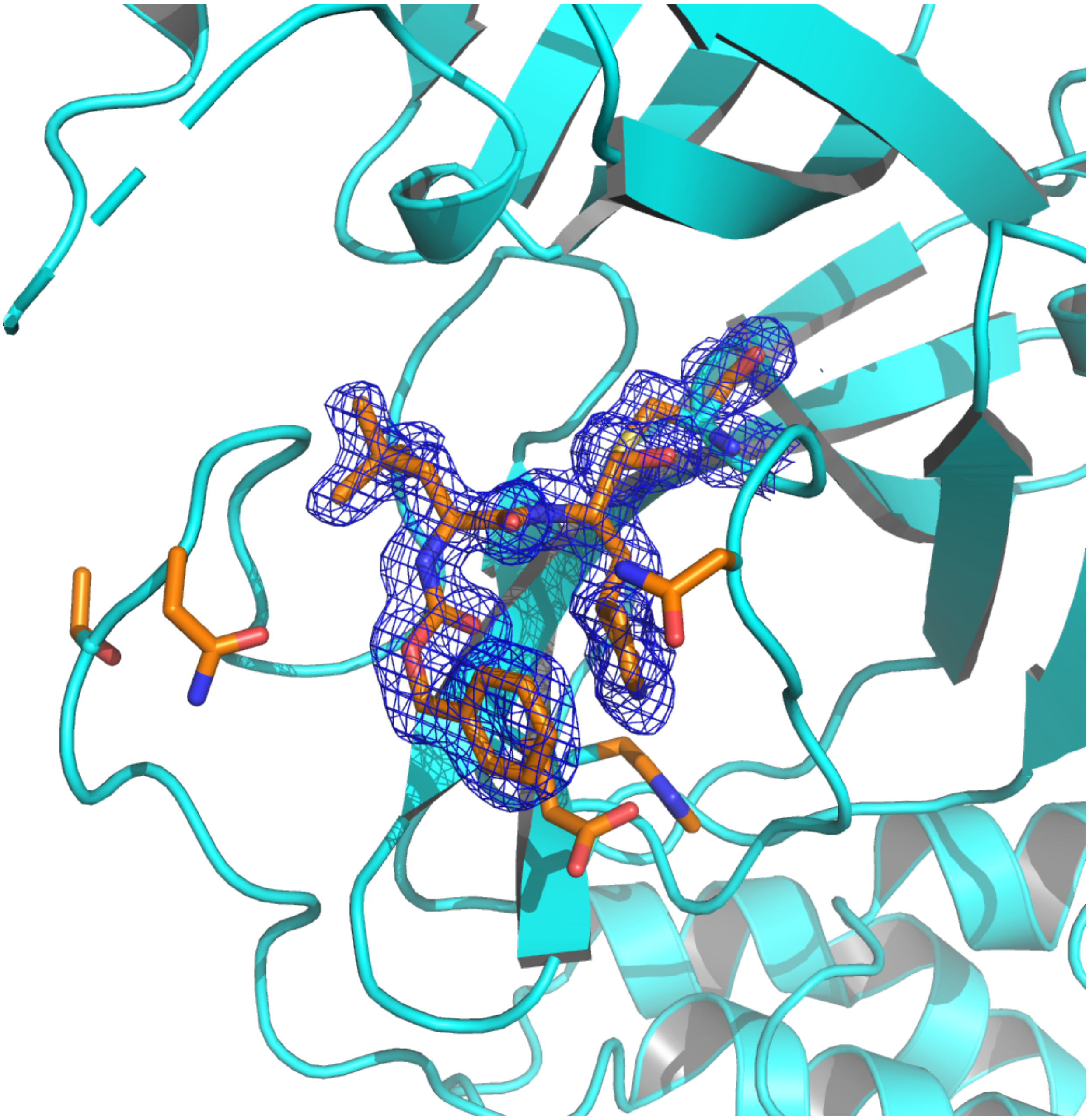

Figure S6: The 2Fo-Fc map around MPI50 and Cys145 in the active site of the M^{Pro}-MPI50 complex. The map is contoured at the 1σ level.

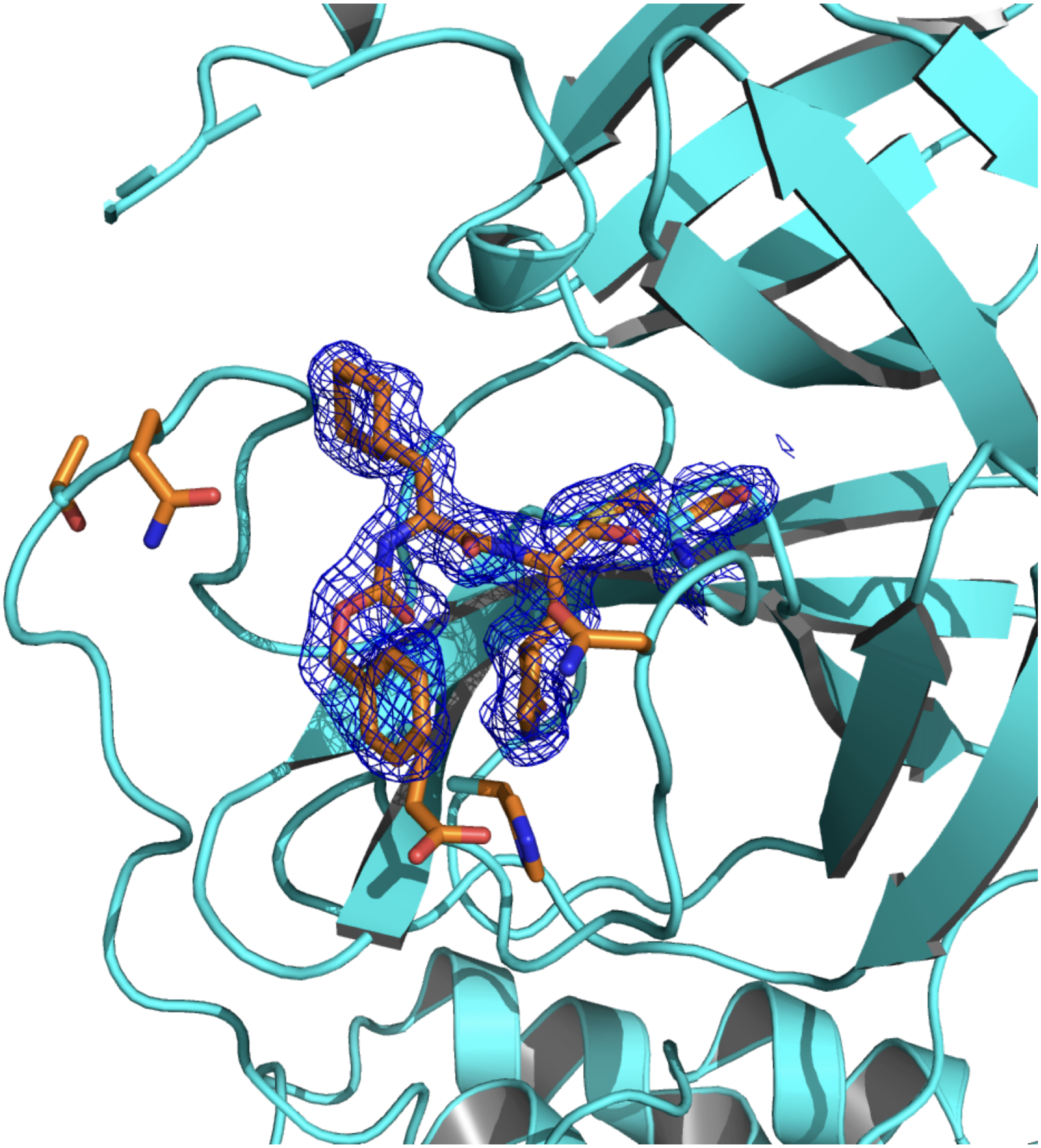

Figure S7: The 2Fo-Fc map around MPI51 and Cys145 in the active site of the M^{Pro}-MPI51 complex. The map is contoured at the 1σ level.

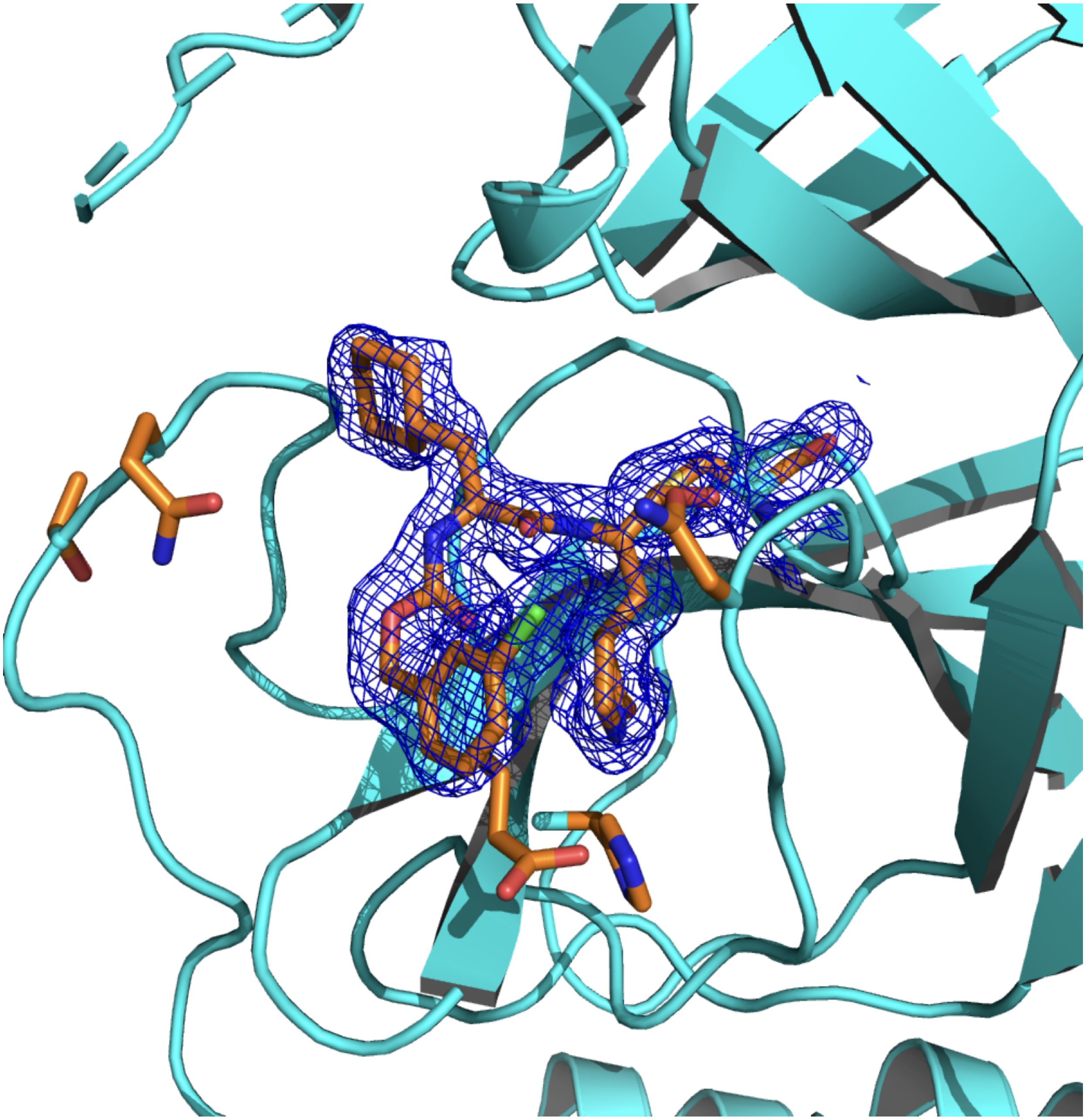

Figure S8: The 2Fo-Fc map around MPI52 and Cys145 in the active site of the M^{Pro}-MPI52 complex. The map is contoured at the 1σ level.

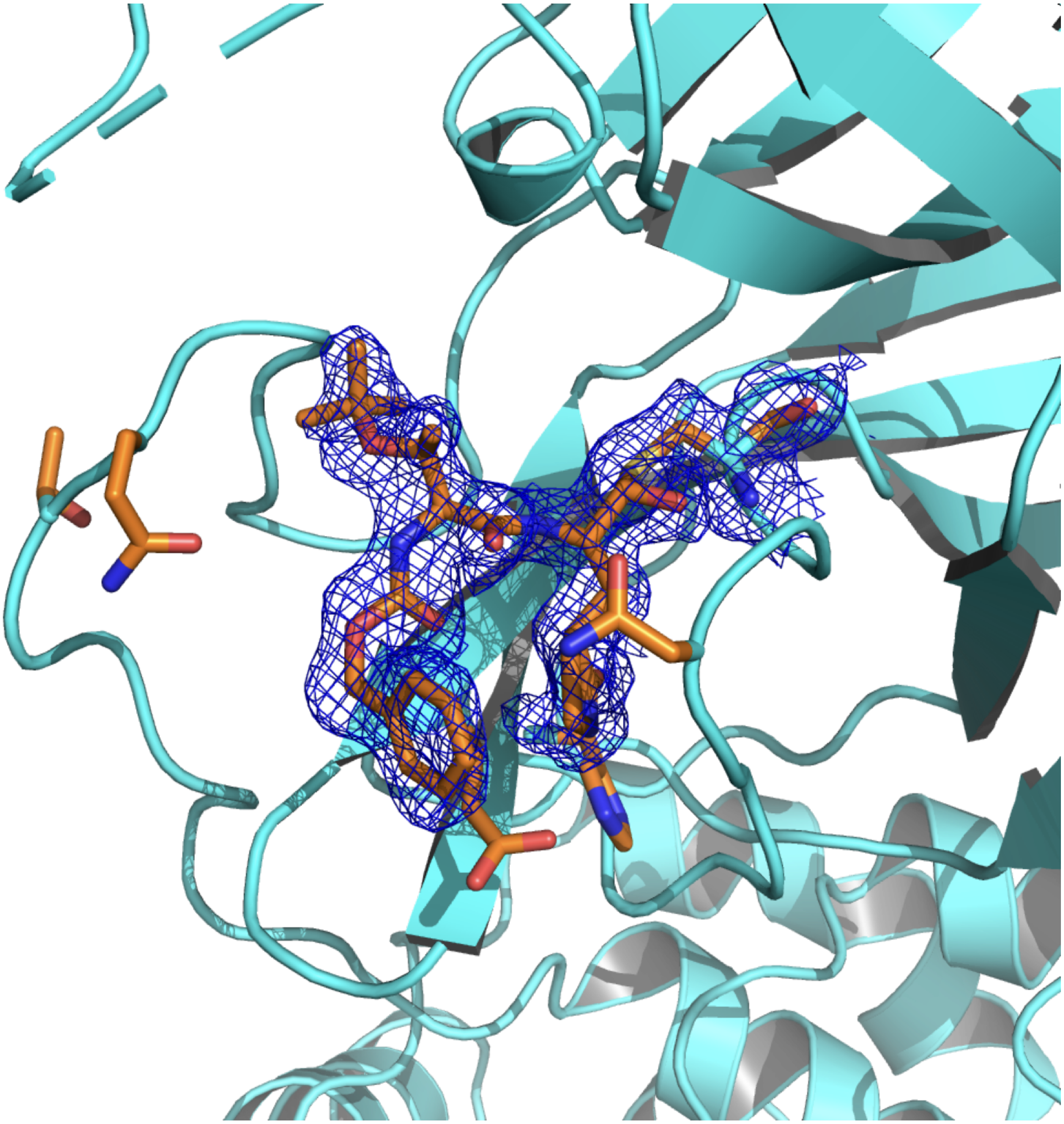

Figure S9: The 2Fo-Fc map around MPI54 and Cys145 in the active site of the M^{Pro}-MPI54 complex. The map is contoured at the 1σ level.

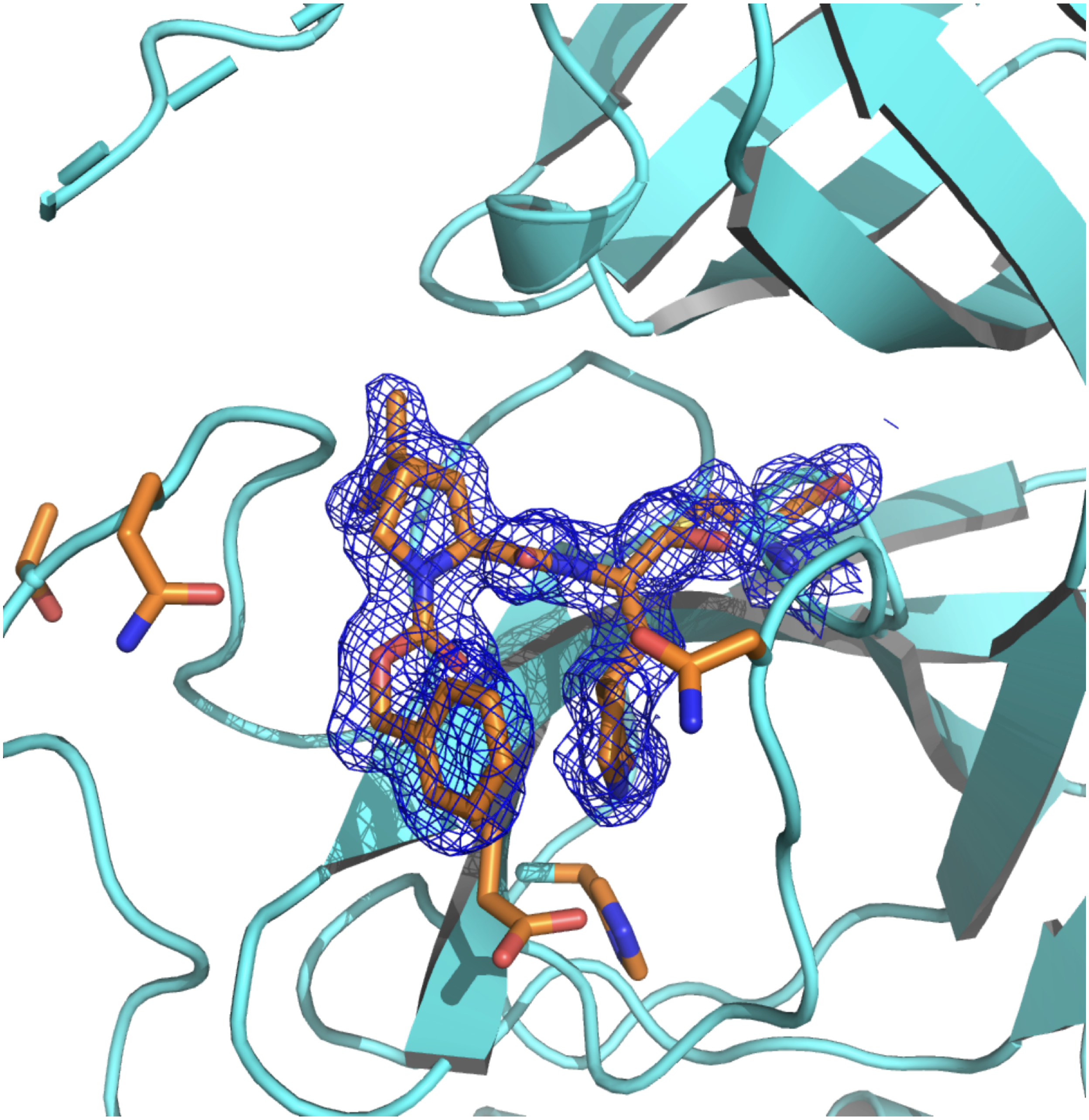

Figure S10: The 2Fo-Fc map around MPI57 and Cys145 in the active site of the M^{Pro}-MPI57 complex. The map is contoured at the 1σ level.

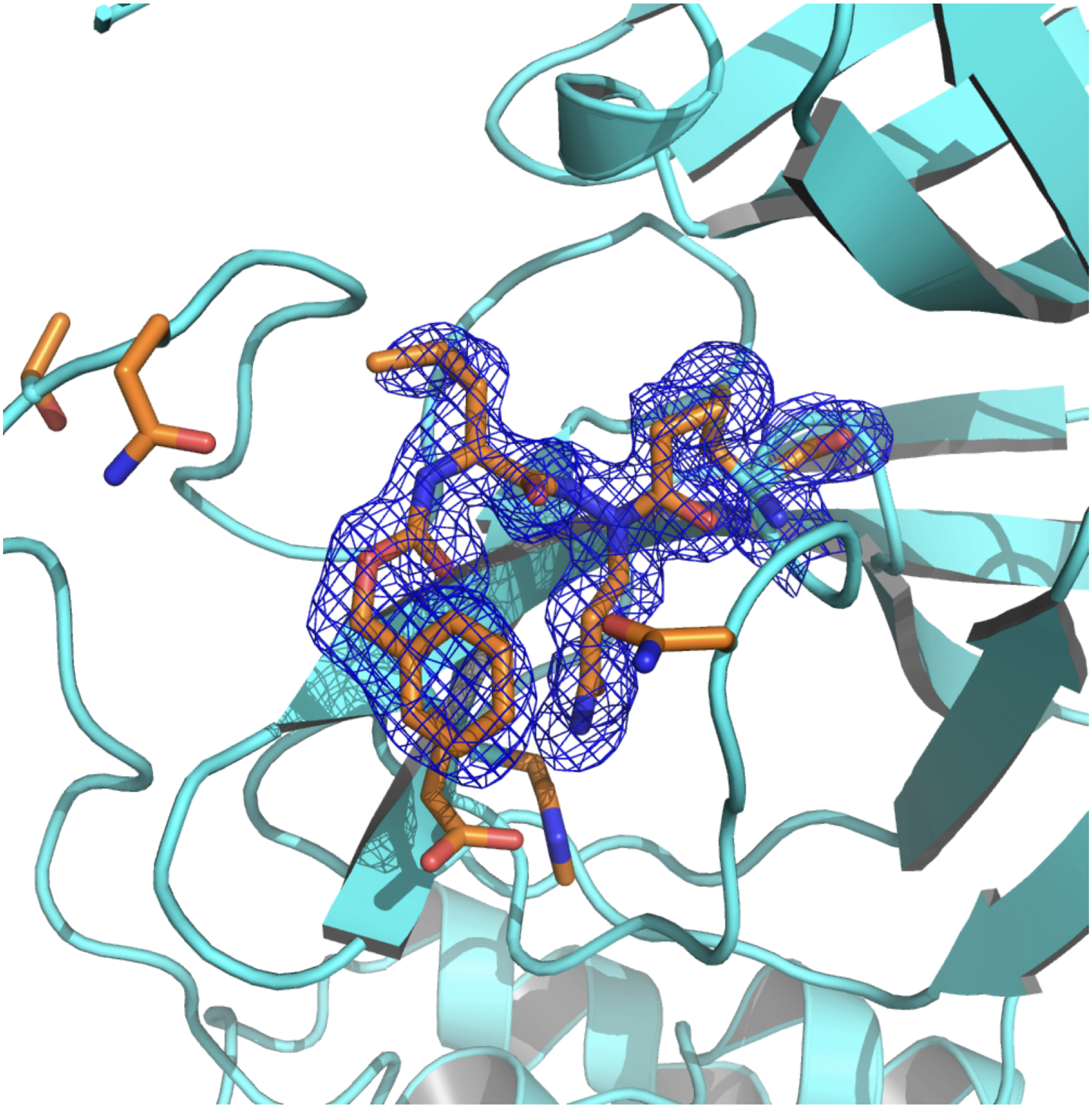

Figure S11: The 2Fo-Fc map around MPI69 and Cys145 in the active site of the M^{Pro}-MPI69 complex. The map is contoured at the 1σ level.

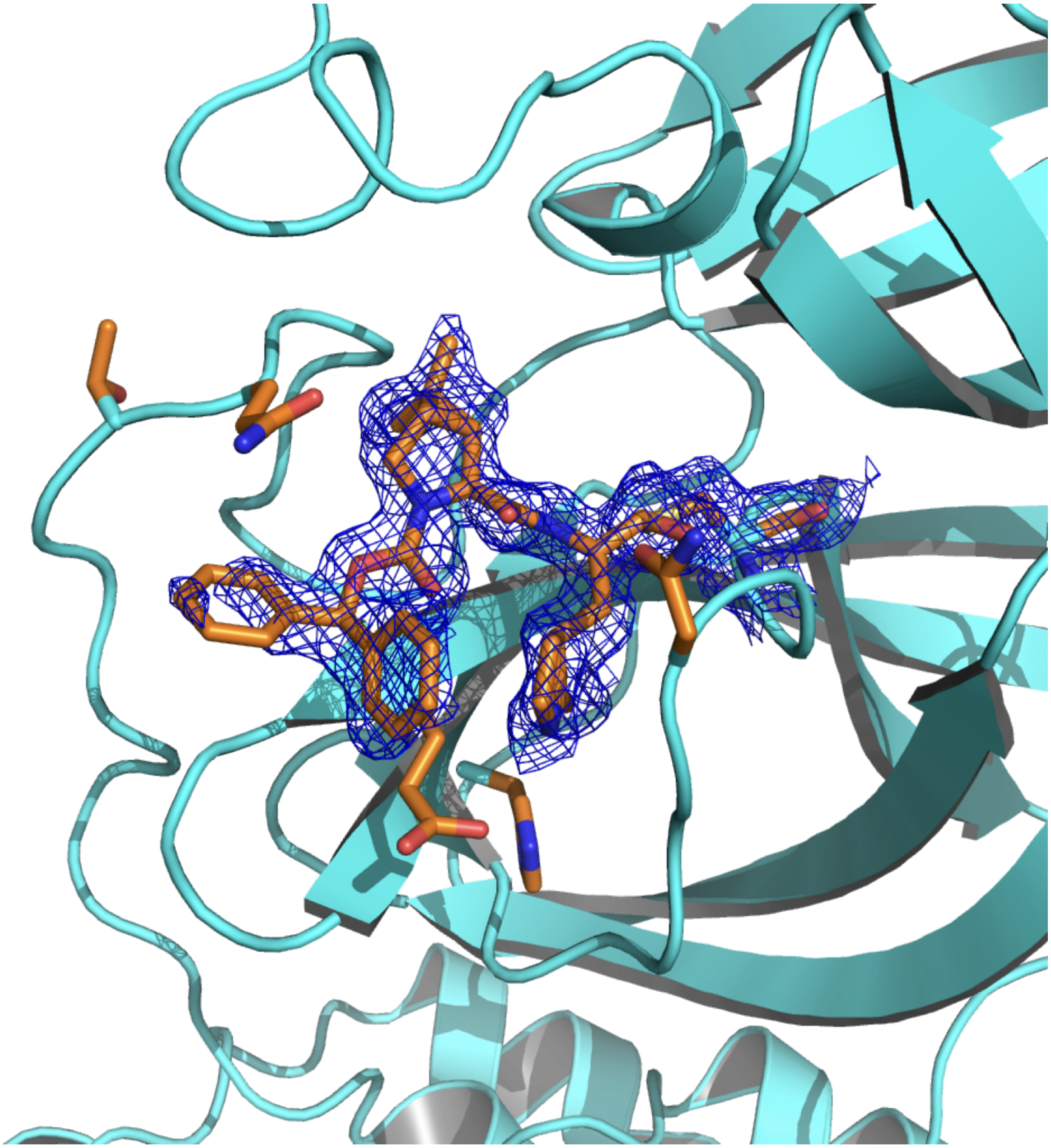

Figure S12: The 2Fo-Fc map around MPI94 and Cys145 in the active site of the M^{Pro}-MPI94 complex. The map is contoured at the 1σ level.

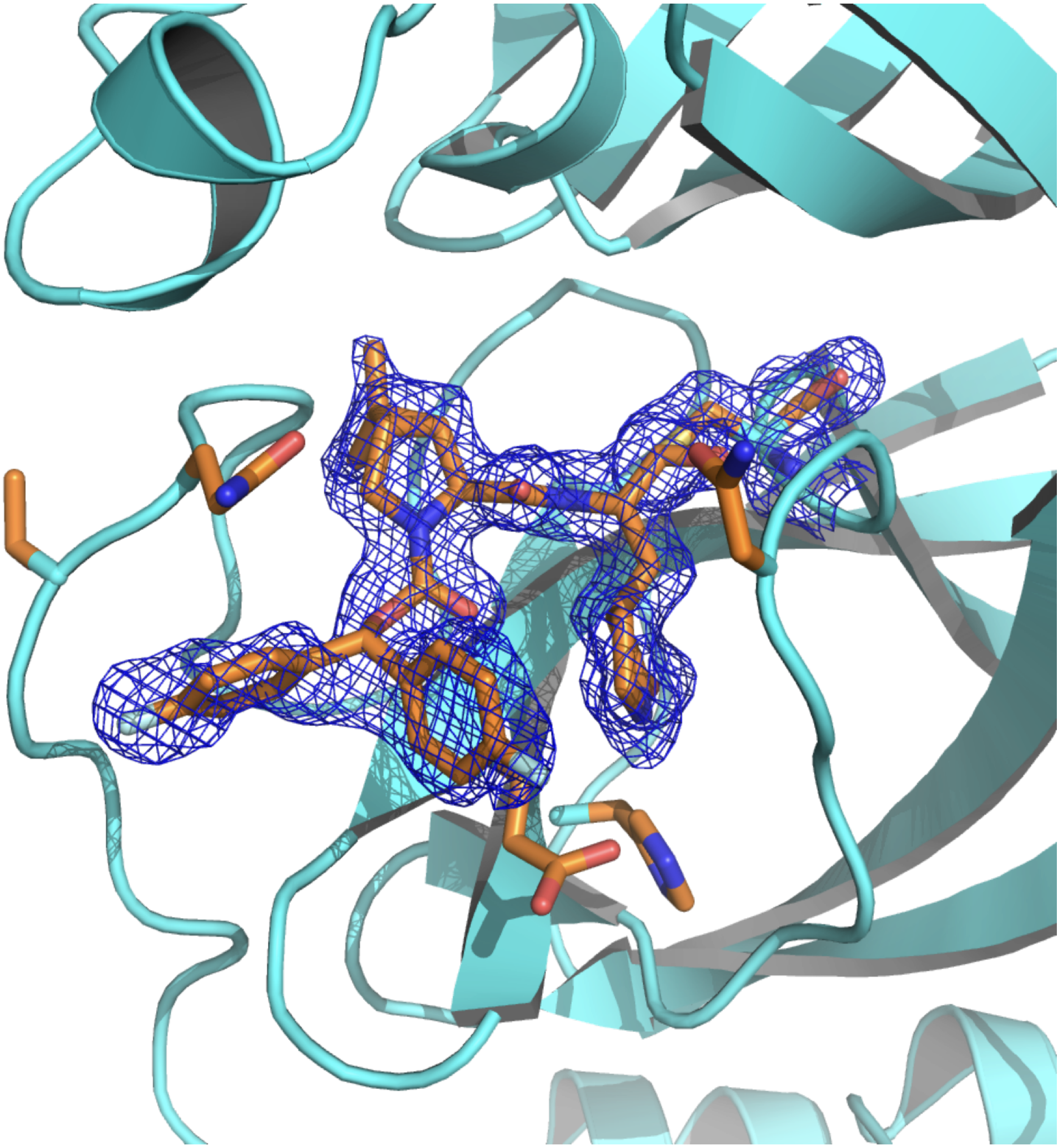

Figure S13: The 2Fo-Fc map around MPI95 and Cys145 in the active site of the M^{Pro}-MPI95 complex. The map is contoured at the 1σ level.

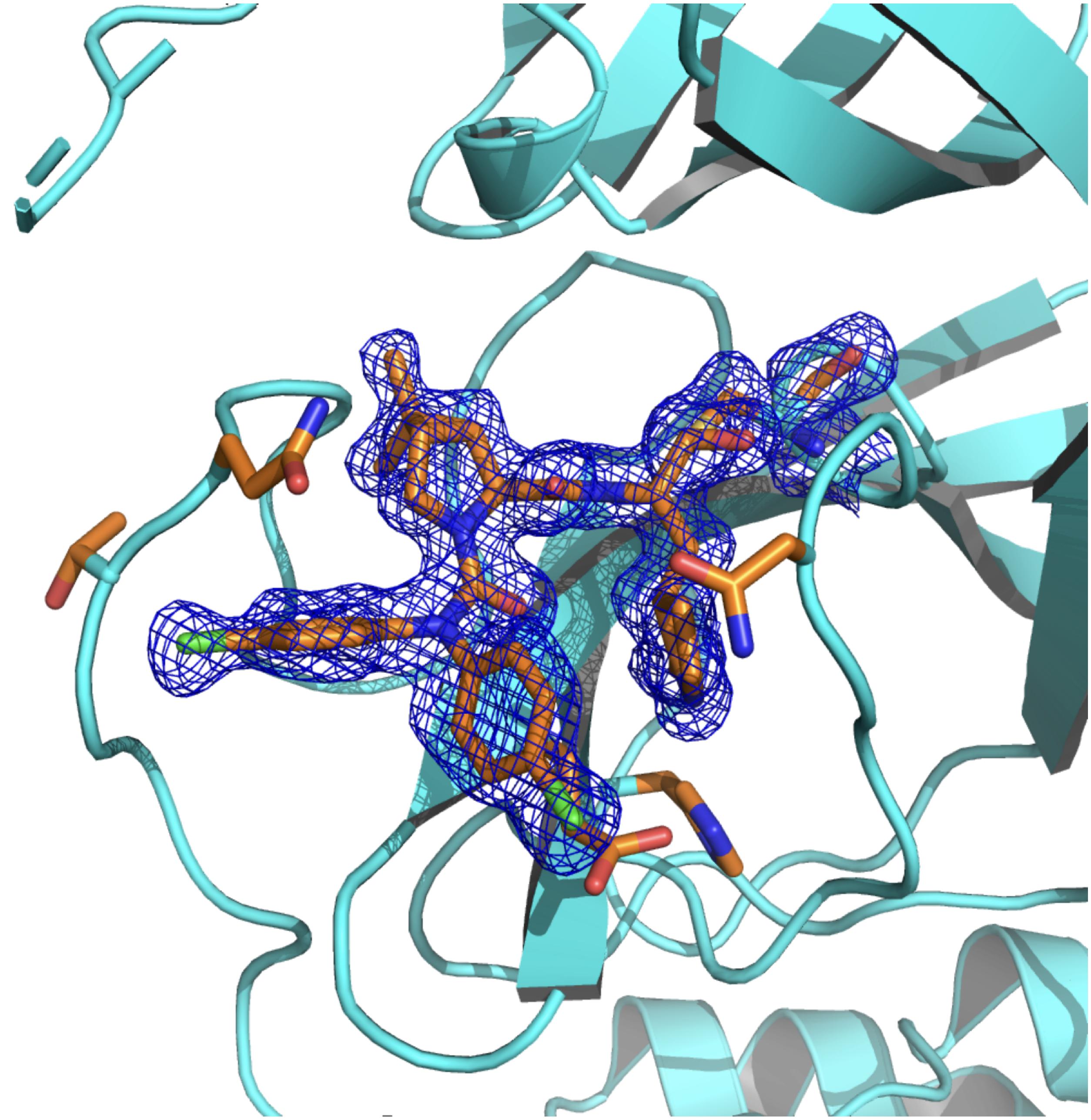

Figure S14: The 2Fo-Fc map around MPI97 and Cys145 in the active site of the M^{Pro}-MPI97 complex. The map is contoured at the 1σ level.

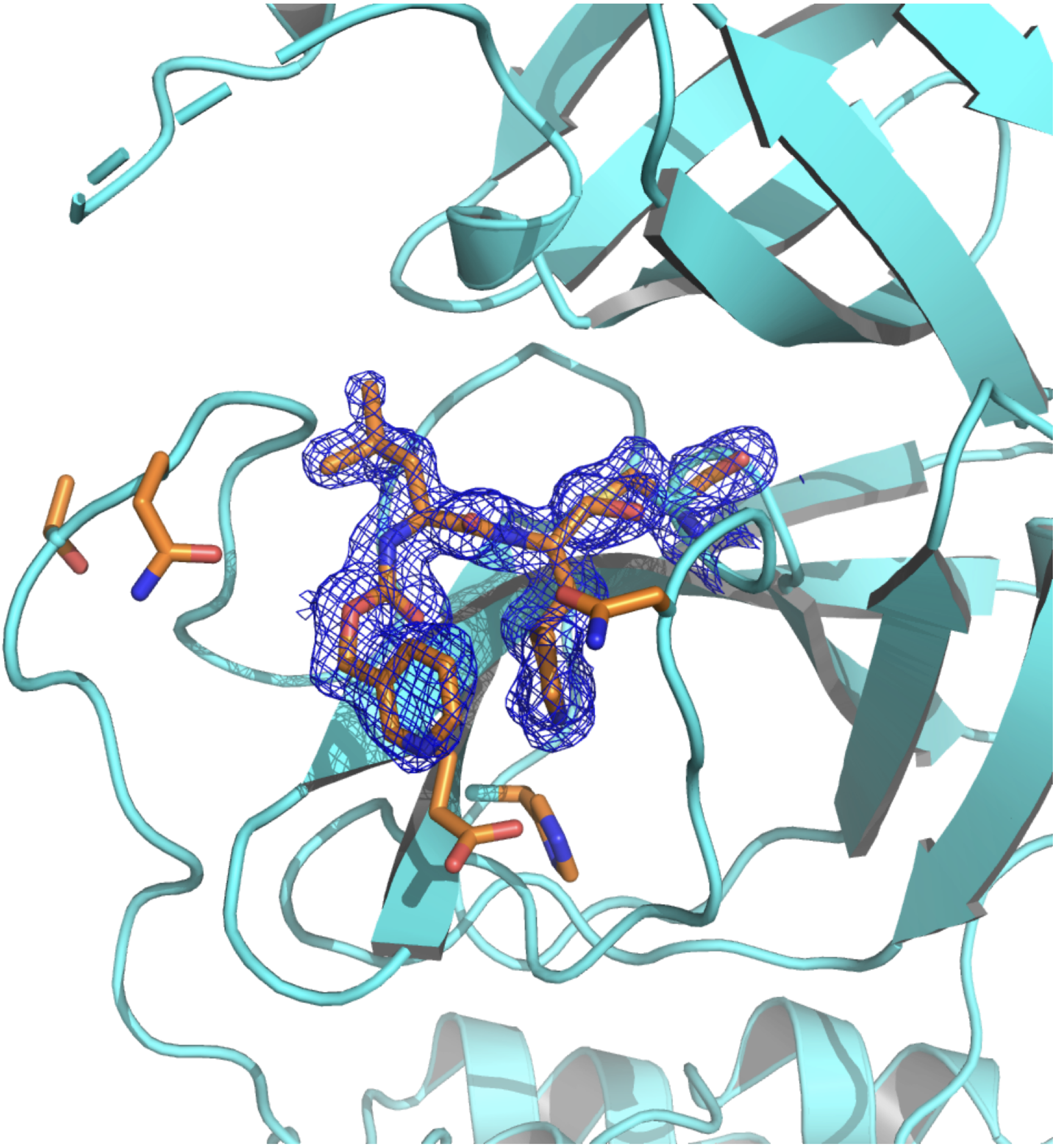

Figure S15: The 2Fo-Fc map around MPI101 and Cys145 in the active site of the M^{Pro}-MPI101 complex. The map is contoured at the 1σ level.

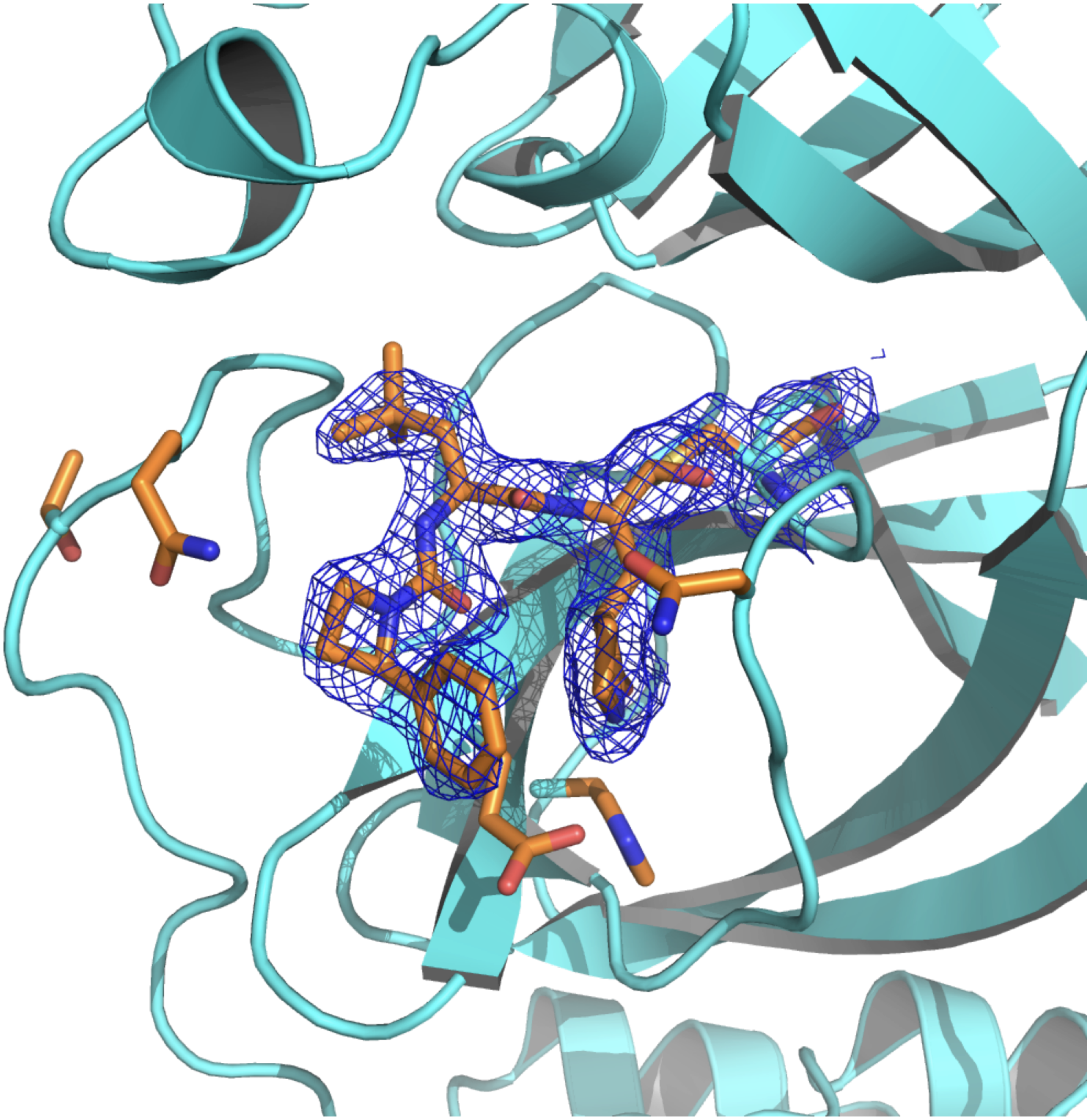

Figure S16: The 2Fo-Fc map around MPI105 and Cys145 in the active site of the M^{Pro}-MPI105 complex. The map is contoured at the 1σ level.

Supplementary Tables

Ligand (PDB Entry)	**MI-14 (8TY5)**	**MI-30 (8TY4)**	**MI-31 (8TY3)**	**MPI50 (8TQH)**	**MPI51 (8TQU)**
Data Collection					
Space Group	I121	I121	I121	I121	I121
cell dimensions					
a, b, c (Å)	51.66, 80.41, 89.86	51.71, 80.89, 90.03	51.53, 80.23, 89.34	51.82, 80.96, 90.25	54.55, 81.10, 86.91
α, β, γ (°)	90, 96.95, 90	90, 96.88, 90	90, 96.50, 90	90, 97.04, 90	90, 97.31, 90
Resolution Range (Å)	23.75 - 1.85 (1.92 - 1.85)	23.87 - 1.85 (1.92 - 1.85)	24.45 - 1.85 (1.92 - 1.85)	24.38 - 1.85 (1.92 - 1.85)	24.18 - 1.85 (1.92 - 1.85)
Unique Reflections	31118 (3126)	31343 (3114)	30875 (3102)	31170 (3124)	31907 (3142)
Completeness (%)	98.70 (98.85)	99.36 (99.11)	96.88 (99.48)	98.64 (99.94)	99.06 (98.21)
Mean I/sigma(I)	13.22 (2.35)	14.63 (2.71)	13.69 (2.55)	14.75 (4.68)	7.99 (1.41)
Wilson B-factor	20.9	19.7	20.7	13.4	20.7
R-merge	0.0289 (0.232)	0.0302 (0.239)	0.0319 (0.390)	0.0387 (0.140)	0.0659 (0.484)
R-meas	0.0409 (0.328)	0.0427 (0.337)	0.0450 (0.551)	0.0547 (0.197)	0.0932 (0.684)
R-pim	0.0289 (0.232)	0.0302 (0.239)	0.0319 (0.390)	0.0387 (0.140)	0.0659 (0.484)
CC1/2	0.999 (0.946)	0.999 (0.933)	0.999 (0.816)	0.998 (0.958)	0.996 (0.768)
Refinement					
No. Reflections	30782 (3104)	31260 (3110)	29931 (3089)	31170 (3124)	31780 (3131)
No. Reflections *R*-free	1544 (150)	1555 (166)	1477 (147)	1546 (167)	1558 (156)
R-work	0.227 (0.301)	0.220 (0.285)	0.211 (0.265)	0.207 (0.272)	0.235 (0.308)
R-free	0.253 (0.320)	0.237 (0.298)	0.224 (0.289)	0.230 (0.293)	0.269 (0.306)
No. atoms					
Non-Hydrogen	2612	2652	2614	2696	2582
Macromolecules	2363	2363	2363	2363	2363
Ligands	33	33	33	30	32
Water	216	256	218	303	187
Protein Residues	306	306	306	306	306
RMS					
Bond Length (Å)	0.009	0.010	0.009	0.009	0.009
Bond Angles (°)	1.13	1.47	1.14	1.21	1.17

Table S1. Data Collection and refinement statistics.

Ligand (PDB Entry)	**MPI52 (8TQT)**	**MPI54 (8TQL)**	**MPI57 (8TQJ)**	**MPI64 (8U9H)**	**MPI68 (7SHD)**
Data Collection					
Space Group	I121	I 1 2 1	I 1 2 1	I 1 2 1	I 1 2 1
cell dimensions					
a, b, c (Å)	54.34, 80.76, 86.14	51.59, 81.44, 89.82	51.50, 80.68, 90.23	51.87, 80.99, 90.11	53.23, 60.75, 67.65
α, β, γ (°)	90, 97.20, 90	90, 96.83, 90	90, 96.93, 90	90, 96.76, 90	90, 91.08, 90
Resolution Range (Å)	44.84 - 1.65 (1.709 - 1.65)	42.3 - 1.9 (1.97 - 1.90)	24.23 - 1.85 (1.92 - 1.85)	24.59 - 1.7 (1.76 - 1.70)	24.93 -2.27 (2.35 -2.27)
Unique Reflections	44318 (4383)	28080 (2873)	30883 (3050)	37723 (3618)	34273 (3243)
Completeness (%)	99.81 (99.48)	96.43 (99.03)	98.48 (96.87)	91.46 (87.05)	98.60 (93.11)
Mean I/sigma(I)	7.05 (0.78)	10.66 (3.21)	16.68 (4.10)	13.77 (1.20)	14.4 (1.2)
Wilson B-factor	26.0	13.3	18.2	21.0	28.2
R-merge	0.0461 (0.880)	0.0470 (0.216)	0.0246 (0.126)	0.0692 (1.35)	0.187 (0.067)
R-meas	0.0652 (1.244)	0.0665 (0.306)	0.0348 (0.175)	0.0745 (1.56)	0.225 (0.079)
R-pim	0.0461 (0.880)	0.0470 (0.216)	0.0246 (0.126)	0.0267 (0.765)	0.094 (0.034)
CC1/2	0.997 (0.480)	0.997 (0.917)	0.999 (0.975)	0.999 (0.520)	0.988 (0.995)
Refinement					
No. Reflections	44293 (4364)	28075 (2872)	30843 (3035)	37228 (3521)	34065 (3231)
No. Reflections *R*-free	2173 (229)	1370 (136)	1523 (154)	1872 (191)	1715 (164)
R-work	0.223 (0.358)	0.237 (0.316)	0.213 (0.315)	0.208 (0.321)	0.230 (0.280)
R-free	0.239 (0.357)	0.268 (0.348)	0.237 (0.326)	0.228 (0.328)	0.292 (0.336)
No. atoms					
Non-Hydrogen	2396	2617	2642	2628	2559
Macromolecules	2363	2363	2363	2363	2486
Ligands	33	32	31	31	35
Water	214	222	248	234	203
Protein Residues	306	306	306	306	306
RMS					
Bond Length (Å)	0.009	0.009	0.019	0.008	0.009
Bond Angles (°)	1.24	1.16	1.11	1.12	1.22

Table S1. Data Collection and refinement statistics (cont'd).

Ligand (PDB Entry)	**MPI69 (7SHC)**	**MPI94 (8U9K)**	**MPI95 (8U9M)**	**MPI96 (8U9N)**	**MPI97 (8U9T)**
Data Collection					
Space Group	I121	I121	I121	I121	I121
cell dimensions					
a, b, c (Å)	51.67, 81.96, 89.39	51.90, 80.75, 90.50	52.06, 80.87, 90.69	51.85, 81.02, 90.31	51.91, 80.76, 90.32
α, β, γ (°)	90, 96.92, 90	90, 96.77, 90	90, 96.66, 90	90, 96.77, 90	90, 96.86, 90
Resolution Range (Å)	30.87 - 1.55 (1.605 - 1.55)	44.93 - 1.85 (1.92 - 1.85)	47.28 - 1.85 (1.92 - 1.85)	30.72 - 1.7 (1.76 - 1.7)	29.29 - 1.65 (1.71 - 1.65)
Unique Reflections	53039 (5199)	30665 (3008)	31571 (2959)	40710 (4017)	40936 (3574)
Completeness (%)	98.90 (97.80)	96.78 (96.01)	98.71 (93.96)	99.39 (98.91)	90.90 (77.15)
Mean I/sigma(I)	11.49 (1.59)	10.05 (2.24)	14.29 (2.75)	12.11 (1.40)	14.83 (2.10)
Wilson B-factor	15.38	15.73	19.96	19.84	18.23
R-merge	0.0366 (0.486)	0.0523 (0.316)	0.0277 (0.231)	0.0814 (1.12)	0.0637 (0.789)
R-meas	0.0517 (0.687)	0.0739 (0.447)	0.0392 (0.326)	0.0868 (1.24)	0.0683 (0.900)
R-pim	0.0366 (0.486)	0.0523 (0.316)	0.0277 (0.231)	0.0291 (0.536)	0.0241 (0.423)
CC1/2	0.999 (0.757)	0.997 (0.855)	0.999 (0.932)	0.999 (0.759)	0.999 (0.989)
Refinement					
No. Reflections	52981 (5192)	30664 (3007)	31488 (2954)	40554 (4001)	40470 (3440)
No. Reflections *R*-free	2654 (292)	1527 (161)	1553 (154)	2028 (212)	1979 (169)
R-work	0.231 (0.320)	0.276 (0.375)	0.217 (0.293)	0.219 (0.320)	0.204 (0.300)
R-free	0.247 (0.350)	0.302 (0.367)	0.240 (0.303)	0.242 (0.335)	0.236 (0.319)
No. atoms					
Non-Hydrogen	2647	2610	2637	2666	2695
Macromolecules	2367	2363	2363	2363	2363
Ligands	29	37	39	38	38
Water	251	210	235	265	294
Protein Residues	306	306	306	306	306
RMS					
Bond Length (Å)	0.007	0.01	0.008	0.009	0.009
Bond Angles (°)	1.00	1.81	1.67	1.16	1.18

Table S1. Data Collection and refinement statistics (cont'd).

Ligand (PDB Entry)	**MPI98 (8U9U)**	**MPI101 (8U9V)**	**MPI105 (8U9W)**
Data Collection			
Space Group	I 1 2 1	I 1 2 1	I 1 2 1
cell dimensions			
a, b, c (Å)	51.95, 81.63, 90.15	51.56, 82.37, 89.53	51.96, 81.30, 90.81
α, β, γ (°)	90, 96.07, 90	90, 96.73, 90	90, 96.65, 90
Resolution Range (Å)	24.07 - 1.85 (1.92 - 1.85)	24.28 - 1.65 (1.71 - 1.65)	24.42 - 2.10 (2.18 - 2.1)
Unique Reflections	31779 (3136)	44018 (4322)	21964 (2188)
Completeness (%)	98.67 (98.64)	98.29 (96.23)	98.50 (98.13)
Mean I/sigma(I)	11.06 (1.24)	10.16 (1.53)	10.44 (2.09)
Wilson B-factor	23.24	16.95	27.17
R-merge	0.111 (1.39)	0.0816 (0.950)	0.112 (0.500)
R-meas	0.118 (1.53)	0.0897 (1.09)	0.120 (0.550)
R-pim	0.0385 (0.643)	0.0357 (0.524)	0.0407 (0.225)
CC1/2	0.998 (0.637)	0.998 (0.814)	0.997 (0.939)
Refinement			
No. Reflections	31558 (3126)	43930 (4286)	21658 (2147)
No. Reflections *R*-free	1563 (161)	2196 (204)	1125 (125)
R-work	0.254 (0.330)	0.223 (0.359)	0.205 (0.254)
R-free	0.285 (0.352)	0.249 (0.382)	0.234 (0.296)
No. atoms			
Non-Hydrogen	2403	2665	2586
Macromolecules	2363	2367	2367
Ligands	40	34	32
Water	250	264	187
Protein Residues	306	306	306
RMS			
Bond Length (Å)	0.01	0.009	0.008
Bond Angles (°)	1.32	1.01	0.92

Table S1. Data Collection and refinement statistics (cont'd).
